# Microcephaly-associated genes *asp* and *Sas4* influence chromatin organization and nuclear lamina structure in *Drosophila melanogaster*

**DOI:** 10.1242/dev.205125

**Published:** 2026-05-28

**Authors:** Degisew Yinur Mengistu, Marta Marzullo, Claudia Pellacani, Marcella Marchetti, Marta Terribili, Emma Montivero Morales, Maria Patrizia Somma, Laura Ciapponi

**Affiliations:** ^1^ Department of Biology and Biotechnologies ‘C. Darwin’, Sapienza University of Rome, Rome 00185, Italy; ^2^ IBPM CNR, c/o Department of Biology and Biotechnologies ‘C. Darwin’, Sapienza University of Rome, Rome 00185, Italy

**Keywords:** *Drosophila melanogaster*, Microtubule, Cytoskeleton, Nuclear lamina, Epigenetics, MCPH

## Abstract

Autosomal recessive primary microcephaly (MCPH) is a neurodevelopmental disorder characterized by reduced brain size and intellectual disability. Mutations in over 30 genes, nearly half linked to centrosome biogenesis or microtubule (MT) dynamics, highlight spindle defects in disease aetiology, yet these alone do not fully explain MCPH. Here, we show that the *Drosophila* orthologs of ASPM/MCPH5 (*asp*) and CENPJ/MCPH6 (*Sas4*) contribute to safeguard nuclear architecture and chromatin organization during brain development. Loss of either gene perturbs MT organization and centromere clustering, leading to reduced Lamin and HP1α levels, and to deformed nuclear lamina. Mutants also display a global reduction in heterochromatin-associated histone marks, H3K9me2/3 and H3K27me3, along with an increase in the euchromatin-associated mark H3K4me3 and elevated DNA damage with delayed repair. Notably, inhibiting demethylases with methylstat restores H3K9me3 and nuclear morphology. These findings suggest a previously unreported role for centrosome proteins in regulating chromatin organization, providing new insights into the mechanisms underlying MCPH pathogenesis.

## Teaser

**Highlighted Article:** *asp* and *Sas4* microcephaly-associated genes help maintain nuclear morphology and DNA stability, providing insight into mechanisms underlying autosomal recessive primary microcephaly.

## INTRODUCTION

Autosomal recessive primary microcephaly (MCPH) is a neurodevelopmental disorder present at birth and characterized by reduction of occipitofrontal head circumference (OFC) by at least three standard deviations from the mean (Mahmood et al., 2011; Thornton and Woods, 2009; von der Hagen et al., 2014; Woods et al., 2005; Zaqout and Kaindl, 2022). MCPH results from neurogenesis defects leading to a reduced number of neurons. To date, MCPH has been linked to recessive mutations in 30 genes (Siskos et al., 2021), which are expressed in all proliferating cell types that will eventually form the cerebral cortex. These genes encode proteins involved in many cellular processes that are essential for proper brain development, including the balance between proliferation and differentiation, the timely migration of neurons, and the maintenance of genome stability (Jean et al., 2020; Phan and Holland, 2021). Specifically, 12 of the 30 MCPH genes encode centrosomal components and/or regulators of MT dynamics (O'Neill et al., 2018). Centrosome defects can lead to the disruption of mitotic spindle positioning, which may result in an imbalance between symmetric and asymmetric division in neural stem cells (NSCs) (Buchman et al., 2011; Jayaraman et al., 2016; Xu et al., 2014). This imbalance would reduce the proliferation of neural progenitors and/or cause premature neuronal differentiation, which may lead to the generation of a small-sized brain. However, recent evidence indicates that altered spindle positioning during neurogenesis is not sufficient to directly induce significant changes in cell fate (Lechler and Mapelli, 2021; Li et al., 2017; Phan and Holland, 2021). Thus, mitotic defects do not fully account for the complexity of the MCPH aetiology.

DNA damage accumulation has been observed in many MCPH models, consistent with the role of several MCPH proteins in DNA repair and genome stability (Iegiani et al., 2023). However, the pool of neural progenitor cells could also be significantly depleted by modifying the fate of proliferating NSCs and inducing their premature differentiation. This might be due to the premature activation of lineage-specific genes and the concomitant silencing of stem cell genes. The establishment of such a transcriptional program must be tightly coordinated with cell division and might be accompanied by changes in chromatin organization. Recently, a great emphasis has been placed on the impact of post-translational histone modifications on the spatiotemporal gene expression programs during neurogenesis (Adam and Harwell, 2020). The N-terminal histone tail is subjected to many modifications (including lysine acetylation, lysine and arginine methylation, serine/threonine phosphorylation, etc.) that regulate the accessibility of the associated DNA regulatory elements. Specifically, methylation of histone H3 on lysines 4, 36, 79 (H3K4, H3K36 and H3K79) is generally associated with poised or active gene transcription, whereas methylation of histone H3 on lysine 9, 20 and 27 (H3K9, H3K20 and H3K27) is a hallmark of gene silencing in the heterochromatic regions. Among those histone repressive marks, H3K27me3 is crucial for the regulation of the progression of NSC lineages (Abdusselamoglu et al., 2019).

Emerging evidence suggests that mechanical forces from the cytoskeleton contribute to the regulation of chromatin organization and nuclear function during neurogenesis. Interestingly, recent work suggests that chromatin organization is affected by forces exerted by the cytoskeleton on the nucleus (Shokrollahi and Mekhail, 2021; Uhler and Shivashankar, 2017). The connection between the nucleus and the cytoplasm is mediated by the MT-actin cytoskeleton and components of the nuclear membrane, such as the LINC complex and the Lamin nucleoskeleton (Amiad Pavlov et al., 2025; Gimpel et al., 2017; Lansweers et al., 2025; Padilla et al., 2025; Sun et al., 2019). The Lamin meshwork is essential to establish the 3D architecture of the genome by anchoring heterochromatic regions to the nuclear envelope (NE, Camozzi et al., 2014). The resulting chromatin compartmentalisation is required for the regulation of many nuclear processes, such as transcriptional activation and repression, the DNA damage response and chromatin dynamics, during the cell cycle. Lamin dysfunction causes a heterogeneous group of diseases known as laminopathies (Evangelisti et al., 2022). A common feature of laminopathy cells is the presence of misshaped nuclei and heterochromatin loss. The correlation between altered MT cytoskeleton, heterochromatin remodelling and Lamin dysfunction has been demonstrated in a *Drosophila* tauopathy model, in which the aberrant cytoskeletal-nucleoskeletal interaction leads to the heterochromatin relaxation, and loss of H3K9me2 and HP1α, which promotes neurodegeneration (Frost et al., 2014, 2016).

The *ASPM/MCPH5* (abnormal spindle-like microcephaly-associated) gene, the human ortholog of the *Drosophila asp* gene, has been found to be mutated in over 40% of MCPH cases. Asp/ASPM proteins localize to the MT minus ends (Jiang et al., 2017) and play a crucial role in the regulation of mitotic spindle assembly and orientation (Razuvaeva et al., 2023). ASPM is a component of a protein complex required for centriole biogenesis and activity (Schoborg et al., 2015). This complex is formed by WDR62, CEP63 and CENPJ, all of which are proteins associated with microcephaly (Jayaraman et al., 2016). Although Asp/ASPM was initially thought to function exclusively during mitosis, recent studies have revealed its involvement in interphase processes such as DNA repair and protein ubiquitylation (Buchman et al., 2011; Capecchi and Pozner, 2015; Williams et al., 2015).

The centromere-associated protein J (*CENPJ/MCPH6*) gene, the human ortholog of *Drosophila Sas4* (Spindle Assembly Abnormal 4), encodes a centrosomal protein involved in centriole duplication and centrosome assembly. CENPJ/Sas4 mediates the interaction between the pericentriolar material (PCM) scaffold and the centriole during centrosome biogenesis by providing a platform termed ‘extended surface-like’ (Gartenmann et al., 2020; Zheng et al., 2014). Depletion of mouse CENPJ protein leads to loss of centrosomes and defective brain development (Gabriel et al., 2016; McIntyre et al., 2012).

In this study, we investigated whether cytoskeletal organization and nuclear morphology are affected in *Drosophila* models of MCPH carrying mutations in *Sas4* or *asp* gene. We found that loss of Asp or Sas4 function, in addition to the established effects on spindle assembly and mitotic progression, leads to alterations in nuclear architecture, chromatin organization and hypersensitivity to DNA damage during *Drosophila* neurogenesis. Altogether, these findings suggest that centrosome dysfunction can influence not only the mitotic process but also the architecture and morphology of the nucleus, thereby contributing to the balance between proliferation and differentiation during brain development.

## RESULTS

### Cytoskeleton defects in *asp* and *Sas4* mutant brain cells

To explore the connection between cytoskeleton and nuclear architecture, we examined the microtubule network in interphase neuroblasts (NBs) from *Sas4* or *asp* homozygous mutants (*Sas4^s2214^* and *asp^t25^*) as well from brains subjected to RNAi knockdown (all experiments involving RNAi lines are included in the supplementary information). Neuroblasts were identified based on a nuclear diameter >10 µm, as consistently observed in Deadpan-positive cells (Fig. S1).

**Fig. 1.**
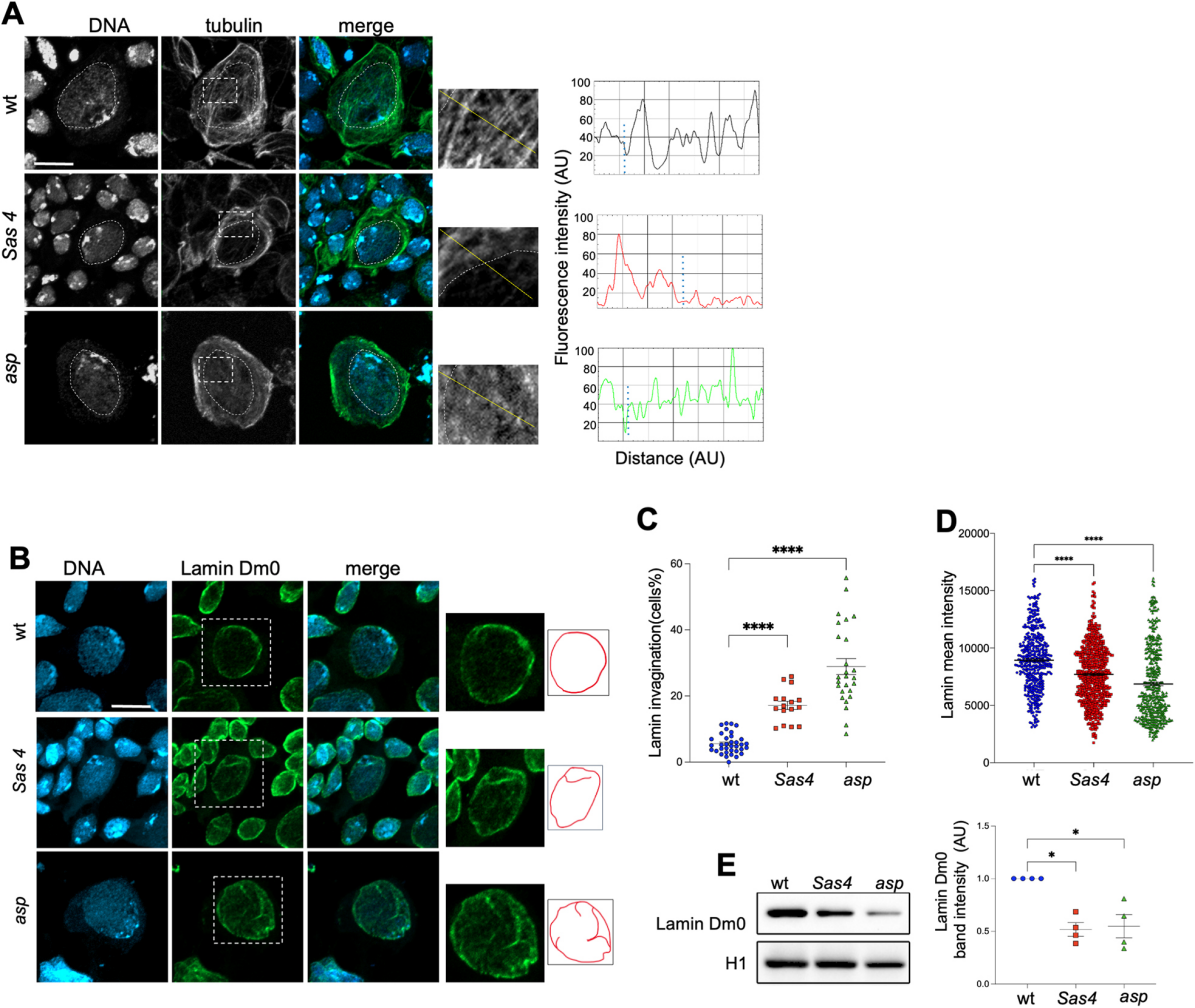
**Cytoskeleton defects in *asp* and *Sas4* mutant brain cells.** (A) Interphase neuroblasts from third instar brains of wild-type and *Sas4* or *asp* mutant *Drosophila* immunostained with anti-tubulin antibody (green; mouse anti-tubulin) and DAPI (blue, DNA, dashed circles). Dotted rectangles outline the regions shown on the right, with corresponding tubulin intensity profiles (black for wild type, red for *Sas4* and green for *asp* mutants) across the highlighted segment. (B) Neuroblasts of wild type and *Sas4* or *asp* mutants immunostained with anti-LaminDm0 antibody (green) and DAPI (blue, DNA). Dashed squares outline the regions shown on the right (three times higher magnification), with the corresponding illustrative rendering of the LaminDm0 signal. (C) Graphical representation of the percentage of neuroblasts showing invaginations of the nuclear envelope in *Sas4* (red squares) and *asp* (green triangles) mutant, and wild-type (blue circles) cells. Cells with Lamin invaginations were defined as those in which the nuclear lamina folds or protrudes inward into the nuclear interior, as highlighted by the illustrative rendering. Each dot represents the score of cells per 63× microscope field in three brains per genotype (*n*≥20). (D) Quantification of Lamin fluorescence intensity per cell, as in C; each dot represents a single cell from at least three brains (*n*≥200). (E) Representative immunoblots on protein extracts from wild-type, *Sas4* or *asp* larval brains labelled with anti-LaminDm0 antibody, with the corresponding band quantification normalized to the loading control (H1) in at least three independent experiments. AU, arbitrary unit. Data are mean±s.e.m. *P<0.05; ****P<0.0001 (C and D, ordinary one-way ANOVA test; E, Kruskal-Wallis ANOVA test). Scale bars: 10 μm. All images are maximum-intensity projections.

In wild-type interphase cells, the centrosome is easily identifiable as a discrete focus located close to the nucleus and serves as the primary site of MT nucleation (Hannaford and Rusan, 2024). From this site, long MTs radiate outward to form a well-organized network that spans the entire cell. In *Sas4* mutant NBs, however, centrioles are entirely absent and centrosomes cannot be detected (Basto et al., 2006). Consequently, MTs are arranged in bundles restricted to the cell periphery and around the nucleus, rather than forming an integrated network (Fig. 1A and Fig. S2A), a phenotype resembling that observed in neurons following *CENPJ* silencing (Garcez et al., 2015). By contrast, in *asp* mutant NBs, centrosomes are retained during interphase, and MTs are initially nucleated from these organelles (do Carmo Avides and Glover, 1999; Gonzalez et al., 1990; Rujano et al., 2013; Wakefield et al., 2001). However, the MT network is disorganized and composed of short and thin filaments (Fig. 1A and Fig. S2A). The line-scan intensity profile analyses provide a clear visualization of these differences: wild-type NBs show defined intensity peaks corresponding to well-organized MT bundles; *Sas4* mutant NBs lack multiple peaks, with signal localized around the nucleus and cell periphery; and *asp* mutant NBs, display a high fluorescence baseline and no prominent intensity peaks, reflecting their poorly organized MT network (Fig. 1A).

Considering the contribution of actomyosin-generated forces to nuclear mechanics, we also examined whether the organization of the actomyosin network is altered in *asp* and *Sas4* mutant cells. Using an antibody against the non-muscle myosin 2 heavy chain protein (Royou et al., 2002), we detected defects in actin-myosin organization in both mutant brain cells. Line-scan analysis in neuroblasts showed that myosin peaks in wild-type cells were broader and more uniform, whereas in *Sas4* and *asp* mutants they were narrower and unevenly distributed, suggesting an altered organization. Line-scans performed on brain cell monolayers further revealed a significant reduction in myosin signal (Fig. S3A,B). These abnormal myosin patterns paralleled the defects observed in MT organization, supporting coordination between these two cytoskeletal components.

To determine whether the loss of Sas4 or Asp alters the morphology of the Lamin-based nucleoskeleton, we performed immunostaining for LaminDm0, the *Drosophila* ortholog of human Lamin B. We showed that, in both *Sas4* and *asp* mutants, a significant percentage of neuroblasts displayed a disorganization of the Lamin network, characterized by Lamin invaginations, defined as deformations of the NE in which the nuclear lamina fold or protrude inward into the nuclear interior (Paonessa et al., 2019; Fig. 1E,F, Figs S2B and S5A-C). Similar immunofluorescence experiments using an anti-Lamin C antibody revealed the same altered nuclear morphology in mutant cells (Fig. S4A,B), supporting the conclusion that the invaginations reflect deformation of the NE, as Lamin staining provides a reliable read-out of nuclear shape. This abnormal nuclear morphology phenotype was associated with significant reduction in Lamin protein levels, as evidenced by decreased Lamin immunofluorescence intensity in mutant brain cells and confirmed by western blotting (Fig. 1G,H). Collectively, our data reveal that Sas4 and Asp are important for maintaining the organization of the nuclear envelope.

### Loss of Asp and Sas4 affects heterochromatin marks and leads to nuclear envelope defects in larval brain cells

**Fig. 2.**
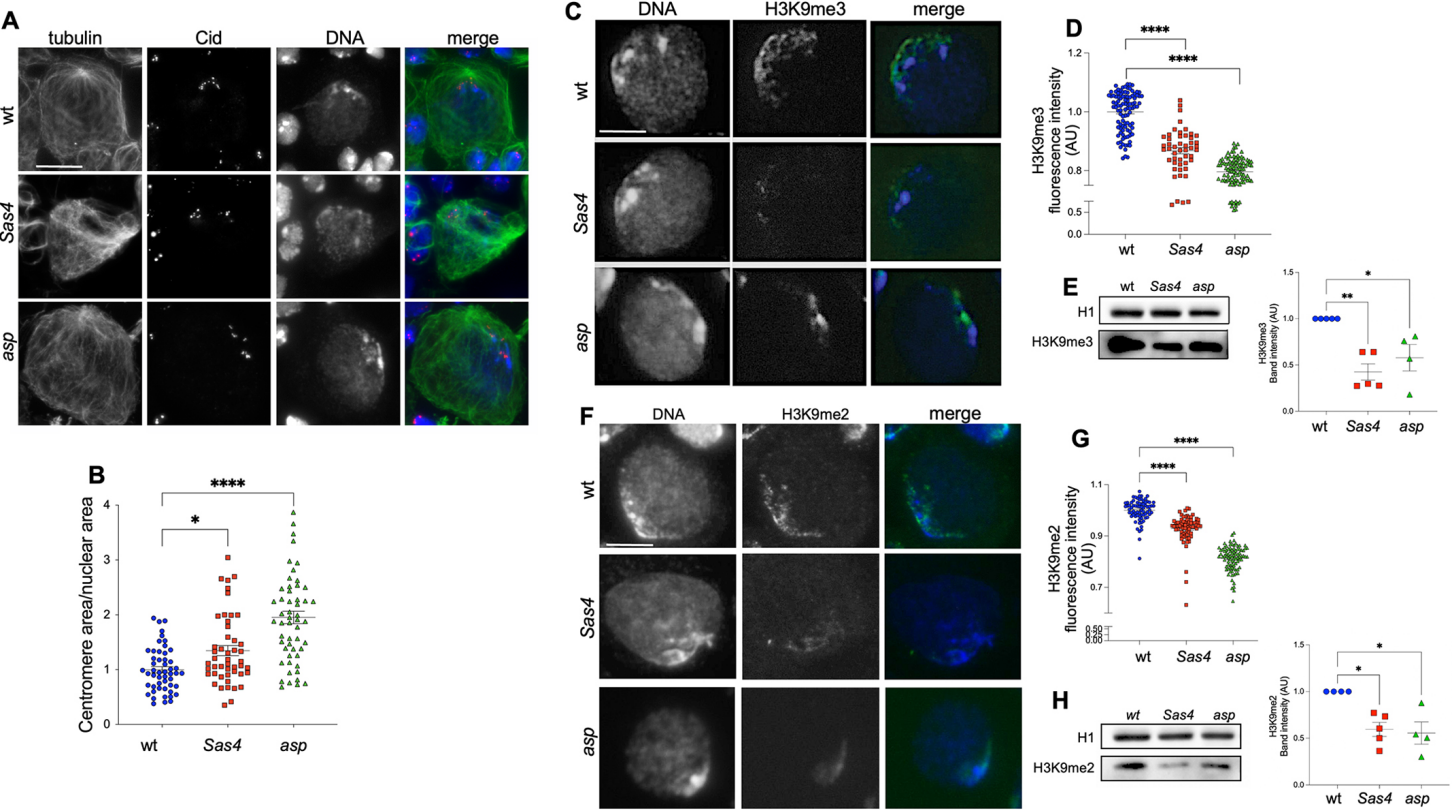
**Asp and Sas4 are required for centromere clustering and H3K9me2-3 positioning.** (A) Late G2/early prophase neuroblasts from third instar brains of wild type and *Sas4* or *asp* mutants immunostained with the anti-centromere specific Histone-3 variant marker Cid (red) and anti-tubulin (green) antibodies. DNA is in blue (DAPI). (B) Graphical representation of the centromere area expressed as the ratio between the area occupied by Cid signals and the total nuclear area in brain cells of wild type (blue circles) and *Sas4* (red squares) or *asp* (green triangles) mutants. Each dot represents a single nucleus from at least three brains (*n*≥50). (C) Neuroblast of wild-type and *Sas4* or *asp* mutants stained with anti*-*H3K9me3 (green) antibody and DAPI (blue, DNA). (D) Quantification of H3K9me3 fluorescence intensity per cell as in C, each dot represents a single cell from at least three brains (*n*≥50). (E) Representative immunoblots showing decreased levels of H3K9me3 in larval brain extracts from *Sas4* or *asp* mutants, compared to wild type with the corresponding quantification of relative band intensity normalized to the loading control (H1), in at least three independent experiments. (F) Neuroblasts of wild type and *Sas4* or *asp* mutants stained with anti-H3K9me2 (green) antibody and DAPI (blue, DNA). (G) Quantification of H3K9me2 fluorescence intensity as in F; each dot represents a single cell from at least three brains (*n*≥50). (H) Representative immunoblots showing decreased levels of H3K9me2 in larval brain extracts of *Sas4* or *asp* mutants compared to wild type, with the corresponding quantification of relative band intensity normalized to the loading control (H1) in at least three independent experiments. AU, arbitrary unit. Data are mean±s.e.m. *P<0.05; **P<0.01; ****P<0.0001 (D and G, ordinary one-way ANOVA test; B, E and H, Kruskal-Wallis ANOVA test). Scale bars: 10 μm.

To assess whether the Lamin defects in *Sas4* and *asp* mutants lead to altered heterochromatin positioning, we analysed the localization of the centromere-specific H3 variant (CENP-A; Centromere identifier, Cid in flies) in early prophase, when the spindle microtubules start to be nucleated. It is known that Cid signals are clustered near the apical centrosome in wild-type NBs (Sunchu et al., 2022). Early prophases in *asp* NBs were identified not only by the size but also by the initial assembly of asters and the presence of Cid doublets belonging to sister chromatids. In contrast, in *Sas4* mutants that lack centrosomes and asters, prophase NBs were identified solely based on the size and the presence of Cid doublets. We found that centromere clustering was reduced in both *asp* and *Sas4* mutant cells, as indicated by an increased ratio of the area occupied by Cid signals to total nuclear area (Fig. 2A,B).

Since Lamin invaginations are frequently associated with heterochromatin relaxation in human cells (Biedzinski et al., 2020; Frost et al., 2016), we examined the distribution of H3K9me2 and H3K9me3, two key epigenetic marks of gene silencing that guide the peripheral positioning of silent chromatin. Immunostaining of neuroblasts from *asp* and *Sas4* mutants, and fluorescence quantification showed a significant reduction in methylation levels compared to controls, without major changes in the peripheral localization of the remaining heterochromatin (Fig. 2C,D,F,G and Fig. S6A,B), confirmed by western blotting (Fig. 2E,H).

**Fig. 3.**
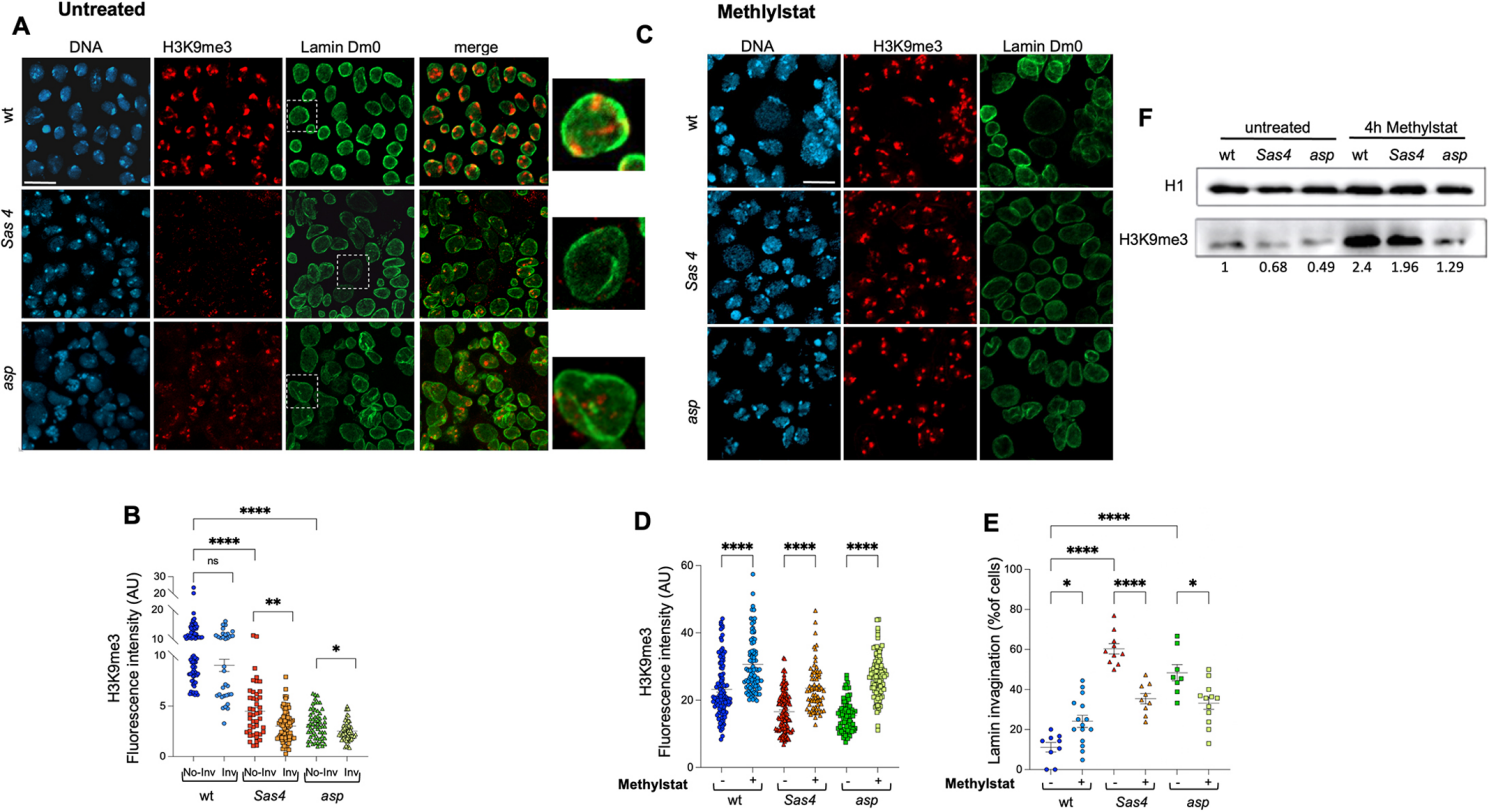
**Lamin invaginations and Lamin level reduction in *Sas4* and *asp* mutant brain cells are dependent on H3K9me3 levels.** (A) Third instar brains of wild type and *Sas4* or *asp* mutants immunostained with anti-LaminDm0 (green) and H3K9me3 (red) antibodies, and DAPI (blue, DNA). Dashed squares outline the brain cells shown on the right (three times higher magnification). Scale bar: 10 μm. (B) Fluorescence intensity quantification of H3K9me3 signal in cells with normal nuclear envelope (NE) or in cells with Lamin invaginations in *Sas4* mutant (red and orange squares), *asp* mutant (green and light-green triangles) and wild type (blue and light-blue circles) showing a significant correlation between Lamin invaginations and H3K9me3 reduction. Each dot represents a single cell (*n*≥30). (C) Third instar brains of wild type and *Sas4* or *asp* mutants immunostained with anti-LaminDm0 (green) and H3K9me3 (red) antibodies, and DAPI (blue, DNA) after 4 h treatment with 3μM methylstat. Scale bar: 10 μm. (D) Fluorescence intensity quantification of H3K9me3 in *Sas4* mutant (red and orange squares) and *asp* mutant (green and light-green triangles), and wild type (blue and light-blue rounds) untreated cells or upon 4 h treatment with 3 μM methylstat. Each dot represents a single cell from at least three brains per genotype (*n*≥50). (E) Graphical representation of the percentage of brain cells showing NE in *Sas4* (red and orange squares) or *asp* mutants (green and light-green triangles) and wild type (blue and light-blue circles) either untreated or treated with 3 μM methylstat for 4 h. Each dot represents the score of cells per 63× microscope field in at least three brains per genotype (*n*≥8). (F) Representative immunoblots on protein extracts from wild-type, *Sas4* or *asp* larval brains either untreated or treated with 3 μM methylstat for 4 h**,** labelled with anti-LaminDm0, numbers below indicate the corresponding band quantification normalized to the loading control (H1). AU, arbitrary unit. Data are mean±s.e.m. *P<0.05; **P<0.01; ****P<0.0001 (B, Mann-Whitney test; D and E, ordinary one-way ANOVA test). All images are maximum-intensity projections.

A reduction of H3K9me3 was also observed in differentiated cells of the larval brain (Fig. 3A), with the expected differences in heterochromatin localization between stem cells and terminally differentiated cells. Specifically, neuroblasts exhibit a single heterochromatin mass localized at the nuclear periphery (Fig. 2C), whereas differentiated cells show multiple heterochromatin blocks distributed across both peripheral and internal nuclear regions (Fig. 3A).

Remarkably, in mutant brains co-stained with LaminDm0 and H3K9me3, mutant cells with NE invaginations consistently displayed lower overall H3K9me3 levels compared to mutant cells without invaginations (Fig. 3A,B). H3K9me3 signal was quantified at the whole-cell level, distinguishing cells with Lamin invaginations from those without, rather than specifically at the invagination sites (Fig. 3A,B), thereby indicating a reduction of the H3K9me3 mark at the whole-cell level. Notably, *ex vivo* treatment of mutant larval brains with methylstat, a demethylase inhibitor (Hervé et al., 2025), restored H3K9me3 level (Fig. 3C-F) and significantly reduced the Sas4- or Asp-dependent nuclear invagination phenotype (Fig. 3C,E), suggesting that global chromatin methylation is required to maintain proper nuclear architecture and prevent NE deformations in these mutants.

To address whether restoration of H3K9 methylation could ameliorate the phenotype caused by Asp or Sas4 depletion *per se*, we overexpressed the *Su(var)3-9* methyltransferase in *asp* or *Sas4* RNAi lines using tubulin-GAL4, and assessed H3K9me3 levels and nuclear invaginations. In the tubulin-GAL4-driven UAS*-Su(var)3-9;*UAS*-aspRNAi* combination, as expected we observed a robust restoration of H3K9me3 levels; however, this was not accompanied by a reduction in the frequency of nuclear invaginations (Fig. S7). Unfortunately, we were unable to obtain viable individuals carrying both the UAS-*Su(var)3-9* overexpression construct and the UAS-*Sas4^RNAi^* transgene, even in the absence of GAL4-driven expression. This suggests an unexpected genetic incompatibility or background-dependent lethality associated with the combined presence of these transgenes, rather than an effect of transgene induction. Despite this technical limitation, the results from the UAS-*Su(var)3-9^OE^*;UAS-*asp^RNAi^* analysis provide evidence that restoring H3K9 methylation is not sufficient to prevent nuclear invaginations.

**Fig. 4.**
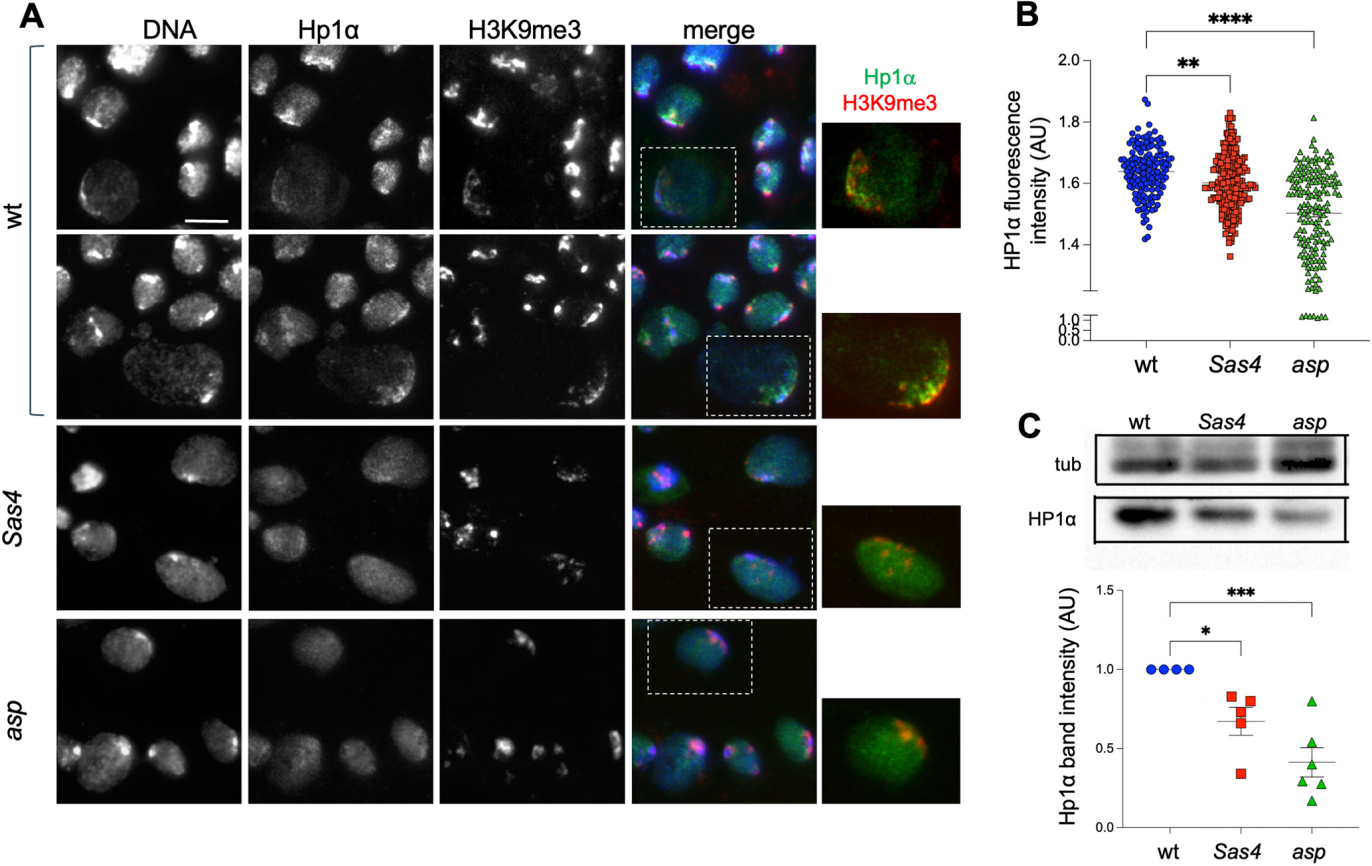
**HP1α is reduced and delocalized in the nucleus of *Sas4* and *asp* mutant cells.** (A) Immunostaining of wild-type and *Sas4* or *asp* mutant third instar larval brains stained with anti-HP1α (green) and H3K9me3 (red) antibodies and DAPI (blue, DNA). Dashed rectangles outline the brain cells shown on the right (1.5 times higher magnification). Scale bar: 10 μm. (B) Graphical representation of fluorescence intensity quantification of HP1α in brain cells of wild type (blue circles) and *Sas4* (red squares) or *asp* (green triangles) mutants. Each dot represents a single cell (*n*≥140). (C) Representative immunoblots for HP1α on protein extracts from wild type, *Sas4* or *asp* larval brains with the corresponding band quantification normalized to the loading control (tubulin) in at least three independent experiments. AU, arbitrary unit. Data are mean±s.e.m. *P<0.05; **P<0.01; ***P<0.001; ****P<0.0001 (B, ordinary one-way ANOVA test; C, Kruskal-Wallis ANOVA test).

Consistent with these results, we found that, in mutants, the reduction of H3K9me3 is accompanied by a corresponding decrease in total levels of heterochromatin protein 1α (HP1α), a specific component of heterochromatic regions (Fig. 4). HP1 binds to H3K9me2/3 and localizes to heterochromatin; its binding to methylated H3K9 plays an important role in the establishment and maintenance of 3D genome organization during development (Lachner et al., 2001; Smothers and Henikoff, 2001; Stutzman et al., 2024). HP1α is enriched in heterochromatic foci, which are readily visible in control brains stained with an HP1α antibody (Fig. 4A). Interestingly, there is no corresponding accumulation of HP1α at the residual H3K9me3 foci detected in *Sas4* and *asp* mutant brain cells (Fig. 4A). Quantification of HP1α fluorescence revealed reduced signal intensity in mutant larval brain cells compared to controls (Fig. 4B), confirmed by western blotting for HP1α (Fig. 4C).

**Fig. 5.**
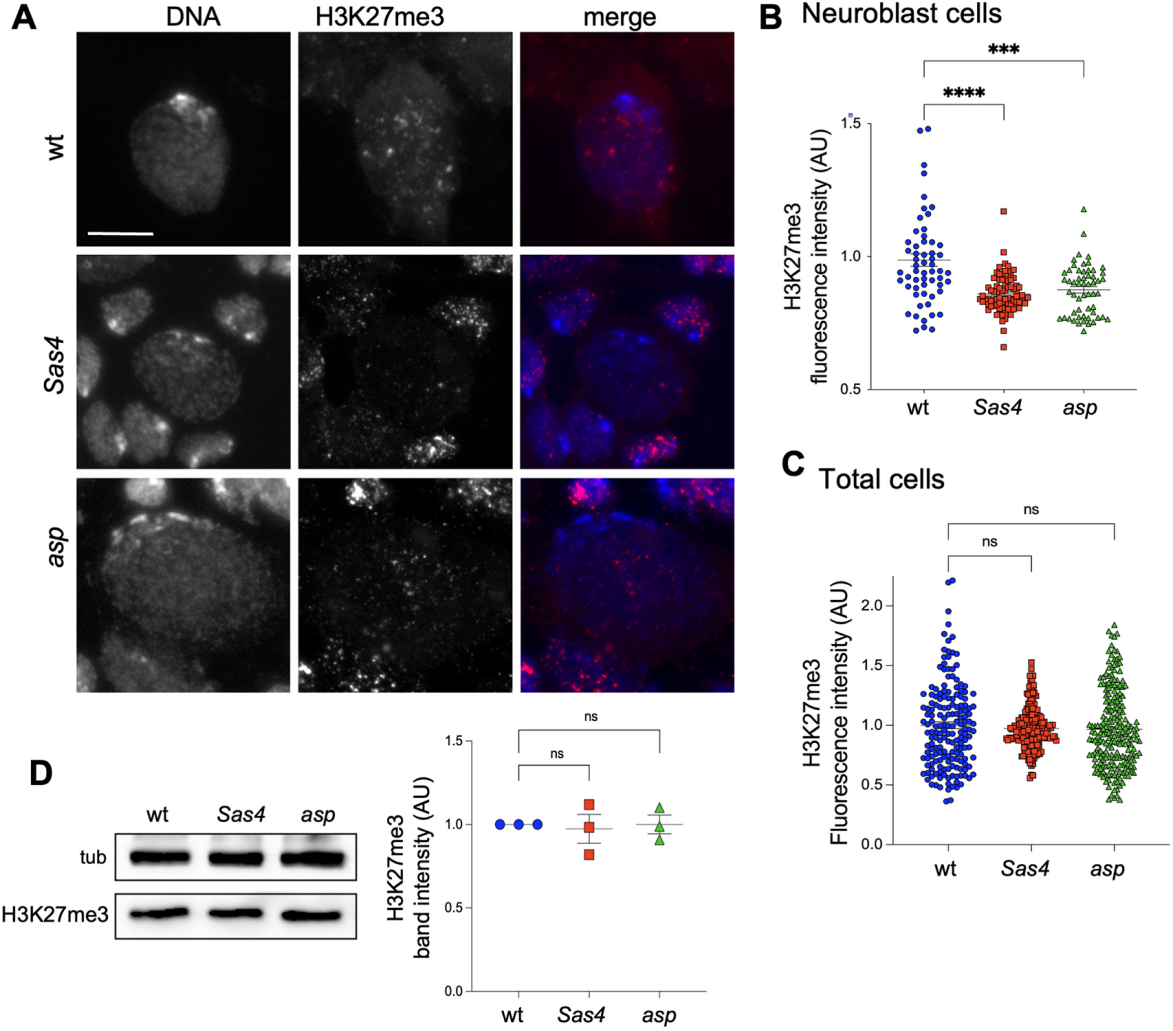
**The facultative heterochromatin mark H3K27me3 is reduced in *Sas4* and *asp* mutant neuroblasts.** (A) Immunolocalization of H3K27me3 (green) on third instar brains of wild type and *Sas4* or *asp* mutants. DNA (DAPI) is in blue. Scale bar: 10 μm. (B) Fluorescence intensity quantification of H3K27me3 in neuroblasts labelled as in A, showing a decrease of H3K27me3 in both *Sas4* (red squares) and *asp* (green triangles) mutant cells, compared to wild type (blue circles; *n*≥50). (C) Fluorescence intensity quantification of H3K27me3 in all larval brain cells/field; each dot represents a single cell (*n*≥150). (D) Representative immunoblots showing similar levels of H3K27me3 in larval brain extracts of *Sas4* and *asp* compared to wild type and the corresponding band quantification normalized to the loading control (tubulin) in at least three independent experiments. AU, arbitrary unit. Data are mean±s.e.m. ns, not significant; *P<0.05; **P<0.01; ***P<0.001; ****P<0.0001 (Kruskal-Wallis ANOVA test). Scale bar: 10 μm.

We next assessed the state of facultative heterochromatin, which is crucial for silencing lineage-specific genes during *Drosophila* larval brain development (Abdusselamoglu et al., 2019). To this end, we analysed H3K27me3 levels in *Sas4* and *asp* mutant larval brains. Immunostaining revealed reduced H3K27me3 signal intensity in mutant neuroblasts compared to wild-type cells (Fig. 5A,B). However, western blot analysis of whole-brain extracts showed no significant difference in overall H3K27me3 levels among wild-type, *asp* and *Sas4* mutants (Fig. 5D). This discrepancy likely reflects the fact that H3K27me3 levels remain high in differentiated cells in both mutant and control brains, while the reduction is specific to neuroblasts (Fig. 5A-C). Interestingly, analysis of the euchromatin mark H3K4me3 revealed an inverse pattern, with increased levels in mutant neuroblasts that were not detected by western blotting (Fig. S8).

Taken together, these data indicate that loss of either Sas4 or Asp reduces Lamin levels and alters Lamin nucleoskeleton architecture, leading to nuclear invaginations. This structural alteration may in turn contribute to the altered heterochromatin positioning and relaxation, indicating a functional role for Sas4 and Asp in the maintenance of nuclear architecture and heterochromatin during brain development.

### Polytene chromosome banding patterns are altered in *asp* and *Sas4* mutant salivary glands

**Fig. 6.**
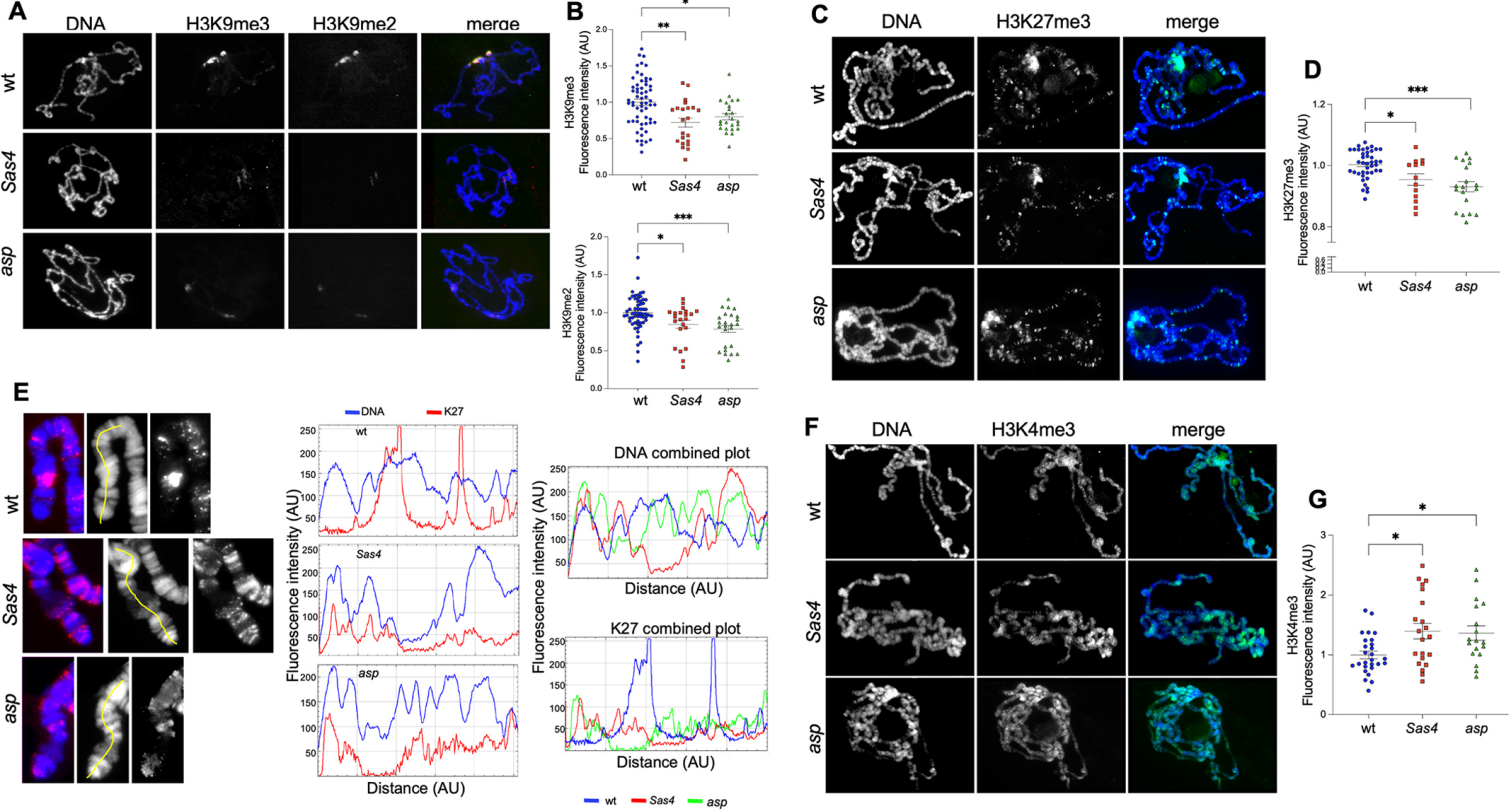
**H3K9me2/3 and H3K27me3 marks are reduced and altered in *Sas4* and *asp* mutant polytene chromosomes, while the euchromatin mark H3K4me3 is increased**. (A) Immunolocalization of H3K9me3 (red) and H3K9me2 (green) on wild-type and *Sas4* or *asp* mutant polytene chromosomes. DNA (DAPI) is in blue. (B) Fluorescence intensity quantification of H3K9me2 and H3K9me3 signals showing a significant decrease of both heterochromatic marks in mutants compared to wild type. Each dot represents a single polytene chromosome (wild type, *n*=60; *Sas4*, *n*=22; *asp*, *n*=25). (C) Immunolocalization of H3K27me3 (green) on wild-type and *Sas4* or *asp* mutant polytene chromosomes. DNA is in blue (DAPI). (D) Fluorescence intensity quantification of H3K27me3 signals showing a significant decrease of H3K27me3 in mutants compared to wild type. Each dot represents a single polytene chromosome (wild type, *n*=42; *Sas4*, *n*=14; *asp*, *n*=19). (E) Examples of the X chromosome extremities stained with anti-H3K27me3 (red) and DNA (blue, DAPI) in wild-type and *Sas4* or *asp* mutant polytene chromosomes with corresponding intensity profiles of DAPI (blue) and H3K27me3 (red) for each genotype. The DNA and H3K27me3 combined plots highlight the altered patterns in both *Sas4* (red line) and *asp* (green line) mutants compared to wild type (blue line); *y*-axis, fluorescence intensity; *x*-axis, distance from the tip of the chromosome. (F) Immunolocalization of H3K4me3 (green) on wild-type and *Sas4* or *asp* mutant polytene chromosomes. DNA is in blue (DAPI). (G) Graphical data quantification of fluorescence intensity of H3K4me3, as in F. Each dot represents a single polytene chromosome (wild type, *n*=27; *Sas4*, *n*=21; *asp*, *n*=18). AU, arbitrary unit. Data are mean±s.e.m. *P<0.05; **P<0.01; ***P<0.001 (ordinary one-way ANOVA test). All data were obtained from at least three independent experiments.

Polytene chromosomes from *Drosophila* salivary glands are characterized by a chromocenter that also contains under-replicated heterochromatin and bands of intercalary heterochromatin in euchromatic arms. This characteristic offers a unique model for studying the distribution and arrangement of chromatin marks on interphase chromosomes (Kolesnikova, 2018; Plata et al., 2009). To explore the pattern and dynamics of chromatin marks in *asp* and *Sas4* mutants, we performed immunostaining of polytene chromosomes from squashed third-instar salivary glands. Fluorescence quantification analysis confirmed a near-complete loss of H3K9me3/me2 signals, especially in the chromocenter, which is normally a strong signal-rich region due to its heterochromatic nature. This loss suggests a substantial reorganization or decondensation of heterochromatin in the mutant context (Fig. 6A,B). A significant reduction in the repressive H3K27me3 mark was also observed (Fig. 6C,D), along with a mild but significant increase in H3K4me3 euchromatic mark (Fig. 6F,G). Interestingly, the band signal profiles of both DNA and H3K27me3 were altered in polytene chromosomes from *asp* and *Sas4* mutants, suggesting an extensive reorganization of intercalary heterochromatin and possibly also of euchromatin (Fig. 6E and Fig. S9).

These changes in the DNA banding pattern further support our hypothesis that Asp and Sas4 loss modify chromatin organization, potentially altering gene expression programs during brain development. Therefore, given the well-established correspondence between chromatin organization in polytene chromosomes and other *Drosophila* tissues (Demakov et al., 2011; Eagen et al., 2015; Vatolina et al., 2011), the alterations observed in salivary glands can be considered representative of similar changes occurring in the CNS.

### Sas4 and Asp are required for genome integrity maintenance and DNA damage response

**Fig. 7.**
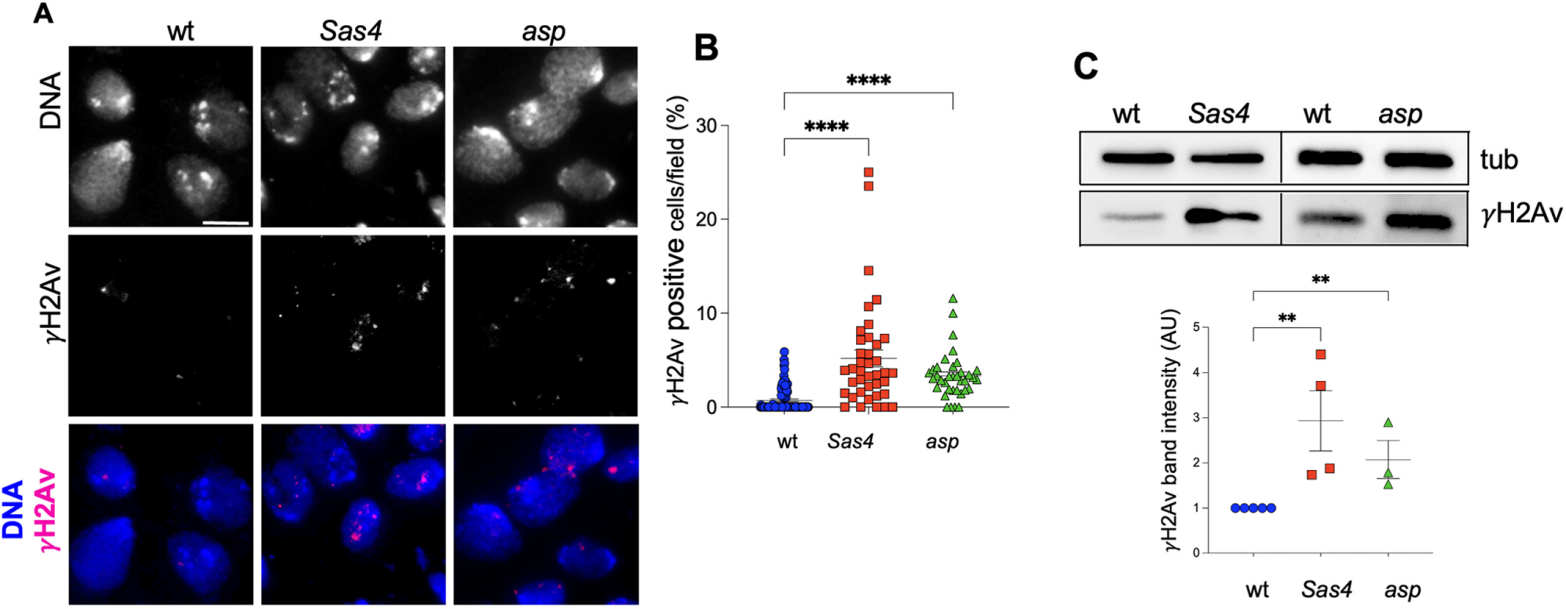
**Loss of Sas4 and Asp causes increased levels of γH2Av.** (A) Immunostaining of wild-type and *Sas4* or *asp* mutant third instar brain cells stained with anti-γH2Av (red) antibody and DAPI (blue, DNA). Scale bar: 10 μm. (B) Graphical representation of the percentage of γH2Av-positive cells per field, as shown in A. Wild type, *n*=99; *Sas4*, *n*=39; *asp*, *n*=36 for at least three brains. (C) Representative immunoblots on brain extracts from wild type and *Sas4* or *asp* mutant labelled using anti-γH2Av antibody with the corresponding band quantification normalized to the loading control (Tubulin). Data are mean±s.e.m. **P<0.01; ****P<0.0001 (Kruskal-Wallis ANOVA test).

One of the pathological hallmarks of microcephaly is genome instability caused mainly by persistent double-strand breaks (DBS) during development (Dileep et al., 2023; Provasek et al., 2022). Previous work has shown that the human orthologues of *Sas4* and *asp* are not only required for proper spindle assembly and function but also to prevent DNA damage (Wu et al., 2022; Xu et al., 2021). In addition, in *Drosophila* Sas4 loss is associated with increased DNA damage (Poulton et al., 2014). Thus, we analysed the levels of phosphorylated H2Av (γH2Av), a well-known marker of DNA damage, both by immunostaining and western blotting. Cytological analysis of γH2Av-positive cells revealed a significant increase in mutant larval brains compared to wild type (Fig. 7A,B), in line with the elevated γH2Av levels observed by western blot (Fig. 7C). These findings indicate that mutations in *Sas4* and *asp* lead to increased endogenous DNA damage, highlighting their crucial role in maintaining genome stability during *Drosophila* brain development.

**Fig. 8.**
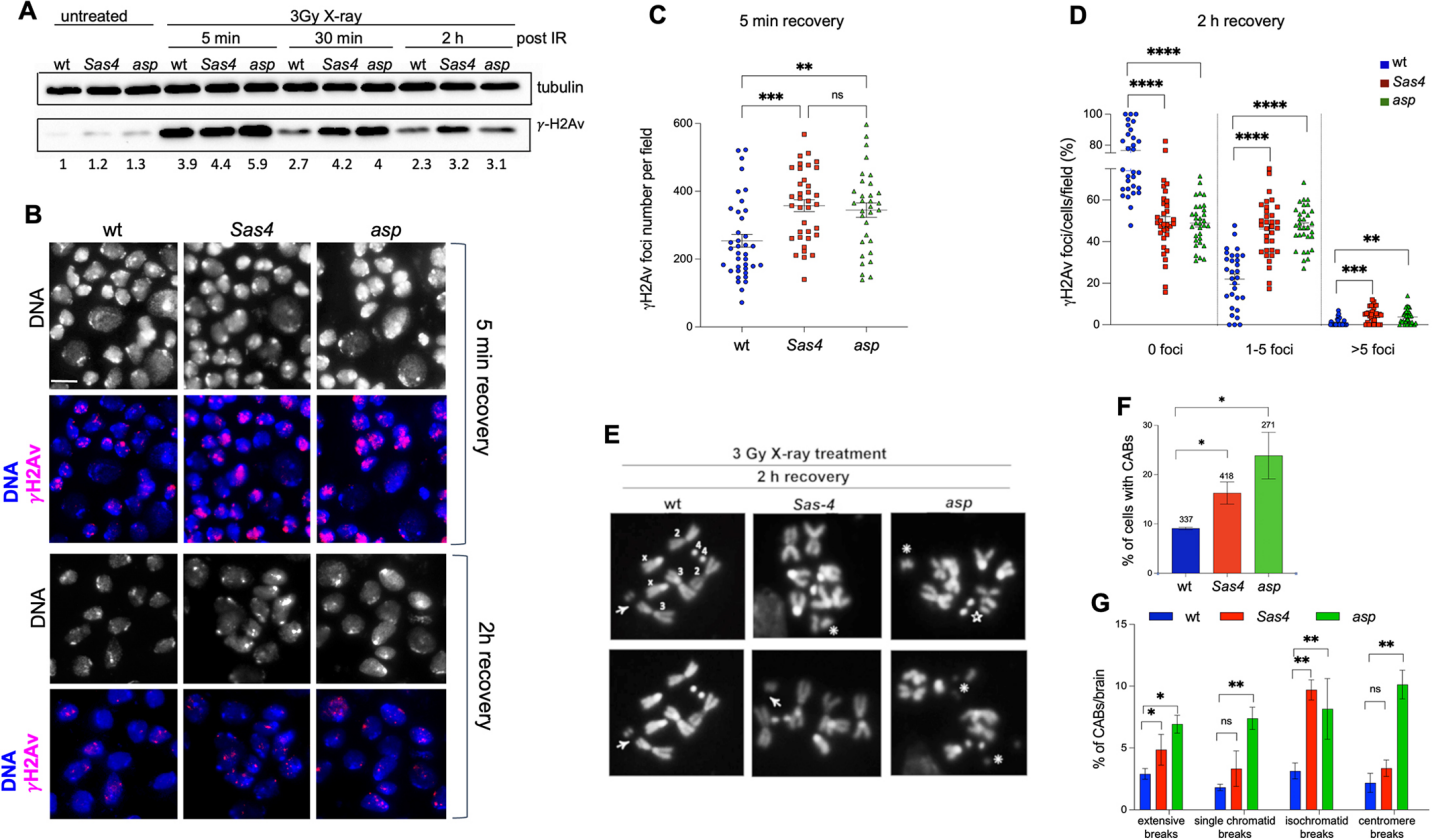
**Loss of *Sas4* or *asp* induces delayed DNA damage recovery and enhanced radiosensitivity.** (A) γH2Av levels in wild-type, *Sas4* and *asp* larval brain extracts after irradiation with 3 Gy and dissection after 5 min, 30 min and 2 h post irradiation (PI). Numbers below indicate the corresponding band quantification normalized to the loading control (Tubulin). (B) *Drosophila* brains from wild-type, *Sas4* and *asp* larvae, irradiated with 3 Gy and stained with anti-γH2Av antibody at 5 min or 2 h PI. Scale bar: 10 μm. (C) Quantification of the γH2Av foci per field (*n*≥30, from at least three brains) at 5 min PI time, as shown in B. (D) Graphical representation of the percentage of γH2Av-positive cells per field (*n*≥30) with 0, 1-5 or >5 foci per cell, in brain cells of wild type (blue circles), *Sas4* (red square) and *asp* (green triangles) mutants of at least three brains at 2 h PI time, as in B. (E) Examples of larval brains metaphases from wild type (numbers indicate autosomes and X symbols indicate sexual chromosomes) and *Sas4* or *asp* mutants at 2 h PI. Iso-chromatid breaks (arrows), extensive DNA fragments (asterisks) and chromatid deletions (star) are present in mutant cells. (F) Graphical representation of chromosome aberration (CAB) frequencies after 3 Gy treatment in wild-type, and *Sas4* or *asp* mutant brain cells. Numbers above the columns represent the number of total cells analysed in at least three larval brains. (G) Graphical representation of the percentage of different types of CABs after 3Gy treatment observed in wild-type, *Sas4* or *asp* mutant larval brain metaphase spreads. AU, arbitrary unit. Data are mean±s.e.m. *P<0.05; **P<0.01; ***P<0.001; ****P<0.0001 (ordinary one-way ANOVA test).

To understand if *asp* and *Sas4* loss also causes hypersensitivity to induced DNA damage, larval brains were exposed to 3 or 10 Gy (Ferri et al., 2026), dissected at different times, from 5 min to 2 h post-irradiation (PI), and then analysed to determine the kinetics of DNA damage repair. Western blot analysis showed a significant increase in γH2Av level compared to the wild type at all recovery times. In particular, at 2 h PI with 3 Gy, the level of γH2Av was significantly decreased in wild type while it remained high in the mutants (Fig. 8A). Similar results were obtained by using 10 Gy or after 2 h hydroxyurea (HU, 2 mM) treatment (Fig. S10). Consistently, immunofluorescence analysis revealed a significant increase in the number of γH2Av foci in the mutant cells compared to wild type, both at 5 min and 2 h PI (Fig. 8B-D). These results indicate that the DSB repair ability in the *Sas4* and *asp* mutant cells was impaired and significantly delayed, resulting in hypersensitivity to X-ray induced DNA damage. Because DSBs generate both γH2Av foci and chromosomal aberrations (CABs) (Merigliano et al., 2017), we examined CAB frequency in *Sas4* and *asp* larval brains. Third instar larvae were irradiated with 3 Gy and then colchicine-treated metaphase analysis was performed at 2 h PI. As expected, in Sas4- or Asp-depleted brain cells, we found a significant increase in metaphase cells with CABs and extensive chromosome fragmentation compared to wild type (Fig. 8E-G). Taken together, these data demonstrate that loss of either Sas4 or Asp causes hypersensitivity to DNA damage and a failure to resolve DSBs in larval brains, suggesting that these proteins are involved in the DNA damage response and play a key role in maintaining genome integrity.

### Broader impact of centrosome dysfunction on heterochromatin and genome integrity maintenance

An important aspect emerging from our study concerns whether the observed phenotypes are specific to *asp* and *Sas4* mutations or represent a broader consequence of centrosome or microtubule disorganization. To explore this possibility, we analysed homozygous mutants for another centrosomal gene, *spindle defective 2* (*DSpd-2^z3-3316^*), the *Drosophila* ortholog of human *CEP192*, which encodes a key protein required for pericentriolar material recruitment and MT nucleation from centrosomes (Conduit et al., 2014; Giansanti et al., 2008; Meghini et al., 2016). Consistent with its well-established roles in centrosome assembly and MT organization in neuroblasts, we found that *Spd2* mutants display abnormalities reminiscent of those observed in *asp* and *Sas4* mutants. Specifically, Spd2 loss results in reduced levels of heterochromatin marks H3K9me2-3 and H3K927me3 both in larval brain cells and polytene chromosomes, accompanied by increased phosphorylation of the histone variant H2Av, indicative of elevated DNA damage (Fig. S11). These findings suggest that disturbance of centrosome integrity or MT organization can broadly affect chromatin state and nuclear architecture.

## DISCUSSION

### The role of Sas4 and Asp in maintaining nuclear architecture and chromatin organization

In this study, we investigated the role of Sas4 and Asp, orthologs of human CENPJ and ASPM, in molecular pathways underlying MCPH pathogenesis. A hallmark of MCPH is a reduction in neuron number during development, often caused by imbalances in the tightly regulated processes of cell proliferation, differentiation and apoptosis. Consistently, *Drosophila Sas4* and *asp* mutant brains are significantly smaller, reflecting the reduced brain size characteristic of human microcephaly and highlighting the relevance of these models for studying MCPH mechanisms (Fig. S12; Mannino et al., 2023; Rujano et al., 2013).

Within this context, we show that Sas4 and Asp function not only in classical mitotic roles, such as centrosome function and spindle orientation, but also contribute to the organization of the interphase cytoskeleton, as shown by the altered MT architecture observed in *Sas4* and *asp* mutant brain cells. Notably, despite the distinct defects in microtubule organization observed in *Sas4* and *asp* mutant NBs, both conditions lead to similar nuclear invaginations, suggesting that different perturbations of the microtubule network can converge on comparable alterations in nuclear shape.

The cytoskeleton acts as a structural integrator of nuclear shape and positioning. Disturbance of its organization leads to deformation of the nuclear lamina, affecting chromatin organization and genome stability. Studies in *Drosophila* embryos have shown that MT polymerization produces pushing forces that affect nuclear envelope dynamics and promote chromatin mobility (Hampoelz et al., 2011). Notably, altered nuclear architecture, characterized by nuclear envelope invaginations, reduced Lamin levels and mislocalization of envelope proteins, has been linked to both neurodevelopmental and neurodegenerative disorders. In particular, studies in *Drosophila* tauopathy models have shown that Lamin dysfunction can drive chromatin decompaction and DNA damage (Frost et al., 2014, 2016; Paonessa et al., 2019; Park et al., 2022). Consistent with this, we found that Sas4 or Asp loss leads to reduced Lamin, relaxed heterochromatin and frequent nuclear envelope invaginations. These features reflect broad nuclear architectural defects. Moreover, reduced centromere clustering and altered abundance and localization of HP1α suggest that Sas4 and Asp are essential for proper heterochromatin compartmentalization. The interaction between H3K9 methylation and HP1α is crucial for organizing 3D genome architecture; its disturbance destabilizes pericentric heterochromatin. Studies in murine embryonic stem cells have shown that HP1 loss compromises self-renewal and pluripotency, partly through reduced H3K9 methylation (Dong et al., 2023).

In line with these findings, we observed decreased levels of H3K9me2/3 and H3K27me3 in *Sas4* and *asp* mutant brains, alongside increased H3K4me3, a mark of active chromatin, suggesting a shift toward transcriptional activation. Interestingly, total H3K27me3 levels were unchanged in whole-brain extracts, indicating that the mark was reduced in neuroblasts but remained stable in differentiated cells, pointing to differential regulation of repressive chromatin during development.

Further support comes from polytene chromosome analysis, where we observed altered banding patterns and reduced intensity of H3K9me2/3 and H3K27me3. These changes could influence gene expression programs relevant to particular developmental stages and cell-type specification (Abdusselamoglu et al., 2019; Marshall and Brand, 2017). Notably, H3K9 methylation plays a key role in maintaining cell identity by preventing inappropriate gene reprogramming and supports neuronal plasticity by silencing non-lineage genes during development (Nicetto and Zaret, 2019; Padeken et al., 2022). Altogether, our data suggest that loss of Sas4 or Asp perturbs heterochromatin organization and 3D genome architecture, potentially contributing to impaired gene regulation, defective cell cycle progression and, ultimately, developmental abnormalities.

Indeed, methylstat, which globally increases histone methylation by inhibiting demethylases, rescues nuclear invaginations caused by Asp or Sas4 loss, consistent with the role of overall chromatin state in nuclear architecture (Stephens et al., 2018; Xia et al., 2025). In contrast, Su(var)3-9 overexpression, which elevates H3K9 methylation in only a non-physiological manner, and thus does not restore the complete proper chromatin balance, is not sufficient to prevent nuclear defects. This suggests that the enhanced nuclear stiffness resulting from the global increase in methylation induced by demethylase inhibition helps to re-establish normal nuclear architecture by maintaining heterochromatin organization in developing brain cells.

Importantly, recent work has shown that impaired nuclear mechanics can hinder differentiation programs (Basso et al., 2025). In this context, the altered actomyosin network and nuclear deformation observed in *asp* and *Sas4* mutants may provide an additional mechanism through which MT dysfunction affects chromatin structure, gene expression and, potentially, neuroblast differentiation in MCPH.

### The role of Sas4 and Asp in DNA/chromosome stability and integrity

Several MCPH genes encode proteins involved in the DNA damage response (Iegiani et al., 2023). For instance, MCPH1 promotes homologous recombination, ASPM stabilizes BRCA1 for double-strand break repair, KNL1 deletion and CITK deficiency trigger DNA damage and apoptosis, and CENPJ mutations increase DNA damage in mice (Bianchi et al., 2017; Liu et al., 2016; McIntyre et al., 2012; Shi et al., 2019; Xu et al., 2021).

We found that loss of Sas4 or Asp leads to a significant increase in γH2Av foci and delayed recovery from DNA damage following exposure to genotoxic stress. Several DNA damage response (DDR) proteins, including ATR, ATM, 53BP1 and DNA-PKs, are known to localize to centrosomes, and microtubules are thought to act as tracks for transporting these proteins to the nucleus (Li et al., 2022; Maizels and Gerlitz, 2015). Furthermore, at least for ASPM, a direct interaction with known factors involved in DNA damage repair has been demonstrated (Feng et al., 2021; Wu et al., 2022; Xu et al., 2021). Our data suggest that Sas4 and Asp may also contribute to genome integrity through a distinct function. Given the roles of Sas4 and Asp in centrosome biogenesis and MT organization, it is plausible that they contribute to DDR regulation through these cytoskeletal functions. In addition, recent studies have revealed that cytoplasmic MTs, in concert with motor proteins, facilitate the movement of DNA damage sites within the nucleus, thereby promoting repair by relocating lesions to specialized, repair-conducive nuclear domains (Lawrimore et al., 2017; Lottersberger et al., 2015; Shokrollahi et al., 2024). In this context, Sas4 and Asp may support these processes by maintaining MT organization and enabling damage site mobility. Another key factor influencing DNA repair efficiency is the local chromatin environment. While chromatin relaxation is often required to allow repair machinery access to DNA lesions, decondensed chromatin is more susceptible to DNA damage compared to compacted heterochromatin (Chiolo et al., 2025; Clouaire et al., 2018; dos Santos et al., 2020; Ortega et al., 2021). Thus, disturbances of chromatin organization not only impair repair but may also increase the accumulation of DNA damage. Importantly, defects in nuclear architecture, including abnormal nuclear morphology, increased DNA damage and altered chromatin compaction, are common features of various pathologies, such as premature aging syndromes and cancers, many of which involve mutations in nuclear envelope components (Kalukula et al., 2022).

Taken together, these observations support the hypothesis that the impaired DNA damage recovery observed in *Sas4* and *asp* mutant cells may result from nuclear envelope defects and heterochromatin loss, linking centrosome and cytoskeletal defects to compromised genome integrity. Remarkably, our findings emphasize the potential role of the microtubule cytoskeleton in the epigenetic dysregulation that underlies the aetiology of microcephaly. While our study specifically focuses on Asp and Sas4 as key regulators of these processes, the similar phenotypes observed in *Spd2* mutants support the idea that maintaining centrosome integrity is broadly required to preserve nuclear organization and chromatin homeostasis. These results are consistent with recent evidence linking biallelic CEP192 variants to microcephaly, probably through centrosome dysfunction causing defective mitosis and reduced neural progenitor growth (Guo et al., 2024). Together, these findings point to a previously unreported potential role for centrosome proteins, such as Asp, Sas4 and Spd2, in coordinating chromatin organization, ensuring proper neuroblast development and providing new insight into MCPH pathogenesis.

### Conclusions

Emerging evidence highlights the crucial role of a proper coordination between centrosomes, microtubule/actin cytoskeleton, nuclear envelope and heterochromatin in maintaining nuclear and chromatin dynamics, ultimately ensuring genome organization and stability (Shokrollahi and Mekhail, 2021; Uhler and Shivashankar, 2017; van Steensel and Belmont, 2017). Disturbance of any component within this axis can lead to aberrant gene expression by altering the spatial organization of chromosomes (Vahabikashi et al., 2022). Indeed, numerous diseases, including cancer and neurodegenerative disorders, are associated with defective microtubule networks, abnormal nuclear morphology and genomic instability.

This model may also extend to explain the molecular mechanisms underlying microcephaly caused by other *MCPH*-associated mutations, as we show for *Spd2*. Indeed, at least 20 of the 30 known *MCPH* genes encode factors that are essential for the assembly and function of key cellular structures, including centrosomes, microtubules, chromatin and the nuclear envelope, which all work together to preserve proper chromatin architecture.

### Limitations

While our study identifies previously unreported roles for Asp and Sas4 in maintaining nuclear architecture, chromatin integrity and genome stability, some limitations should be acknowledged. First, our analyses were performed primarily in *Drosophila* neuroblasts, which, although genetically tractable and evolutionarily conserved, may not fully recapitulate the complexity of mammalian neurodevelopment. Validation in mammalian models or patient-derived cells would strengthen the translational relevance of our findings. Second, although treatment with the demethylase inhibitor methylstat restored heterochromatin marks and nuclear morphology, we did not dissect the underlying molecular mechanism or evaluate long-term functional rescue.

## MATERIALS AND METHODS

### *Drosophila* strains and rearing conditions

*Drosophila* stocks were long-term maintained at 18°C, while the stocks used in the experiments were maintained and bred at 25°C. For RNAi, we used *tubulin-GAL4* to induce expression of the UAS-RNAi construct and the fly crosses were incubated at 29°C to increase the efficiency of RNAi. Fly stocks used in this study are listed in Table 1.

**Table 1.** *D. melanogaster* genetic resources

Stock designation	Source or reference	Identifiers	Additional information
*tubulin-GAL4*	Bloomington	5138	y[1] w[*]; P{w[+mC]=tubP-GAL4}LL7/TM3, Sb[1] Ser[1]
*Sas4^s2214^*	Bloomington	12119	Shokrollahi and Mekhail (2021); Uhler and Shivashankar (2017)
*asp^1^*	Bloomington	1972	*In(3R)Ubx[7LL]ats[R], asp[1] ats[1] p[p]/TM6B, Tb[1] ca[1]; y[1]/Dp(1;Y)y[+]*
*asp^t25^*	Schoborg et al. (2015)	-	-
*DSpd-2^z3-5711^*	Giansanti et al. (2008)	-	-
*UAS asp^RNAi^*	Bloomington	28741	*y[1] v[1]; P{y[+t7.7] v[+t1.8]=TRiP.JF03169}attP2*
*UAS Sas4^RNAi^*	Bloomington	35049	y[1] sc[*] v[1] sev[21]; P{y[+t7.7]v[+t1.8]=TRiP.HMS01463}attP2
*W^1118^*	Bloomington	-	-
*UAS-Su(var)3-9^OE^*	Bloomington	93147	y[1] w[*]; P{w[+mC]=UAS-Su(var)3-9.lacI}2

### X-ray and HU treatment

X-ray treatment was carried out using MHF PLUS (Portable X-ray unit-Gilardoni). Third instar *Drosophila* larvae were irradiated with 3 and 10 Gy, and dissected after 5 min, 30 min, 2 h and 6 h (Ferri et al., 2026). Hydroxyurea (HU) treatment was performed by incubating larval brains for 30 min in 2 mM HU and then in tissue culture medium for 2 h. Brains were then collected for subsequent analyses.

### Methylstat treatment

The third instar larvae were dissected, and the brains were collected and treated with 3 μM methylstat in Schneider's medium for 4 h. Subsequently, after lysis, the brain protein extracts from each sample were subjected to western blot analysis. For immunohistochemistry, the brains were fixed with 3.7% formaldehyde followed by the steps in the ‘Immunohistochemistry’ section. All reagents are listed in Table S1.

### Immunohistochemistry

The samples were incubated in 3.7% formaldehyde for 20 min, transferred to 45% acetic acid for 30 s and fixed in 60% acetic acid for 2 min. Successively, the brains were squashed and frozen in liquid nitrogen. After the removal of the coverslips, the slides were placed in ice-cold ethanol for 15 min, rinsed twice for 10 min in 0.1%TritonX-100/PBS (PBT), and incubated overnight at 4°C with the primary antibodies listed in Table S1. After two washes in PBT for 10 min each, the slides were incubated for 1 h at room temperature with the appropriate secondary antibodies listed in Table S1. Immunostained preparations were mounted in Vectashield H-200 with DAPI (Vector Laboratories). Cytological preparations were examined using either fluorescence microscope Nikon equipped with a Charged-Coupled Device (CCD camera; Photometrics CoolSnap HQ) or confocal laser scanning microscopy Zeiss LSM980, using a 63×/1.40 NA oil objective and excitation spectral laser lines at 405, 488, 543 or 594, and 647 nm. Image acquisition and processing were carried out using the Zeiss confocal software Zen 3.3 (Blue edition) and Adobe Photoshop CS5 software programs (Adobe Systems).

### Polytene chromosome immunostaining

Salivary glands were dissected in physiological solution and fixed with 1.8% paraformaldehyde and 45% acetic acid for 7 min, then transferred onto a glass slide cover with a coverslip and then squashed with gentle tapping. The spread of polytene chromosome preparations was checked under microscope and then frozen in liquid nitrogen for 10 min. After removal of the coverslips, the slides were immersed in ice-cold TBS for 10 min, then rinsed twice for 10 min each in 0.1% Tween-20/TBS (TBS-T) and then incubated with the appropriate primary antibodies listed in Table S1. The preparations were washed twice in TBS-T for 10 min each and incubated with the appropriate secondary antibodies, listed in Table S1, for 1 h at room temperature. The preparations were then washed twice in TBS-T for 10 min each and mounted in Vectashield H-200 with DAPI (Vector Laboratories). All reagents are listed in Table S1.

### Chromosome cytology

To analyse metaphase chromosomes, brains were dissected in physiological solution (0.7% NaCl), incubated with colchicine (10^−5^ M in PBS) for 45 min, and then for 10 min in hypotonic solution (0.5% sodium citrate). The preparations were squashed in 45% acetic acid and frozen in liquid nitrogen for at least 10 min. The preparations were mounted in Vectashield H-200 with DAPI (Vector Laboratories). All reagents are listed in Table S1.

### Immunoblot and antibodies

Protein extracts from *Drosophila* larval brains were obtained by dissecting 10 larval brains in 0.7% NaCl, diluted in 20μl of 2×Laemmli buffer [4% SDS, 10% 2-mercaptoethanol, 20% glycerol, 0.004% Bromophenol Blue, 0.125 M Tris HCl (pH 6.8)], boiled for 5 min and centrifugated at 5000 ***g*** for 30 s. Protein samples were loaded onto 15% SDS-PAGE gels and blotted onto PVDF or nitrocellulose filter membrane (Hybond ECL, Amersham). Filters were blocked in 5% non-fat dry milk dissolved in 0.1% Tween-20/PBS for 30 min at room temperature and then incubated with the appropriate antibodies, listed in Table S1, overnight at 4°C. The blots were washed three times with 1×TBS-T for 5 min each, incubated with the appropriate HRP-conjugated secondary antibodies for 1 h at room temperature and then washed again three times with 0.1%Tween-20/PBS. The chemiluminescent signal was revealed through either SuperSignal West Femto or SuperSignal West Pico substrate (Thermo Scientific) using the ChemiDoc scanning system (Bio-Rad). All reagents are listed in Table S1.

### Quantification and statistical analysis

For analysis of the fluorescence intensity, the corrected total cell fluorescence (CTCF) was quantified using Image J software and calculated using the following formula: CTCF=Mean fluorescence intensity−(Area of selected nucleus×background mean fluorescence intensity).

Western blot band intensity measurements were quantified by densitometric analysis using the Image Lab 4.0.1 software (Bio-Rad). Western blotting was repeated independently at least three times.

Statistical analyses were performed using one-way ANOVA or the Kruskal–Wallis test for multiple groups, and an unpaired *t*-test or Mann–Whitney U-test for two-group comparisons, depending on data distribution using GraphPad Prism 8.1. When required, data were normalized with the transform Prism tool. All software and algorithms are listed in Table S1.

## Supplementary Material



10.1242/develop.205125_sup1 Supplementary information
