# Supplementary material for: Microcephaly-associated genes asp and Sas4 influence chromatin organization and nuclear lamina structure in Drosophila melanogaster
Source: Development. 2026 May 28;153(10):dev205125. doi: 10.1242/dev.205125 (PMC13286369; doi:10.1242/dev.205125)
Supplement: Supplementary information [file develop-153-205125-s1.pdf]

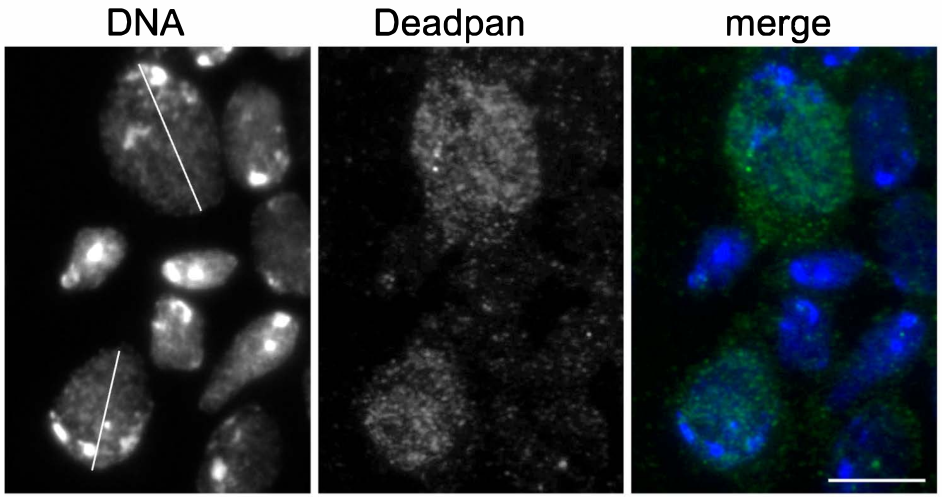

Fig. S1. Deadpan neuroblast labelling.
Wild type third instar brain squashes stained with the neuroblast specific marker Deadpan (green) and DAPI (blue). This allowed to measure the nuclear diameter (white line) of Deadpan-positive wild type neuroblasts, which validates the use of nuclear size (diameter >10 µm) as a reliable criterion for NB identification.

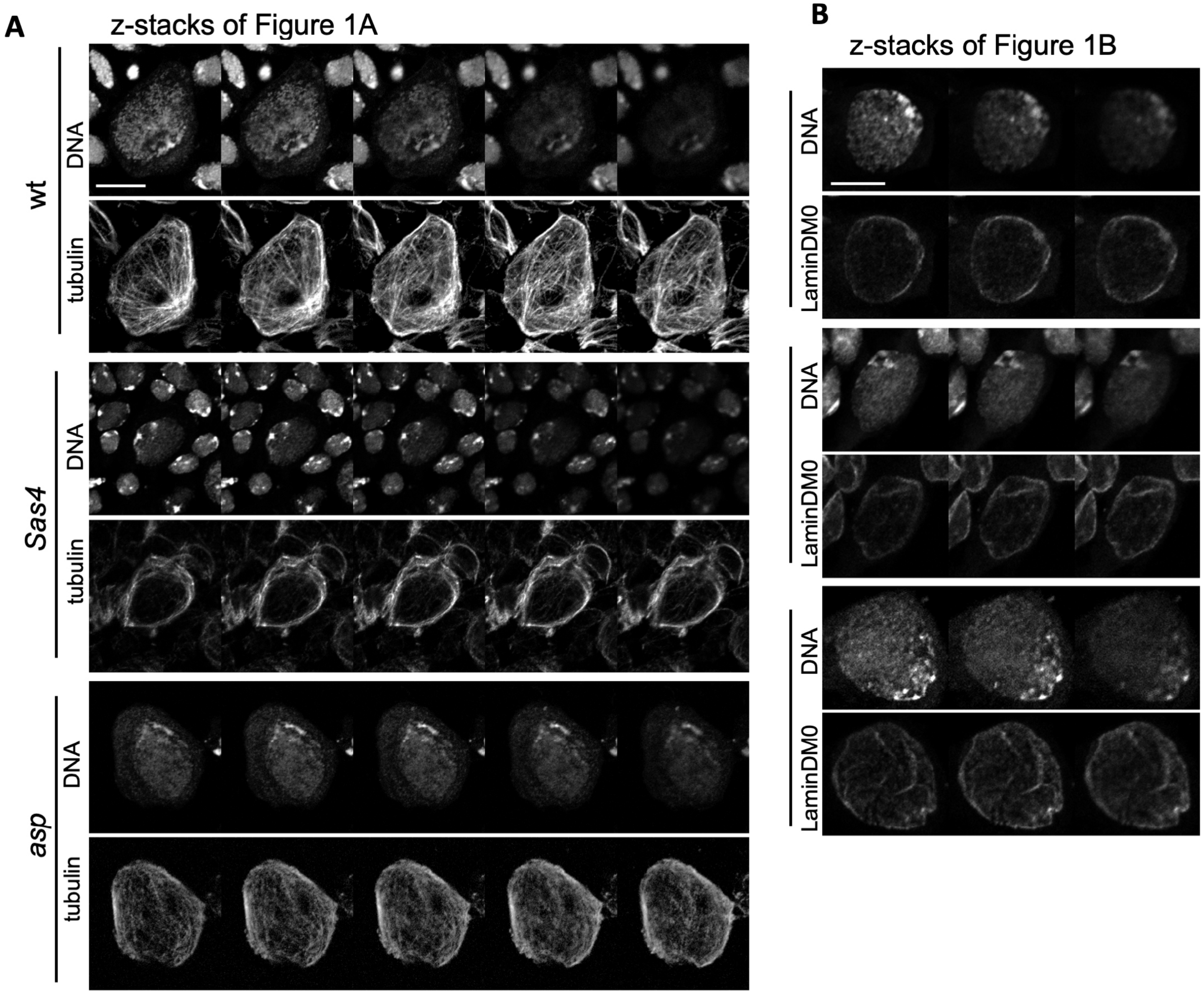

Fig. S2. Confocal Z-stack images. Z-stack images related to Figure 1A showing that in all sections, *Sas4* mutant cells display MT bundles restricted to the cell periphery, and *asp* mutants show a disorganized MT network composed of short, thin filaments. B) Z-stack images related to Figure 1B showing that in wt cells, the Lamin signal remains restricted to the nuclear periphery in all Z planes, whereas in mutant cells, Lamin forms inward extensions or streams within the nucleoplasm visible inall Z-stacks.

Development • Supplementary information

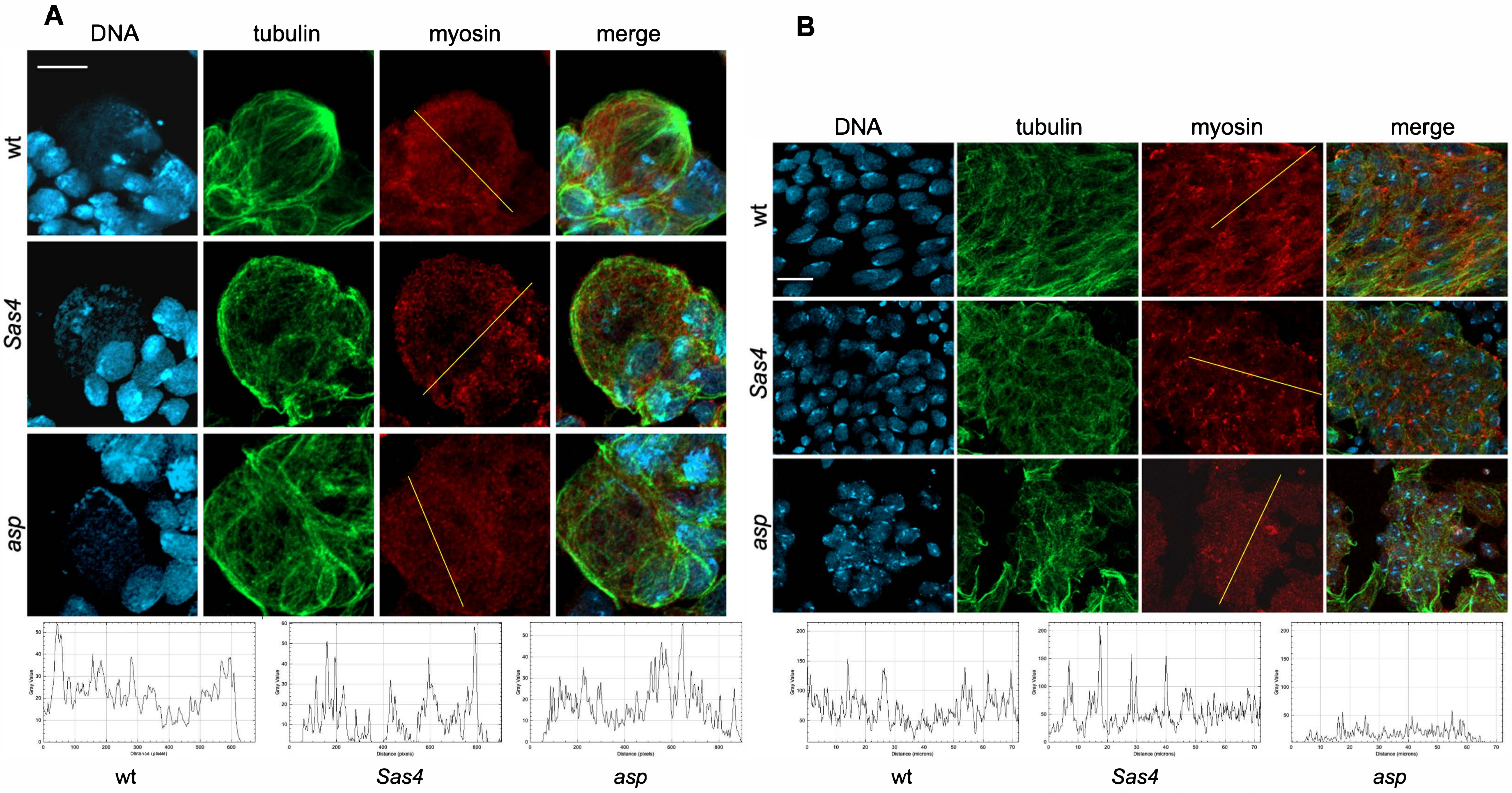

	Mean	StdDev
wt	673.394	245.787
Sas4	541.887	344.489
asp	152.825	109.001

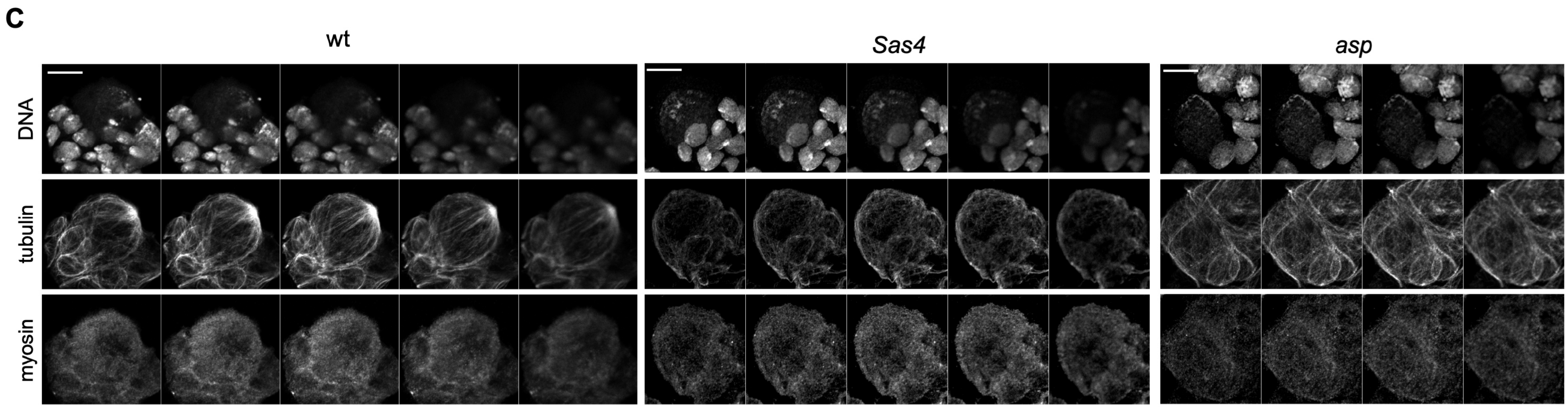

z-stacks of Figure S3A

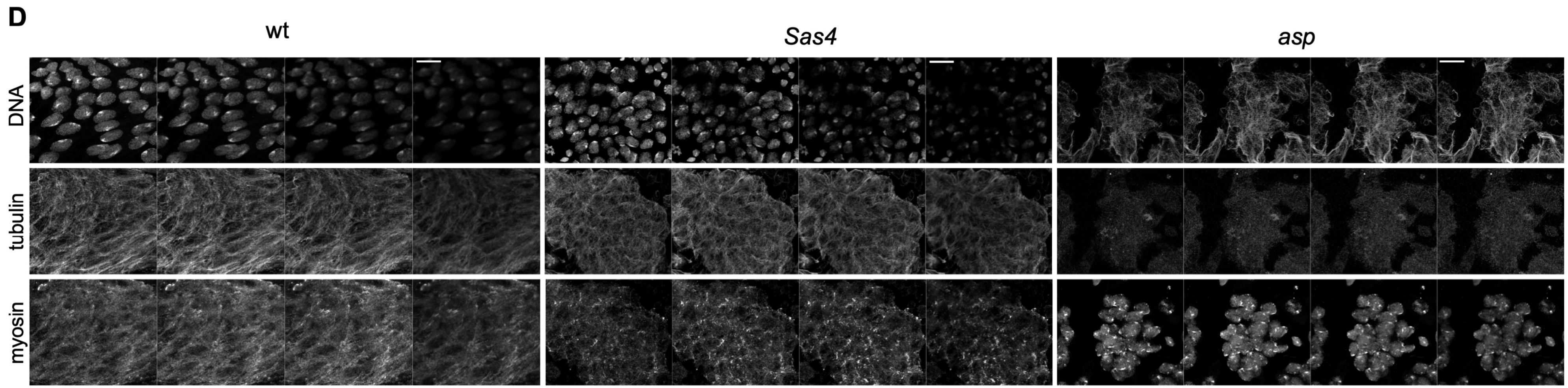

z-stacks of Figure S3B

Fig. S3. Loss of Sas4 or Asp leads to alterations of the actin-myosin network

Immunostaing of wt and Sas4 or asp larval brain cells using an antibody against the nonmuscle myosin 2 heavy chain protein (red), an antibody against the tubulin (green) and DAPI (blue, DNA), with the corresponding line-scan intensity profiles of myosin signal in brain cells of each genotype. A) Neuroblasts; B) Monolayer of brain cells. The table shows the mean intensity of the myosin signal± standard deviation, quantified from at least ten fields of monolayer cells. Notably, Sas4 cells displayed a very high standard deviation, indicating regions of higher and lower intensity suggestive of aggregation and uneven distribution. C) Z-stacks of figure A. D) Z-stacks of figure B. Scale bar = 10 μm. Images in A and B are maximum-intensity projections

Development • Supplementary information

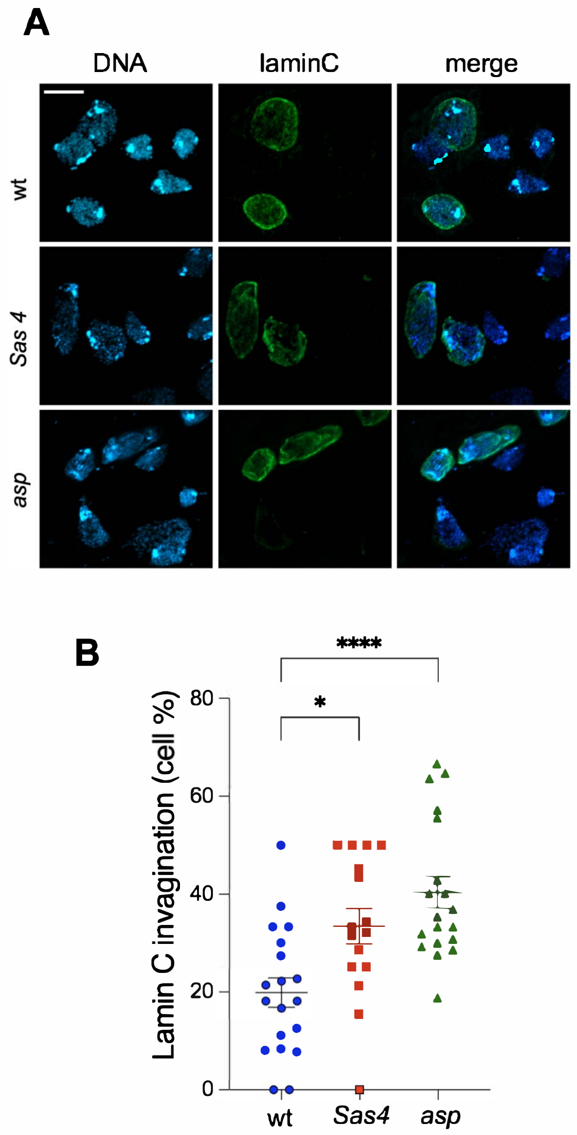

Fig. S4. Lamin C stains nuclear envelope invaginations in *Sas4* and *asp* mutant brain cells.

A) Cells from third instar brain squashes of wt and *Sas4* or *asp* mutants immunostained with anti-LaminC (green) antibody and DAPI (DNA, blue). B) Graphical representation of the percentage of cells showing invaginations of the NE in *Sas4* (red rectangles) and *asp* (green triangles) mutant and wt (blue circles) brain cells. Each dot represents the score of cells per 63x microscope field in 3 brains per genotype ($n \geq 16$). AU, arbitrary unit. Error bars represent SEM. P = p-value calculated using unpaired t test. *$p < 0.05$; **$p < 0.01$; ***$p < 0.001$; ****$p < 0.0001$. Scale bar = 10 µm. Images in A are maximum-intensity projections.

Development • Supplementary information

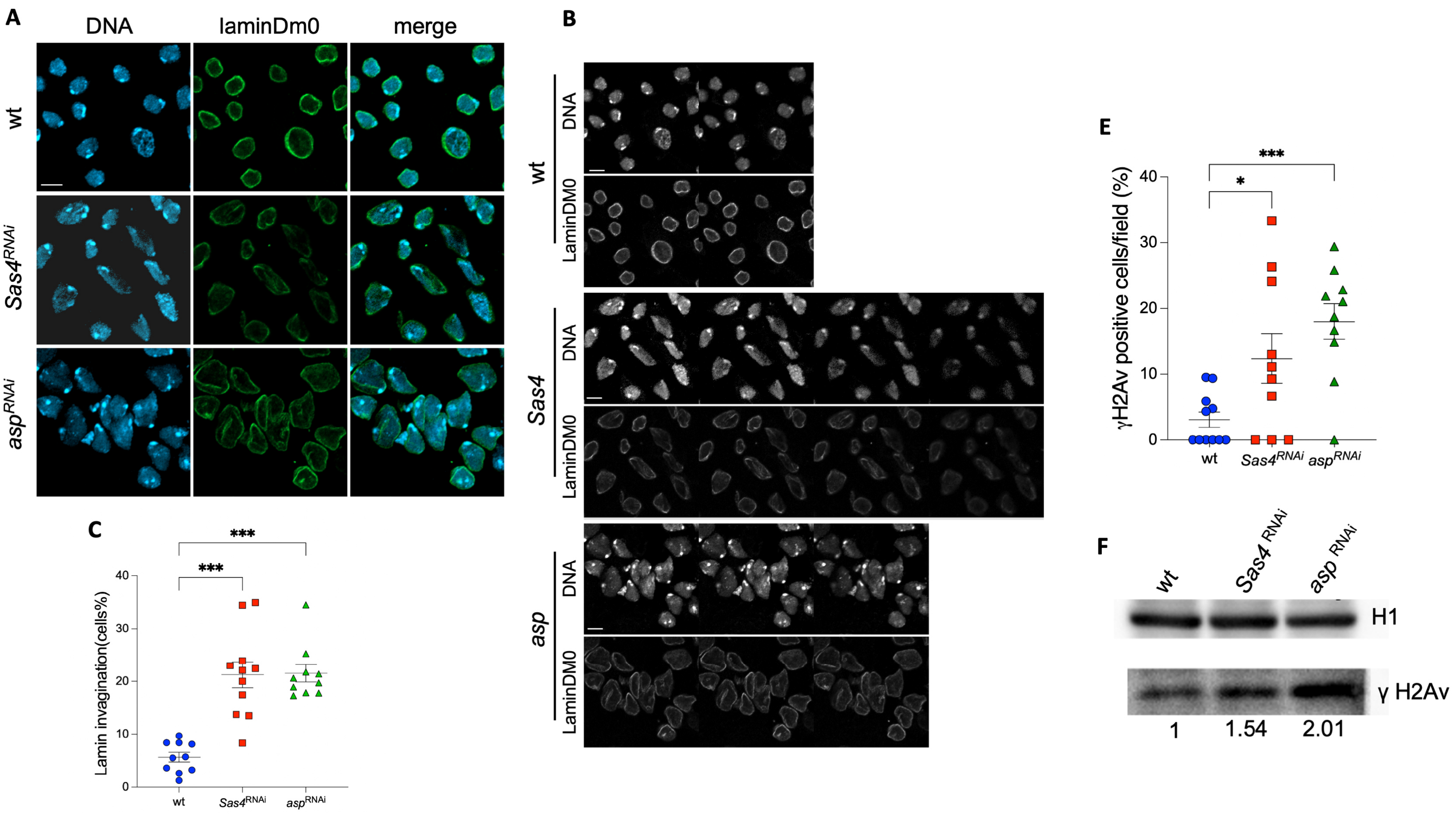

Development • Supplementary information

Fig. S5. RNAi-mediated silencing of *Sas4* or *asp* recapitulates both the lamin alteration and the increased levels of γH2Av of mutant phenotypes.

A) Third instar brain squashes of wt and *Tub-GAL4>UAS-Sas4^{RNAi}* or *UAS-asp^{RNAi}* immunostained with anti-LaminDm0 antibody (green) and DAPI (blue, DNA). B) Z-stack images related to Figure S5A, showing that in wild-type cells, the Lamin signal remains restricted to the nuclear periphery in all Z planes, whereas in silenced cells, Lamin forms inward extensions or streams within the nucleoplasm visible in all Z-stacks. C) Graphical representation of the percentage of cells showing NE in *Tub-GAL4>UAS-Sas4^{RNAi}* (red rectangles) and *UAS-asp^{RNAi}* (green triangles) and wt (blue circles) brain cells. Each dot represents the score of cells per 63x microscope field ($n \geq 10$). D) Graphical representation of the percentage of γH2Av positive cells per field, $n \geq 10$. F) Immunoblots on brain extracts from wt and *Tub-GAL4>UAS-Sas4^{RNAi}* or *UAS-asp^{RNAi}* labelled using anti-γH2Av antibody with the corresponding band quantification normalized on the loading control (H1). Error bars represent SEM. P = p-value calculated using unpaired t test. *$p < 0.05$; **$p < 0.01$; ***$p < 0.001$; ****$p < 0.0001$. All images are maximum-intensity projections. Scale bar = 10 µm.

Development • Supplementary information

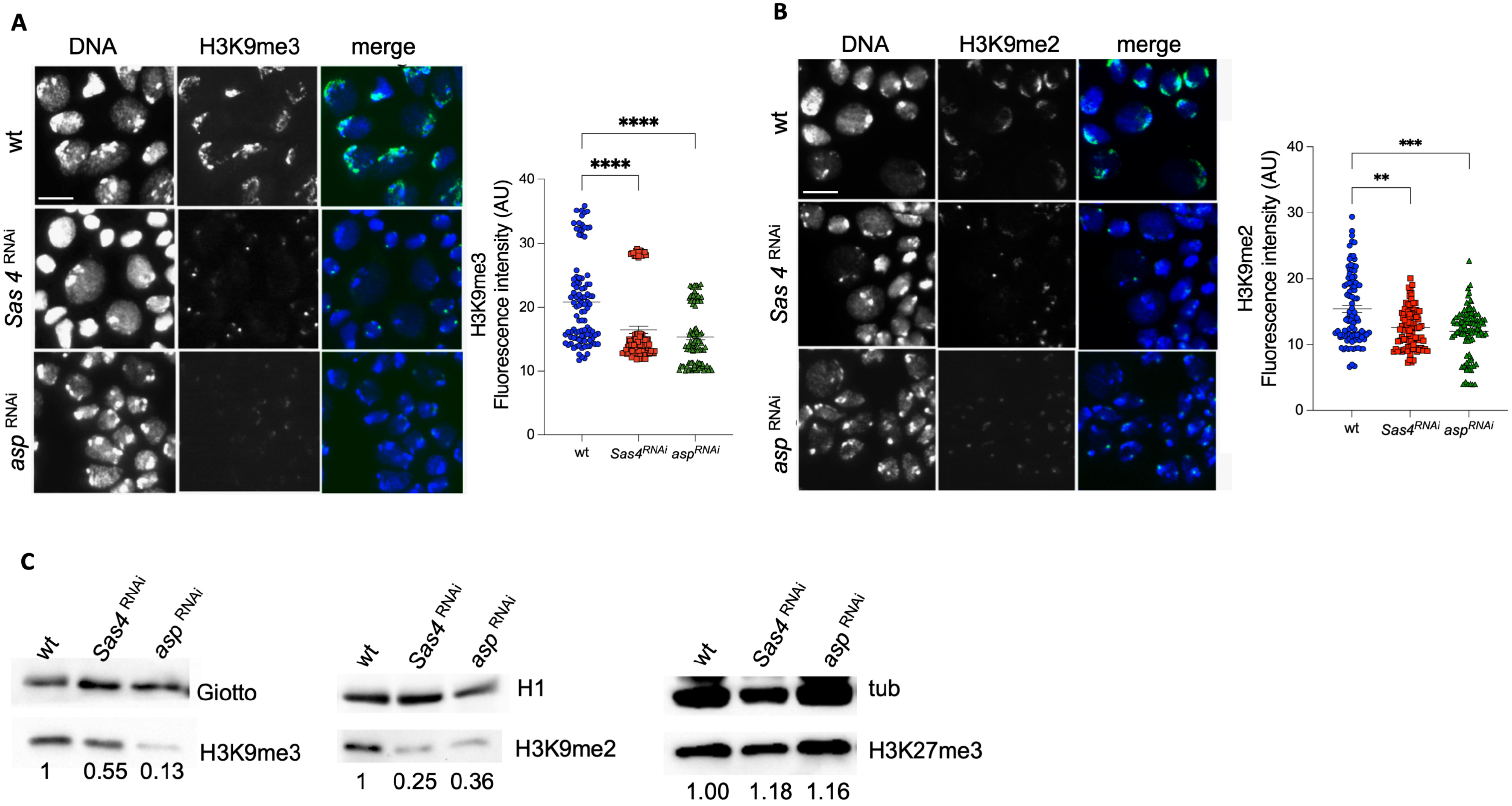
A
DNA
H3K9me3
merge
wt
Sas 4 RNAi
asp RNAi
H3K9me3 Fluorescence intensity (AU)
****
****
wt
Sas4RNAi
aspRNAi
B
DNA
H3K9me2
merge
wt
Sas 4 RNAi
asp RNAi
H3K9me2 Fluorescence intensity (AU)
***
**
wt
Sas4RNAi
aspRNAi
C
wt
Sas4 RNAi
asp RNAi
Giotto
H3K9me3
1 0.55 0.13
H1
H3K9me2
1 0.25 0.36
tub
H3K27me3
1.00 1.18 1.16

Fig. S6. RNAi-mediated silencing of *Sas4* or *asp* recapitulates the heterochromatin marks reduction of mutant phenotypes.

A) Third instar brain squashes of wt and *Tub-GAL4>UAS-Sas4^{RNAi}* or *UAS-asp^{RNAi}* immunostained with anti-H3K9me3 and quantification of H3K9me3 fluorescence intensity per cell as in (A), each dot represents a single cell from at least three brains ($n \geq 50$). B) Third instar brain squashes of wt and *Tub-GAL4>UAS-Sas4^{RNAi}* or *UAS-asp^{RNAi}* immunostained with anti-H3K9me2 and quantification of H3K9me2 fluorescence intensity per cell as in (B), each dot represents a single cell from at least three brains ($n \geq 50$). C) Immunoblots showing reduced levels of both H3K9me3 and me2 and no difference in H3K27me3 level upon *Sas4* or *asp* silencing compared to wt. Numbers below indicate the corresponding band quantification normalized on the loading control (Giotto, H1 or tubulin). Error bars represent SEM. P = p-value calculated using unpaired t test. *$p < 0.05$; **$p < 0.01$; ***$p < 0.001$; ****$p < 0.0001$. Scale bar = 10 µm.

Development • Supplementary information

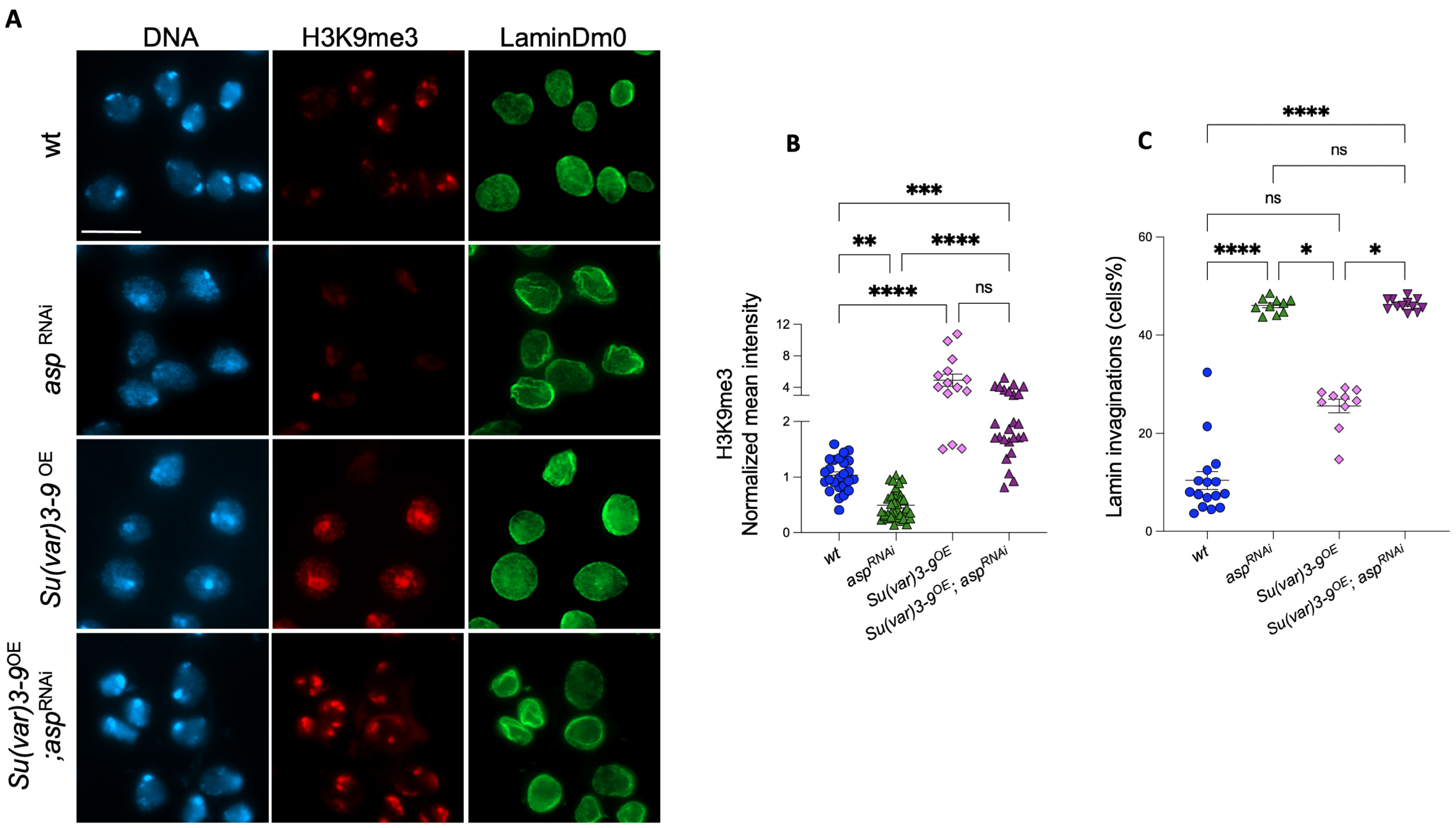
A
DNA
H3K9me3
LaminDm0
wt
asp RNAi
Su(var)3-9 OE
Su(var)3-9OE ;aspRNAi
B
H3K9me3 Normalized mean intensity
***
**
****
****
ns
12
8
4
2
1
0
wt
aspRNAi
Su(var)3-9OE
Su(var)3-9OE; aspRNAi
C
Lamin invaginations (cells%)
****
ns
ns
****
*
*
60
40
20
0
wt
aspRNAi
Su(var)3-9OE
Su(var)3-9OE; aspRNAi

Fig. S7. Genetic reconstitution of H3K9 methylation is not sufficient to impede nuclear invagination.

A) Third instar brain squashes of wt and *Tub-GAL4> UAS-asp*^{RNAi} larvae with or without Su(var)3-9 overexpression [*Su(var)3-9*^{OE}] imrnunostained with anti-H3K9me3 (red) and anti-Lamin Dm0 (green). Scale bar = 10 µm. B) Quantification of H3K9me3 fluorescence intensity per cell in wt (blue circles), *UAS-as*^{Ai} (green triangles), *Su(var)3-9*^{OE} (pink diamonds) and *UAS-as*^{RnAi} + *Su(var)3-9*^{OE} (purple inverted triangles) larvae. each dot represents a single cell from at least three brains ($n \geq 15$). C) Graphical representation of the percentage of brain cells showing invaginations of the NE as in B. Each dot represents the score of cells per 63x microscope field in at least 3 brains per genotype ($n \geq 10$). Error bars represent SEM. P = p-value calculated using unpaired t test. *$p < 0.05$; **$p < 0.01$; ***$p < 0.001$; ****$p < 0.0001$. All images are maximum-intensity projections.

Development • Supplementary information

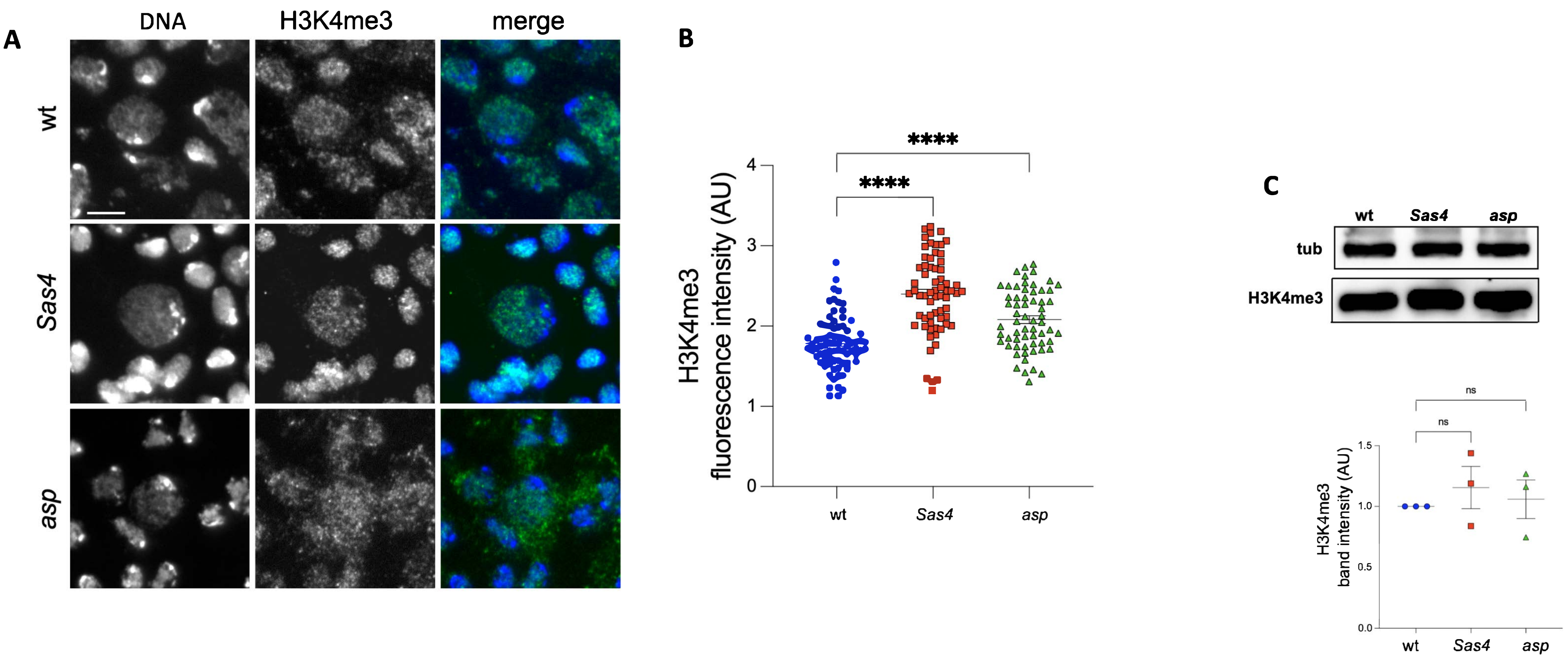

Fig. S8. Loss of Sas4 or Asp alters H3K4me3 levels in neuroblasts.

A) Immunolocalization of H3K4me3 on brain squashes from third instar larvae of wt and *Sas4* or *asp* mutants. In the merged panels, H3K4me3 is in green and DNA in blue (DAPI). Scale bar = 10 µm. B) H3K4me3 fluorescence intensity quantification in neuroblasts, showing an increase of H3K4me3 in both *Sas4* and *asp* mutants compared to wt, each dot represents a single cell (n ≥50). C) Representative western blots showing no change of H3K4me3 in larval brain extracts of *Sas4* or *asp* mutants in comparison to wt extracts with the corresponding band quantification normalized on the loading control (Tubulin). AU, arbitrary unit. Error bars represent SEM. P = p-value calculated using unpaired t test. *p < 0.05; **p < 0.01; ***p < 0.001; ****p < 0.0001.

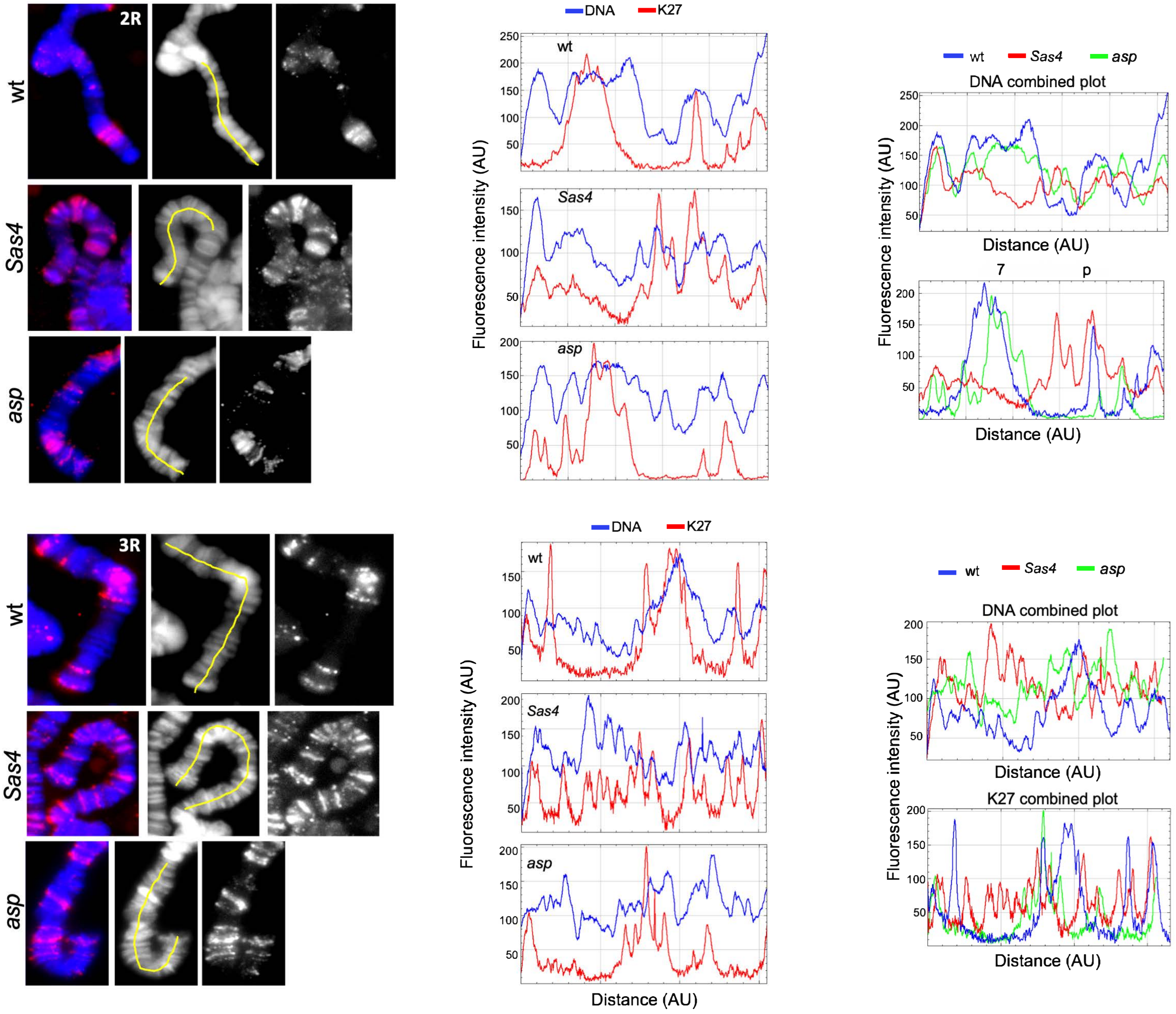

Fig. S9. DNA and H3K27me3 banding patterns are altered in *Sas4* and *asp* mutant polytene chromosomes.

Examples of the 2R and 3R chromosome extremities stained with anti H3K27me3 (red) and DNA (blue) in wt and *Sas4* or *asp* mutant polytene chromosomes with corresponding intensity profiles of DAPI (blue) and H3K27me3 (red) for each genotype. The DNA and H3K27me3 combined plots show the altered patterns in both *Sas4* (red line) and *asp* (green line) mutants compared to wt (blue line); y-axis: fluorescence intensity; x-axis: distance from the tip of the chromosome (arbitrary unit). Scale bar = 5 µm.

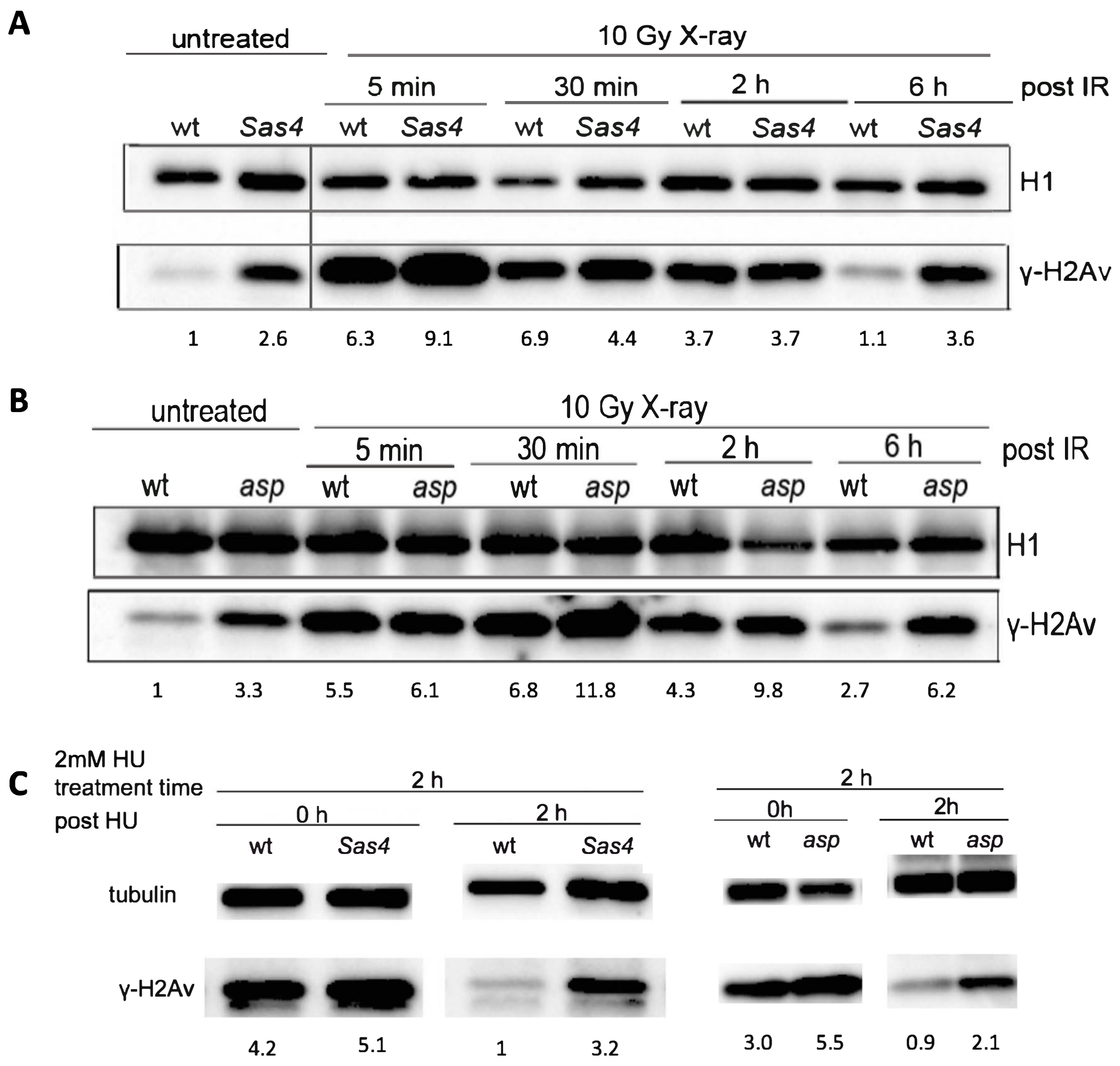

Fig. S10. *Sas4* and *asp* mutant brain cells exhibit delayed DNA Damage Response following irradiation and hydroxyurea treatment.

A) γH2Av levels in wt and *Sas4* larval brain extracts after irradiation with 1 0Gy and dissection after 5 min, 30 min, 2 hours and 6 hours of recovery time. H1 was used as loading control and the γH2Av band quantification was normalized on the loading control. B) γH2Av levels in wt and *asp* larval brain extracts after irradiation with 10Gy and dissection after 5 min, 30 min, 2 hours and 6 hours of recovery time. H1 was used as loading control and the γH2Av band quantification was normalized on the loading control. C) γH2Av levels in wt or *Sas4* and *asp* mutant larval brain extracts detected with γH2Av antibody and tubulin as loading control. Larval brains were treated with 2mM of HU for 2 hours and dissected at 0 and 2 hours post treatment (recovery time). Numbers below the blots indicate the γH2Av band quantification normalized on the loading control (Hl or tubulin).

Development • Supplementary information

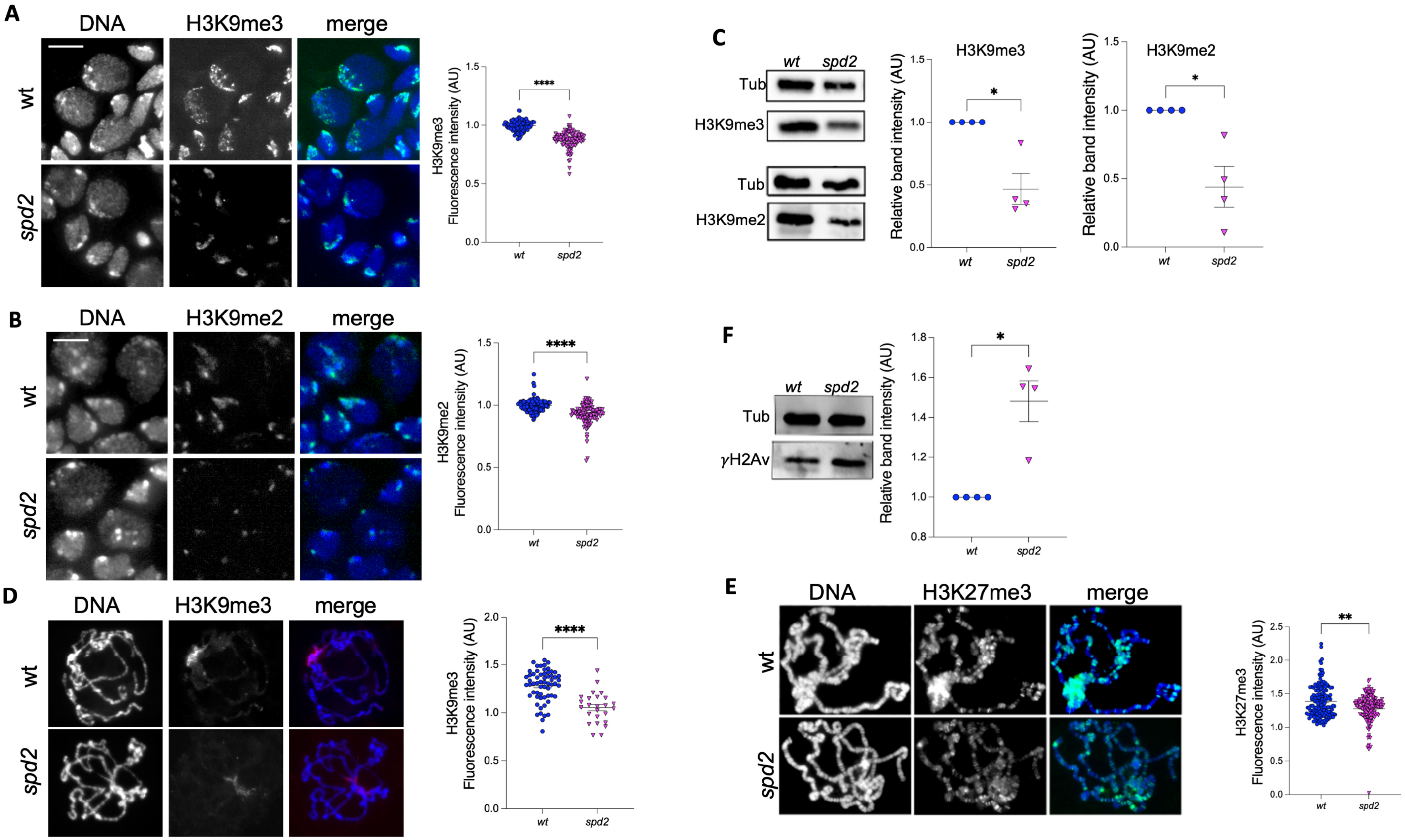
A
DNA
H3K9me3
merge
wt
spd2
H3K9me3 Fluorescence intensity (AU)
****
wt
spd2
B
DNA
H3K9me2
merge
H3K9me2 Fluorescence intensity (AU)
D
H3K9me3 Fluorescence intensity (AU)
C
Tub
H3K9me3
H3K9me2
Relative band intensity (AU)
*
F
γH2Av
E
H3K27me3
H3K27me3 Fluorescence intensity (AU)
**

Fig. S11. ***Spd2*** **mutants display nuclear abnormalities reminiscent of those observed in** ***asp*** **and** ***Sas4*** **mutants.**

A) Brain cells from third instar larval brain squashes of wt and *spd2* mutant stained with anti-H3K9me3 antibody and DAPI (DNA). In merged images H3K9me3 is in green and DNA in blue. Beside the corresponding quantification of H3K9me3 fluorescence intensity per cell is shown. Each dot represents a single cell from at least 3 brains (n≥100). B) Cells from third instar larvae brain squashes of wt and *spd2* mutant stained with anti-H3K9me2 (green) antibody and DAPI (DNA). DNA is in blue. Beside the corresponding quantification of H3K9me2 fluorescence intensity per cell is shown. Each dot represents a single cell from at least three brains (n≥100). C) Representative immunoblots showing decreased levels of both H3K9me3 and H3K9me2 in *Drosophila* larval brain extracts of *spd2* mutant compared to wt, with the corresponding quantification of relative band intensity normalized on loading control (Tub) in at least four independent experiments.
D) Immunolocalization of H3K9me3 on wtand *spd2* mutant polytene chromosomes. In merged images H3K9me3 is in red and DNA (DAPI) is in blue. Beside the corresponding fluorescence intensity quantification of H3K9me3 signals is shown. Each dot represents a single polytene chromosome (n≥25). E) Immunolocalization of H3K27me3 on wtand *spd2* mutant polytene chromosomes. In merged images H3K27me3 is in green and DNA in blue (DAPI). Beside the corresponding fluorescence intensity quantification of H3K27me3 signals is shown. Each dot represents a single polytene chromosome (n≥25). AU, arbitrary unit. Error bars represent SEM. P = p-value calculated using unpaired t test. *p < 0.05; **p < 0.01; ***p < 0.001; ****p < 0.0001. Scale bar =10 µm.

Development • Supplementary information

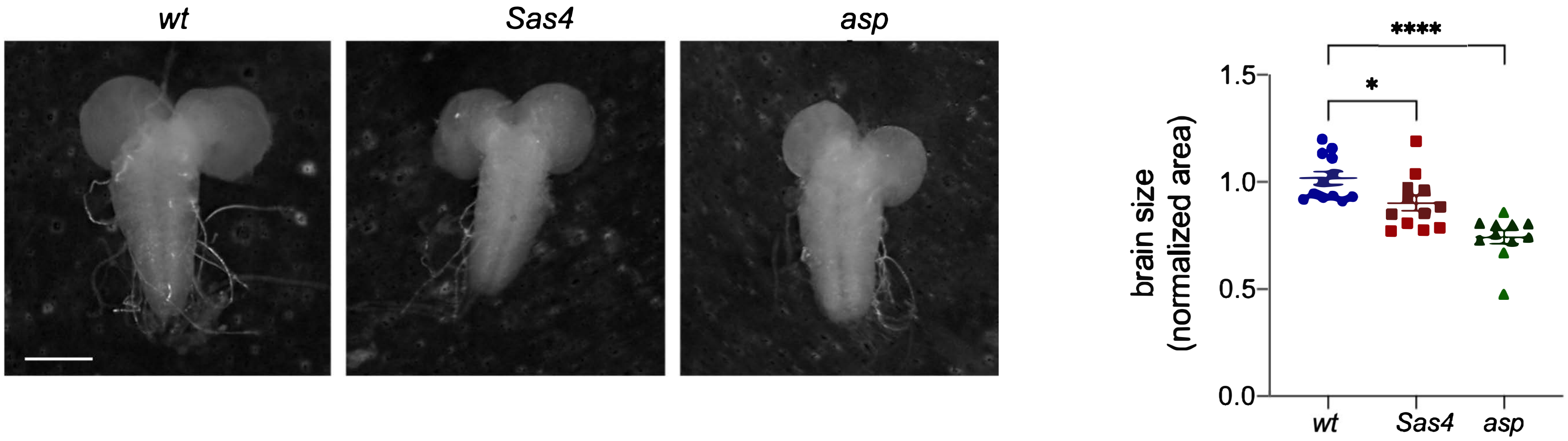

Fig. S12. Loss of Sas4 or Asp leads to reduced brain size.

Representative brain images from synchronized , *Sas4*, and *asp* larvae, with the corresponding size quantification. Each dot represents a single brain (n ≥ 10). Equal numbers of male and female flies, both mutants and wild type, were added to equal amounts of fresh fly food. After one week, when the third instar larvae had reached full size, the larvae were dissected, and brains were collected and scanned to measure their sizes. Brain measurements were performed using Fiji ImageJ software. Error bars represent SEM. P = p-value calculated using unpaired t test. *p < 0.05; **p < 0.01; ***p < 0.001; ****p < 0.0001. Scale bar = 100 .

Table S1. Key resources

Reagent type (species) or resource	Designation	Source or reference	Additional information
Antibody	anti-a tubulin (mouse)	Sigma Aldrich T6199-200UL	IH 1:100 WB 1:5000
Antibody	anti-a tubulin (rabbit)	Abcam ab18251	IH 1:100 WB 1:5000
Antibody	anti-dpn (guinea pig)	Provided by J. Skeath	IH 1:500
Antibody	anti-γH2Av (mouse)	DSHB - UNC93-5.2.1	IH 1:20 WB 1:2000
Antibody	anti-H3K9me3 (rabbit)	Abcam ab8898	IH 1:100 WB 1:2000
Antibody	anti-H3K9me2 (mouse)	Abcam ab1220	IH 1:50 WB 1:400
Antibody	anti- H3K27me3 (rabbit)	Cell Signalling	IH 1:100 WB 1:5000
Antibody	anti- H3K4me3 (rabbit)	Active Motif 61379	IH 1:400 WB 1:5000
Antibody	anti-Giotto (rabbit)	Marzullo et al. (2023)	WB 1:5000
Antibody	anti-H1 (rabbit)	provided by D.V. Fyodorov	WB 1:10,000
Antibody	anti-Dm0lamin (mouse)	DSHB - ADL67.10	IH 1:100 WB 1:1000
Antibody	anti-Asp (rabbit)	Provided by D. Glover	WB 1:1000
Antibody	anti-HP1a (mouse)	DSHB - C1A9	IH 1:10 WB 1:500
Antibody	anti-myosin (rabbit)	Provided by R. Karess Royou et al. (2002)	IH 1:500
Antibody	anti-CID (chicken)	provided by G. Karpen	IH 1:1000
Antibody	goat anti-mouse IgG FITC tagged	Jackson ImmunoResearch 115-095-003	IH 1:20
Antibody	sheep anti-mouse IgG HRP tagged	Sigma Aldrich NA931V	WB 1:5000
Antibody	goat anti-rabbit IgG FITC tagged	Molecular probes life technology A21430	IH 1:300
Antibody	goat anti-rabbit IgG HRP tagged	Sigma Aldrich Cytiva RPN4301	WB 1:5000
Antibody	anti-guinea pig	Invitrogen A21435	IH 1:50
Reagent	PageRuler Plus Protein Ladder	Thermo Scientific 26619	
Reagent	Formaldehyde	Sigma Aldrich F8775	
Reagent	Tris-Buffered Saline (TBS)	Medicago AB 09-7500-100	
Reagent	PBS: Phosphate-Buffered Saline	Sigma Aldrich D5652-1L	
Reagent	Tween® 20	PanReac AppliChem	

Development • Supplementary information

Reagent type (species) or resource	Designation	Source or reference	Additional information
		A4974,0250	
Reagent	Triton X-100	PanReac AppliChem A4975,0500	
Reagent	VECTASHIELD	Vector Laboratories H-1200	
Reagent	Acetic acid	Titolchimica UN2789	
Reagent	Ethanol	Applichem 13186.1212	
Reagent	Bovine Serum Albumin (BSA)	PanReac AppliChem A1391,0250	
Reagent	Non-fat dried milk powder	PanReac AppliChem A830,0500	
Reagent	Normal Goat Serum	JacksonImmunoResearch 005-000-121	
Reagent	Sodium Chloride	PanReac AppliChem A2942,1000	
Reagent	Colchicine	Sigma Aldrich C9754	
Reagent	Hydroxyurea	Sigma Aldrich H8627-5G	
Reagent	Schneider Cell Culture media	Dominique Dutscher L0207-500	
Reagent	Methylstat	Sigma Aldrich SML0343-5MG	
Reagent	Sodium Citrate	Applichem A2403	
Software and algorithms	Prism Graph Pad	Dotmatics	https://www.graphpad.com
Software and algorithms	ImageJ	FUJIFILM Corporation	https://fiji.sc/
