# Supplementary material for: Microcephaly-associated genes asp and Sas4 influence chromatin organization and nuclear lamina structure in Drosophila melanogaster
Source: Development. 2026 May 28;153(10):dev205125. doi: 10.1242/dev.205125 (PMC13286369; doi:10.1242/dev.205125)
Supplement: Supplementary file 1 [file develop-153-205125_review_history.pdf]

Microcephaly-associated genes *asp* and *Sas4* influence chromatin organization and nuclear lamina structure in *Drosophila melanogaster*

Degisew Yinur Mengistu, Marta Marzullo, Claudia Pellacani, Marcella Marchetti, Marta Terribili, Emma Montivero Morales, Maria Patrizia Somma and Laura Ciapponi
DOI: 10.1242/dev.205125

Editor: Thomas Lecuit

Review timeline

Original submission:	23 July 2025
Editorial decision:	19 August 2025
First revision received:	28 January 2026
Editorial decision:	19 February 2026
Second revision received:	18 March 2026
Editorial decision:	31 March 2026
Third revision received:	9 April 2026
Accepted:	9 April 2026

Original submission

First decision letter

MS ID#: dev.205125

MS TITLE: Microcephaly-Associated Genes asp and Sas4 Control Chromatin Organization and Nuclear Lamina Structure in Drosophila melanogaster

AUTHORS: Degisew Yinur Mengistu, Marta Marzullo, Claudia Pellacani, Marcella Marchetti, Marta Terribili, Emma Montivero Morales, Maria Patrizia Somma and Laura Ciapponi

Dear Dr Ciapponi,

I have now received all the referees' reports on the above manuscript, and have reached a decision. The referees' comments are appended below, or you can access them online: please go to: ********

As you will see, the referees express considerable interest in your work, but have some significant criticisms and recommend a substantial revision of your manuscript before we can consider publication. If you are able to revise the manuscript along the lines suggested, which may involve further experiments, I will be happy to receive a revised version of the manuscript. Your revised paper will be re-reviewed by one or more of the original referees, and acceptance of your manuscript will depend on your addressing satisfactorily the reviewers' major concerns. Please also note that Development will normally permit only one round of major revision. If it would be helpful, you are welcome to contact us to discuss your revision in greater detail. Please send us a point-by-point response indicating your plans for addressing the referees' comments, and we will look over this and provide further guidance.

Please attend to all of the reviewers' comments and ensure that you clearly highlight all changes made in the revised manuscript. Please avoid using 'Tracked changes' in Word files as these are lost in PDF conversion. I should be grateful if you would also provide a point-by-point response detailing

© 2026. Published by The Company of Biologists under the terms of the Creative Commons Attribution License (https://creativecommons.org/licenses/by/4.0/).

how you have dealt with the points raised by the reviewers in the 'Response to Reviewers' box. If you do not agree with any of their criticisms or suggestions please explain clearly why this is so.

Reviewer 1

Advance summary and potential significance to field

Autosomal recessive primary microcephaly (MCPH) is a neuro-developmental disorder that derives from a reduced number of neurons and manifests in a smaller brain size. Many MCPH-linked genes encode proteins involved in centrosome biogenesis, including CENPJ and ASPM. It is believed that these proteins affect the asymmetric division of neuronal stem cells, which could lead to imbalances in the number of differentiating neurons.

This manuscript elucidates an interesting connection between the Drosophila homologues of CENPJ (Sas4) and ASPM (Asp) and the configuration of chromatin in larval neuroblasts. Importantly Mengistu et al report an altered methylation state of Histone tails in neuroblasts that lack sas4 or asp, which could have profound consequences for gene expression and ultimately the balance of proliferation versus differentiation of neuronal precursors. They also report that loss of sas 4 or asp results in a higher sensitivity to DNA damage, a phenotype related to MCHP. The observed phenotypes extend our view on how centrosomal components could affect neuronal development. The authors conclude that-at least in flies- sas4 and asp could regulate neuronal cell fate via a cytoskeleton and lamina mediated effect on epigenetic chromatin marks.

However in my opinion this mechanistic link suggested by the authors between Sas4/Asp and chromatin structure is not sufficiently supported. The authors claim that in sas4/asp mutant neuroblasts, cytoskeletal changes lead to a restructured lamina, which then causes epigenetic changes and consequences for DNA repair. While this is possible, more direct mechanisms cannot be excluded. The authors have to strengthen this link to sufficiently backup their conclusions, before the study can be further considered for publication.

Comments for the author

Major points

In Figure 1, S4, S5 the authors describe consequences of sas4/asp loss on NE/lamina organization: While the effects on MT organization and also on the distribution of the LINC protein Klarsicht is somehow clear from Figure 1A-C the conclusions drawn for the lamina are not sufficiently supported by the presented data.

1. First it is unclear how "lamina invaginations" are spotted and quantified. I can see "nucleoplasmic" streaks of Dm0 staining but they could also be present at different z planes in the wild type neuroblasts. The authors should show representative z stacks or z-projections to make this clear in a more rigorous manner. That "MT bundles correlate with Dm0 invaginations in the mutant NBs", is an overstatement, based on what is shown in Figure1D. If not shown clearer, this conclusion cannot be made.

2. I have the same problem when sas4 or asp is depleted by RNAi (Figure S5A). I cannot see this clear difference between wild type (no/few) Dm0 stained invaginations and depletions (many invaginations). The authors have to explain how they score the presence of invaginations and should illustrate this by convincing examples. Again z-stacks could provide clarity.

3. To get a more comprehensive view of the lamina, the authors should document the distribution of the Drosophila C type lamin (where an antibody is available) in sas4/asp neuroblasts.

4. To support their model, the authors should investigate the respective epigenetic marks after perturbations downstream of the centrosome, for example at the level of Microtubules or the lamina. Do modifications of the lamina (by overexpression or depletion of lamins, LEM domain or other INM proteins) cause effects on the respective histone modifications? Would those modifications modify the chromatin phenotypes that the authors see in sas4 or asp mutants?

© 2026. Published by The Company of Biologists under the terms of the Creative Commons Attribution License (https://creativecommons.org/licenses/by/4.0/).

Epistasis experiments could at least genetically relate sas4/asp, the lamina and Histone modifying enzymes.

5. In Figure 3 the authors examine the distribution of the H3K9Me3 labeled heterochromatin in wild type and sas4/asp neuroblasts. They see a general reduction in H3K9Me3 levels and claim that H3K9Me3 loss is specifically pronounced at lamina invaginations. There are 2 problems with this conclusion: 1) As the study suggests that there are hardly any NE invaginations in wild type, the levels of H3K9Me3 at those locations are difficult to compare. 2) The global levels of H3K9Me3 appear so much reduced in the mutants that a specific down-regulation at lamina invaginations is hard to appreciate (Figure 3A).

6. Also in Figure 3 the authors show that Methylstat treatment restores not only H3K9Me3/2 levels but also nuclear morphology and lamina invaginations in sas4/asp mutant cells. We can infer this only from a quantification (Figure 3E), but it would be important to include the actual images in a figure.
Moreover this experiment suggests that the chromatin state instructs NE morphology independent of the cytoskeleton. Although an inter-dependence is possible (perhaps likely), to me this is contrasting the mechanism favored by the authors, namely that the cytoskeleton/lamina affects chromatin configuration. Can the authors please comment on this?

Minor points

There is an issue with the referencing/numbering of the supplementary figures; Figures S4 and S5 are referred to at the beginning of the results section (page 5), much before Figures S1-S3.

Reviewer 2

Advance summary and potential significance to field

The manuscript authored by Degisew Yinur Mengistu et al., describes the function of two Microcephaly-associated disease genes, asp and sas4, in chromatin epigenetic control and nuclear lamina structure. These genes, functionally associated with centrosome function, link centrosome function with chromatin epigenetic control, neuroblasts proliferation, and nuclear lamina integrity. The authors show that loss of either asp or sas4 genes disrupts microtubules organization, nuclear lamina integrity, leads to reduced lamin B and HP1a levels, and causes defective centromere clustering. Further phenotypes include reduction in heterochromatin-associated histone marks, H3K9me2/3 and H3K27me3, elevated DNA damage with delayed repair. Inhibiting denethylases with Methylstat restored H3K9me3 levels. The studies provide novel insights into the pathogenesis of autosomal recessive primary microcephaly (MCPH). Most of the results are well documented and show proper statistical analysis.

Comments for the author

Below are few comments that should addressed by the authors, to improve the results description and conclusions:
1. Functional significance of the mutations in terms of brain size: since the human phenotype is microcephally, and in order to link between the cellular phenotypes described and brain size it would be informative to dissect whether brain size is altered in the mutants.
2. Figure 1A: it is unclear if the figure shows the nucleus or the entire NB cell. It would help to outline the nuclear borders.
3. Figure 1B: Klar staining appears to be non-specific. It is expected to see enrickment along the circumference of the nucleus. The authors should either omit it, or use another LINC marker (e.g. MSP300).
4. Figure 3: it appears that H3K9me3 is reduced in all cells, whereas lamin B is reduced only in a few cells. so the correlation between lamin B reduction and H3K9me3 is not perfect. Are all these cells NBs? Is the phenotype specific to NBs?
5. Figure 6: the chromosome aberration phenotype is interesting. Since this phenotype is observed in salivary gland cells, and therefore is not specific for NBs, the authors should discuss the

© 2026. Published by The Company of Biologists under the terms of the Creative Commons Attribution License (https://creativecommons.org/licenses/by/4.0/).

genes function and the connection to the disease phenotype in a broader view. For example, is it known whether there are phenotypes unrelated to brain size in other tissues.

Reviewer 3

Advance summary and potential significance to field

ASPM/MCPH (asp) and CenpJ/MCPH6 (sas4) are essential for centrosome biogenesis. Their role during mitosis is well established. In the context of neurogenesis, Sas4 and asp mutations associate with microcephaly: the absence of functional Sas4 or asp induce mitotic defects leading to reduced proliferation and premature neuronal differentiation.
In this manuscript, Mengistu et al investigate the role of Drosophila Sas4 and asp in "non-mitotic" processes related to microcephaly pathogenesis, using mutants or using RNAi approaches. They show that:
Sas4 and Asp have are required for the proper organization of the microtubule (MT) cytoskeleton during interphase in Drosophila Neuroblasts. Sas4 and Asp are important for proper brain cells nuclear architecture during interphase: (i) Sas4 and asp mutants are characterized by the presence of abnormal nuclear envelope invaginations (ii) reduced levels of lamin B (iii) reduced levels of heterochromatin marks (iv) increased DNA damage and a delayed response in DNA repair.
These data are potentially interesting, since non-mitotic roles for asp and sas 4 have not been described previously (at the exception of a role in DNA damage).
The authors propose an interesting model where sas4 and asp by controlling interphasic cytoskeleton organization would in turn be crucial for the nuclear architecture and the gene expression programs during brain development.

Comments for the author

The result section could be amended by providing rigorous analyses and restating some of the conclusions (see Concerns below).

In addition, the main conclusion of the manuscript on "a previously unrecognized role for Asp and Sas4 in linking centrosome function to chromatin state" (Abstract) is not fully supported by the data presented.
The data show a correlation between microtubule mis-organization, and nucleus shape abnormalities associated with a reduction of heterochromatin marks, but the causal link between these phenomena is not demonstrated, and only suggested by the alteration of LINC complex bridging cytoskeleton to the nuclear envelope.
Direct investigations on this causal link would greatly improve the impact of the manuscript:
Are the nuclear phenotypes described specific to asp and sas 4 mutations, or can they be reproduced using alternative mutants that alter centrosome/MT organization ?
What would be the effect described of pharmacological perturbations of MT dynamics on the "nuclear phenotypes "described? Cf (Lottersberger et al., 2015) where the link between DNA damage repair and cytoskeleton dynamics has been directly tested using MT -stabilizing and depolymerizing- drugs.
MT stabilizing drug Taxol, often induce MT bundling and might be used to mimic the effect of the sas4 mutant on MT organization: does MT bundling per se would induce nuclear invaginations and alter heterochromatin marks? Finally disrupting the cytoskeleton-nucleus link by silencing LINC complex component is classically used and may be informative too.

Major concern 1: Cytoskeleton organization

* Sas4 and asp are both essential for proper centrosome assembly and function. A full description of the centrosome shape and position abnormalities, should be added to fully describe the MT network mis-organization and help the reader to understand the cytoskeleton architectures depicted in the figures. Moreover, in absence of such piece of data, it is hard to judge whether "In sas4 mutant NB, the centrosome is not detectable" (page 5).

* In the case of mutants, the microtubule organization in the selected cells in Figure 1A, Figure 1G and Figure 2A are not similar. It is therefore not clear to me (1) whether mutant cells lack a clear

© 2026. Published by The Company of Biologists under the terms of the Creative Commons Attribution License (https://creativecommons.org/licenses/by/4.0/).

wild-type "aster like"organization, contain only MT bundles, and (2) where the T bundles are located within the cell. In figure 1A, bundles are only found at the periphery of the mutant cells. In contrast, in Figure 1D et E, MT bundles associate with nuclear invaginations. The data as well as the text describing the MT organization should be clarified.

* "In asp mutant NBs, the MT network appears disorganized and consists of short, fragmented MTs" (Figure 1A). In figure 1A, a diffuse signal in the cytoplasm and some bundle-like structures at the cell periphery can be observed: what is the argument to conclude on the presence of fragmented MTs? Morever, according to Garcez et al, sas4/cenpJ deletion result in MT stabilization which is not compatible with fragmentation.

*The quantifications (line scans) presented in 1A are poorly informative. For such semi quantitative analysis, reference marks (cell membrane + nuclear envelope markers) should be added to the analysis in order to understand where the MT bundles are located within the cell. In particular, since the authors investigate nuclear morphology and architecture in the following figures, it is crucial to assess the interactions between centrosome/MT and the nuclear envelope in WT versus mutant conditions.
In this line, the DAPI staining unfortunately appears weak: it is difficult to clearly visualize the nuclear boundaries in the figure. Maybe zooming on the nuclei of interest, as done in other figures, would be helpful.

* In other experimental models, actomyosin cytoskeleton has been shown to be tightly associated with the nucleus and involved in mechano-transduction pathways (Davidson et al , 2021 doi.org/10.1016/j.tcb.2020.11.009). Acto-myosin defects have been documented in Drosophila asp mutant brain cells (Rujano et al., 2013 DOI: 10.1038/ncb2858) and Actin-based deformations of the nucleus have recently been shown be involved in mouse multi-ciliated ependymal cell differentiation (Basso et al, 2025 doi.org/10.1016/j.devcel.2024.11.008). In the light of this literature, it would be important to assess the role of actin cytoskeleton in the processes described in the present manuscript.
Is acto-myosin network organization altered in asp and sas4 mutant cells? If yes, what iq its contribution to the nuclear architecture?

Main concern 2. Nucleus morphology

* In addition to the effect of Asp and sas4 mutations on nucleus inbaginations, is there any effect on the nucleus size ? This point should be adressed, since the figure presented (figure 1A and E) suggests that the nuclei in sas4 mutant are smaller than in other conditions.

In addition, the authors should clarify the method they use to identify NB Nucleus. Neuroblasts are identified according to the nucleus size (diameter > 10 μm), I have no objection except that this method is not justified in the text. A reference should be added, or supp data should be provided to validate their shift from Deadpan staining to nucleus size estimation.

* In Figure 1B. Wild type, Klar decorates nucleus envelope « while Sas4 and asp mutant cells showed Klar dispersed throughout the nucleus (Fig. 1B and C)"
This point should be clarified since it appears to me that the signal is homogenously distributed in the nucleus and in the cytoplasm and not only in the nucleoplasm: Is this protein improper localization or reduced expression? A western blot analysis would be informative (as in figure 1G).

* On nuclear envelope function
Page 6 "Loss of Asp and Sas4 (..) leads to nuclear envelope dysfunction in larval brain cells"
What do the authors mean by Nuclear envelope dysfunction: what are the processes affected? (The global reduction of heterochromatin marks shown in this section could indeed rely on mechanisms that are independent of the nuclear envelope). This point should be clarified.

As far as Cid clustering is concerned, this process is described to occur at prophase, thus to my understanding, when the nuclear envelope starts to be dismantled : how does this process reflect nuclear envelope activity ? The authors should argument more precisely on this point.

© 2026. Published by The Company of Biologists under the terms of the Creative Commons Attribution License (https://creativecommons.org/licenses/by/4.0/).

Page 7 "Taken together, these data indicate that loss of either Sas4 or Asp reduces Lamin levels and disrupts lamin nucleoskeleton architecture, potentially impairing nuclear envelope integrity »
Such conclusion raises the question of Nuclear integrity (absence of discontinuities / breaks) in the mutants. Nuclear integrity can be easily assessed by the presence of nuclear blebs (see for instance Maning et al 202 DOI : 10.1080/19491034.2025.2486816), or by visualizing discontinuities in the lamin staining. It would be interesting to analyze this parameter to support this conclusion.

* In the graphical abstract
-The MT network should be differently drawn in the mutant panel (bundles associated with the nucleus envelope or anything reflecting the phenotypes observed)
- Lamin invaginations are represented under an undeformed nuclear envelope: the nuclear envelope should lie on the lamin network and be curved accordingly.
-In addition, a unique and large invagination seems to be present most of the time in the mutants This is what should be drawn instead of the multiple and tiny "wrinkles".

Main concern 3. Heterochromatin remodeling

*The authors propose that asp and sas 4 mutations "affect heterochromatin organization" (title page 6). Nevertheless, the data presented in the brain cells indicate a global reduction of heterochromatin marks (Immunostaining + Western blot) rather than a spatial reorganization in the nucleoplasm.
Moreover, in Figure 2 H3K9me3 an 2 signals in the mutants remain in the periphery of the nucleoplasm, as in WT : the spatial distribution of the remaining marks appears thus unchanged. Confusingly, the spatial distribution of H3K9me3shown in figure 3, does not appear to be consistent with the figure 2.
A clarification on this point is thus required.

The authors claim that "in mutant brains (...), the nuclear envelope invaginations were consistently associated with a marked reduction of these heterochromatic marks "(Figure 3A and B). The cells selected an presented in the insets do not support an "association" -in the sense of " co-localization"- of invaginations with a local reduction of the H2K9me2 and me3 marks. A clarification on this point is required too.

In conclusion, the spatial distribution of the chromatin marks should be analyzed quantitatively more deeply in the brain cells (distribution area at the periphery as done for the cid, signal distance to invagination etc) in order to support any conclusion on heterochromatin organization.

*As far as results obtained with salivary glands. I understand the interest of the model for the pattern analysis, but I must confess that I do not see the link with brain development "These changes in the DNA banding pattern further support our hypothesis that Asp and Sas4 loss disrupts chromatin organization, potentially altering gene expression programs during brain development" (page 6).
This point maybe more argumented in the text.

*Finally, H3K9me3 and 2 levels appear to be different in asp and sas4 mutants (figure 2): could the the authors comment on that point ?

Minor concerns

* Figure 6 Legend
Each dot represents a single polytene chromosome ($n \geq 15$).
The exact value of n and the number of replicates should be provided.

* Figure 6.E
The linescan profiles are confusing : A normalization of the K27 signal intensity on DAPI signal should be more informative. In addition the x and y axes are not properly labelled.

* Figure 7
"Previous work has shown that the human orthologues of Sas4 and asp, (McIntyre et al. 2012; Williams et al., 2015) (…) function but also to prevent DNA damage."

© 2026. Published by The Company of Biologists under the terms of the Creative Commons Attribution License (https://creativecommons.org/licenses/by/4.0/).

The references are not properly quoted: the two publications are based on mouse models not on human.
In addition Drosophila sas4 deletion associates with increased DNA damage in (Poulton et al 2015 DOI: 10.1016/j.devcel.2014.08.007) , this publication could be mentioned.

Fig.7 B " Graphical representation of the percentage of γH2Av positive cells per field, as shown in (A). n≥35 for 3 brains."
The exact value of n should be provided.

* Figure 7 and 8 may be fused to a present a full and coherent

---

First revision

Author response to reviewers' comments

LINES are referred to the track-changes version of the revised manuscript

Reviewer 1: SUMMARY OF THE ADVANCE MADE IN THIS PAPER AND ITS POTENTIAL SIGNIFICANCE TO THE FIELD

Autosomal recessive primary microcephaly (MCPH) is a neuro-developmental disorder that derives from a reduced number of neurons and manifests in a smaller brain size. Many MCPH-linked genes encode proteins involved in centrosome biogenesis, including CENPJ and ASPM. It is believed that these proteins affect the asymmetric division of neuronal stem cells, which could lead to imbalances in the number of differentiating neurons.
This manuscript elucidates an interesting connection between the Drosophila homologues of CENPJ (Sas4) and ASPM (Asp) and the configuration of chromatin in larval neuroblasts. Importantly Mengistu et al report an altered methylation state of Histone tails in neuroblasts that lack sas4 or asp, which could have profound consequences for gene expression and ultimately the balance of proliferation versus differentiation of neuronal precursors. They also report that loss of sas 4 or asp results in a higher sensitivity to DNA damage, a phenotype related to MCHP. The observed phenotypes extend our view on how centrosomal components could affect neuronal development. The authors conclude that-at least in flies- sas4 and asp could regulate neuronal cell fate via a cytoskeleton and lamina mediated effect on epigenetic chromatin marks.

However in my opinion this mechanistic link suggested by the authors between Sas4/Asp and chromatin structure is not sufficiently supported. The authors claim that in sas4/asp mutant neuroblasts, cytoskeletal changes lead to a restructured lamina, which then causes epigenetic changes and consequences for DNA repair. While this is possible, more direct mechanisms cannot be excluded. The authors have to strengthen this link to sufficiently backup their conclusions, before the study can be further considered for publication.

SUGGESTIONS TO AUTHORS

Major points

In Figure 1, S4, S5 the authors describe consequences of sas4/asp loss on NE/lamina organization: While the effects on MT organization and also on the distribution of the LINC protein Klarsicht is somehow clear from Figure 1A-C the conclusions drawn for the lamina are not sufficiently supported by the presented data.

1. First it is unclear how "lamina invaginations" are spotted and quantified. I can see "nucleoplasmic" streaks of Dm0 staining but they could also be present at different z planes in the wild type neuroblasts. The authors should show representative z stacks or z-projections to make this clear in a more rigorous manner. That "MT bundles correlate with Dm0 invaginations in the mutant NBs", is an overstatement, based on what is shown in Figure1D. If not shown clearer, this

© 2026. Published by The Company of Biologists under the terms of the Creative Commons Attribution License (https://creativecommons.org/licenses/by/4.0/).

conclusion cannot be made.
We thank the referee for raising this issue. Cells with Lamin invaginations were quantified as those displaying a disorganization of the Lamin network, characterized by a Lamin signal extending into the nucleoplasm in the form of inward projections or streams (Paonessa et al., 2019). **Lines 134-137**. This definition has now been included in the manuscript. To better explain this point, all z-stacks corresponding to the panels shown in Figure 1 are now included in Supplementary Figure 2. These images clearly show that no inward extensions or streams of Lamin within the nucleoplasm were present in any of the z-stacks from wild-type cells, whereas in mutant cells Lamin signals were consistently observed as inward extensions or streams within the nucleoplasm, rather than being restricted to the nuclear periphery.
In Supplementary Figure 2 we have now included the representative z-stacks corresponding to each panel of Figure 1A, D and E.
In relation to the MT bundles, we have modified our statement in the revised manuscript to present a milder hypothesis. **Lines 141-143.**
2. I have the same problem when sas4 or asp is depleted by RNAi (Figure S5A). I cannot see this clear difference between wild type (no/few) Dm0 stained invaginations and depletions (many invaginations). The authors have to explain how they score the presence of invaginations and should illustrate this by convincing examples. Again z-stacks could provide clarity.
We thank the referee for bringing this issue to our attention. We acknowledge that the original image was not of adequate quality; therefore, it has been replaced with a more representative and clearer one (now Supplementary figure S5 A and B) with the corresponding z-stacks. The strategy used for the scoring of nuclear invaginations is described in detail in the previous point.

3. To get a more comprehensive view of the lamina, the authors should document the distribution of the Drosophila C type lamin (where an antibody is available) in sas4/asp neuroblasts.
We agree with the referee that this experiment would help determine whether the invagination phenotype is restricted to a specific Lamin isoform. Accordingly, we performed immunofluorescence experiments using an antibody against Lamin C (shown in Supplementary Figure S4E–F). This experiment revealed the same altered nuclear morphology in both *Sas4* and *asp* mutant cells, indicating that the phenotype is not limited to a single Lamin isoform but rather reflects a broader alteration of the nuclear membrane where Lamin proteins are associated. **Lines 137-141.**

4. To support their model, the authors should investigate the respective epigenetic marks after perturbations downstream of the centrosome, for example at the level of Microtubules or the lamina. Do modifications of the lamina (by overexpression or depletion of lamins, LEM domain or other INM proteins) cause effects on the respective histone modifications? Would those modifications modify the chromatin phenotypes that the authors see in sas4 or asp mutants? Epistasis experiments could at least genetically relate sas4/asp, the lamina and Histone modifying enzymes.
We agree with the referee that epistasis experiments could, in principle, provide a genetic link between Sas4/Asp, the nuclear lamina, and histone-modifying enzymes. However, such experiments are technically demanding and time-consuming, as they require the generation of complex genotypes involving multiple chromosomes and carefully balanced stocks. Moreover, although the *Drosophila* research community is well known for its willingness to share fly lines, the importation of fly stocks into Italy is often complicated by customs-related issues, which can substantially delay this process.
That said, we respectfully believe that depletion or overexpression of LamDm0 in these mutant backgrounds would be unlikely to provide substantial additional mechanistic insight, as Lamin depletion is already well established to cause heterochromatin loss and reduced H3K9 methylation (Frost et al., 2016).

Nonetheless, to genetically address the referee's point and to test whether restoration of heterochromatin marks could ameliorate the phenotype caused by microtubule-binding protein depletion, we pursued a complementary genetic approach using a fly line overexpressing Su(var)3-9 methylase, that was already in our hands. Specifically, we combined *asp* or *Sas4* RNAi with *Su(var)3-9* overexpression driven by tubulin- GAL4 and assessed both H3K9me3 levels and the presence of nuclear invaginations.
In the tubulin-GAL4 driven UAS-*Su(var)3-9; UAS-asp*^{RNAi} combination, as expected we observed a

© 2026. Published by The Company of Biologists under the terms of the Creative Commons Attribution License (https://creativecommons.org/licenses/by/4.0/).

robust restoration of H3K9me3 levels; however, this was not accompanied by a reduction in the frequency of nuclear invaginations.
Unfortunately, we were unable to obtain viable individuals carrying both the UAS-*Su(var)3-9* overexpression construct and the UAS-*Sas4*^{RNAi} transgene, even in the absence of GAL4-driven expression. This suggests an unexpected genetic incompatibility or background-dependent lethality associated with the combined presence of these transgenes, rather than an effect of transgenes induction. Despite multiple independent crossing attempts and careful stock balancing, this limitation could not be resolved.
However, this technical limitation does not undermine the conclusions supported by the analysis of the UAS-*Su(var)3-9; UAS-asp*^{RNAi} combination. These results clearly demonstrate that the observed nuclear invaginations arise as a direct consequence of microtubule-binding protein depletion, reflecting cytoskeletal instability rather than defects in the Su(var)3-9 mediated H3K9 methylation *per sé*. These results have been included in the revised manuscript and the relative figure included as Supplementary Figure 7. **Lines 192-205.**

5. In Figure 3 the authors examine the distribution of the H3K9Me3 labelled heterochromatin in wild type and sas4/asp neuroblasts. They see a general reduction in H3K9Me3 levels and claim that H3K9Me3 loss is specifically pronounced at lamina invaginations. There are 2 problems with this conclusion: 1) As the study suggests that there are hardly any NE invaginations in wild type, the levels of H3K9Me3 at those locations are difficult to compare. 2) The global levels of H3K9Me3 appear so much reduced in the mutants that a specific down-regulation at lamina invaginations is hard to appreciate (Figure 3A).
We are extremely grateful to the reviewer for highlighting this important point. We wish to clarify that H3K9me3 was quantified at the whole-cell level, distinguishing cells with Lamin invaginations from those without, rather than specifically at the invagination sites (Figure 3A, B). We apologize to the reviewer for the misleading wording in our original statement. In the revised manuscript, we now explain that in mutant brains co-stained for LaminDm0 and H3K9me3, cells with clear envelope invaginations consistently displayed lower overall H3K9me3 levels compared to mutant cells without invaginations (Figure 3B), indicating that reduced H3K9me3 occurs at the whole-cell level and is not restricted to nuclear envelope invaginations sites. **Lines 177-182.**

6. Also in Figure 3 the authors show that Methylstat treatment restores not only H3K9Me3/2 levels but also nuclear morphology and lamina invaginations in sas4/asp mutant cells. We can infer this only from a quantification (Figure 3E), but it would be important to include the actual images in a figure. Moreover, this experiment suggests that the chromatin state instructs NE morphology independent of the cytoskeleton. Although an inter-dependence is possible (perhaps likely), to me this is contrasting the mechanism favoured by the authors, namely that the cytoskeleton/lamina affects chromatin configuration. Can the authors please comment on this?
We are extremely grateful to the reviewer for suggesting the inclusion of this image. The comparison of brain cells before and after Methylstat treatment clearly highlights the significant increase in H3K9me3 deposition and the reduction of Lamin invaginations following treatment (figure 3C). Previously, this effect was shown only through graphical representations, which made the result highly technical. The new image now unambiguously shows the increased number of red spots for H3K9 methylation and the reduced disorganization of the Lamin network after treatment, making the biological message much more evident. Regarding the reviewer's mechanistic point, we agree that these findings suggest a bidirectional relationship between chromatin organization and nuclear architecture. While the loss of Sas4 or Asp primarily perturbs the cytoskeleton and nuclear lamina, consequently altering chromatin compaction, inhibition of demethylases with Methylstat causes a global increase in methylation that intensifies nuclear stiffness and potentially counteracts the nuclear invaginations caused by microtubule dysfunction. **Lines 188-191 and Lines 357-359.**

Minor points
There is an issue with the referencing/numbering of the supplementary figures; Figures S4 and S5 are referred to at the beginning of the results section (page 5), much before Figures S1-S3.
We thank the reviewer for this observation. In the revised manuscript, the numbering of the supplementary figures has been adjusted to follow the order in which they are described in the text.

© 2026. Published by The Company of Biologists under the terms of the Creative Commons Attribution License (https://creativecommons.org/licenses/by/4.0/).

Reviewer 2: SUMMARY OF THE ADVANCE MADE IN THIS PAPER AND ITS POTENTIAL SIGNIFICANCE TO THE FIELD
The manuscript authored by Degisew Yinur Mengistu et al., describes the function of two Microcephaly- associated disease genes, asp and sas4, in chromatin epigenetic control and nuclear lamina structure. These genes, functionally associated with centrosome function, link centrosome function with chromatin epigenetic control, neuroblasts proliferation, and nuclear lamina integrity. The authors show that loss of either asp or sas4 genes disrupts microtubules organization, nuclear lamina integrity, leads to reduced lamin B and HP1a levels, and causes defective centromere clustering. Further phenotypes include reduction in heterochromatin-associated histone marks, H3K9me2/3 and H3K27me3, elevated DNA damage with delayed repair. Inhibiting demethylases with Methylstat restored H3K9me3 levels. The studies provide novel insights into the pathogenesis of autosomal recessive primary microcephaly (MCPH).
Most of the results are well documented and show proper statistical analysis.

SUGGESTIONS TO AUTHORS
Below are few comments that should addressed by the authors, to improve the results description and conclusions:

1. Functional significance of the mutations in terms of brain size: since the human phenotype is microcephaly, and in order to link between the cellular phenotypes described and brain size it would be informative to dissect whether brain size is altered in the mutants.
We thank the referee for this question. Previous studies have shown that larval brains of *asp* mutants are smaller, thereby phenocopying the human microcephaly phenotype. Now we have examined larval brains from both *Sas4* and *asp* mutations and performed quantitative analyses of their size. These analyses confirm a significant reduction in brain size in both *Sas4* and *asp* mutant brains compared to controls. The corresponding images and quantifications are now provided as Supplementary Figure12, allowing a direct comparison of the effects of these mutations on larval brain size.

2. Figure 1A: it is unclear if the figure shows the nucleus or the entire NB cell. It would help to outline the nuclear borders.
We thank the reviewer for this suggestion. In the revised manuscript, we have outlined the nuclear borders in Figure 1A (and in figures 1D and 1E) to clarify that the images show the entire neuroblast, allowing a clearer distinction between the nucleus and the rest of the cell.

3. Figure 1B: Klar staining appears to be non-specific. It is expected to see enrichment along the circumference of the nucleus. The authors should either omit it, or use another LINC marker (e.g. MSP300).
We appreciate the reviewer's comment regarding Figure 1B. To ensure the robustness of our conclusions, we have performed an additional experiment using an alternative anti-Klar antibody (Klar-M). We found that in wild-type neuroblasts, Klar-M is predominantly enriched at the nuclear envelope, whereas in *Sas4* and *asp* mutant cells, Klar appears dispersed throughout the nucleus. Importantly, the results obtained with Klar-M fully corroborate those observed with Klar-C, confirming that Klar is dispersed in mutant cells. As Klar-M provided a clearer and more specific labelling, these images and their quantification are now included in the revised manuscript as Figure 1B and C, while the Klar-C data have been moved to Supplementary Figure 4A and B.

4. Figure 3: it appears that H3K9me3 is reduced in all cells, whereas lamin B is reduced only in a few cells. So, the correlation between lamin B reduction and H3K9me3 is not perfect. Are all these cells NBs? Is the phenotype specific to NBs?
We thank the reviewer for raising this important point. As this figure was intended solely to illustrate nuclear envelope invaginations rather than to quantify Lamin levels, the image contrast was adjusted (for both wild- type and mutant samples) to enhance visualization. We have now included in the revised manuscript the corresponding images with the original intensity levels, and, as requested by Reviewer 1, we also added the images relative to the Methylstat experiment for direct comparison. Moreover, a graphical representation of Lamin intensity, illustrating a general reduction of Lamin levels in mutant larval brain cells (a phenotype not restricted to neuroblasts), is now presented in Figure 1G.

© 2026. Published by The Company of Biologists under the terms of the Creative Commons Attribution License (https://creativecommons.org/licenses/by/4.0/).

Finally, we would like to clarify that the fields shown in Figure 3 contain only differentiated larval brain cells, with no neuroblasts present, whereas in Figure 1 the cells indicated by dashed squares correspond to neuroblasts, as specified in the figure legends. We apologize to the reviewer for not having consistently specified when the images refer to neuroblasts and when they represent differentiated brain cells. We have now revised the manuscript accordingly, and both the main text and figure legends clearly distinguish between these two types of cells.

5. Figure 6: the chromosome aberration phenotype is interesting. Since this phenotype is observed in salivary gland cells, and therefore is not specific for NBs, the authors should discuss the genes function and the connection to the disease phenotype in a broader view. For example, is it known whether there are phenotypes unrelated to brain size in other tissues.
We thank the reviewer for this comment. However, we are not entirely sure we fully understand the specific concern. Typically, a "chromosome aberration" phenotype refers to mitotic chromosome breaks, translocations, or similar structural defects.
In this case, however, we believe the reviewer is referring to the chromatin alterations observed in mutant polytene chromosomes shown in Figure 6. Polytene chromosomes were used solely to assess changes in chromatin banding patterns using specific antibodies against different chromatin marks, rather than to identify structural chromosome aberrations. These analyses were intended to provide additional insight into the alterations of nuclear organization observed in the mutant cells. There is extensive literature regarding the consistency of data between polytene chromosomes chromatin organization and other *Drosophila* tissues (Vatolina et al, 2011; Demakov et al, 2011; Eagen et al, 2015). Thus, we believe that data obtained from salivary gland can be translated to CNS. Although polytene chromosome analysis is not quantitative, it allows precise visualization of bands, interbands, and their associated chromatin marks. **Lines 251-254**.

Reviewer 3: SUMMARY OF THE ADVANCE MADE IN THIS PAPER AND ITS POTENTIAL SIGNIFICANCE TO THE FIELD
ASPM/MCPH (asp) and CenpJ/MCPH6 (sas4) are essential for centrosome biogenesis. Their role during mitosis is well established. In the context of neurogenesis, Sas4 and asp mutations associate with microcephaly: the absence of functional Sas4 or asp induce mitotic defects leading to reduced proliferation and premature neuronal differentiation.
In this manuscript, Mengistu et al investigate the role of Drosophila Sas4 and asp in "non-mitotic" processes related to microcephaly pathogenesis, using mutants or using RNAi approaches. They show that:
Sas4 and Asp are required for the proper organization of the microtubule (MT) cytoskeleton during interphase in Drosophila Neuroblasts. Sas4 and Asp are important for proper brain cells nuclear architecture during interphase: (i) Sas4 and asp mutants are characterized by the presence of abnormal nuclear envelope invaginations (ii) reduced levels of lamin B (iii) reduced levels of heterochromatin marks (iv) increased DNA damage and a delayed response in DNA repair.
These data are potentially interesting, since non-mitotic roles for asp and sas 4 have not been described previously (at the exception of a role in DNA damage).
The authors propose an interesting model where sas4 and asp by controlling interphasic cytoskeleton organization would in turn be crucial for the nuclear architecture and the gene expression programs during brain development.

SUGGESTIONS TO AUTHORS
The result section could be amended by providing rigorous analyses and restating some of the conclusions (see Concerns below).
In addition, the main conclusion of the manuscript on "a previously unrecognized role for Asp and Sas4 in linking centrosome function to chromatin state" (Abstract) is not fully supported by the data presented.
The data show a correlation between microtubule mis-organization, and nucleus shape abnormalities associated with a reduction of heterochromatin marks, but the causal link between these phenomena is not demonstrated and only suggested by the alteration of LINC complex bridging cytoskeleton to the nuclear envelope.
Direct investigations on this causal link would greatly improve the impact of the manuscript: Are the nuclear phenotypes described specific to asp and sas 4 mutations, or can they be reproduced using alternative mutants that alter centrosome/MT organization?

© 2026. Published by The Company of Biologists under the terms of the Creative Commons Attribution License (https://creativecommons.org/licenses/by/4.0/).

We thank the reviewer for this constructive comment and for highlighting the importance of establishing whether the phenotypes observed are specific to *asp* and *Sas4* mutations or reflect a broader consequence of centrosome or microtubule disorganization.
To address this point, we analysed mutants for another centrosomal gene, *spindle defective 2 (spd2)*, the *Drosophila* ortholog of human *CEP192*, which encodes a key protein required for pericentriolar material recruitment and MT nucleation from the centrosomes and has well-established roles in centrosome assembly and MT organization in neuroblasts.
Interestingly, we found that *spd2* mutants display defects reminiscent of those observed in *asp* and *Sas4* mutants. These findings are detailed in an additional paragraph and figure (Supplementary Figure S11) and have been considered into the discussion section of the revised manuscript. **Lines 291-304.**

What would be the effect described of pharmacological perturbations of MT dynamics on the "nuclear phenotypes "described? Cf (Lottersberger et al., 2015) where the link between DNA damage repair and cytoskeleton dynamics has been directly tested using MT -stabilizing and depolymerizing- drugs.
MT stabilizing drug Taxol, often induce MT bundling and might be used to mimic the effect of the sas4 mutant on MT organization: does MT bundling per se would induce nuclear invaginations and alter heterochromatin marks?
We thank the referee for raising this point. Indeed, Taxol is known to hyper-stabilize microtubules, promoting the formation of rigid bundles and inducing nuclear envelope deformations such as invaginations. These MT-dependent nuclear deformations have been reported to influence chromatin organization and dynamics (Hampoelz et al., 2011 https://doi.org/10.1242/dev.065706; Ramdas and Shivashankar, 2015, https://doi.org/10.1016/j.jmb.2014.09.008).
As suggested, we performed acute (4-hour) treatment of dissected wild-type larval brains with Taxol and examined nuclear envelope morphology (LaminDm0) and tubulin. Consistent with previous reports, Taxol treatment induced nuclear envelope deformations/invaginations (7.9% in untreated *versus* 35.5% in treated brain cells; n > 200). However, we have not included these data in the revised manuscript, as these effects have been well documented in the literature, and most importantly, we believe that short-term pharmacological perturbations cannot fully recapitulate the developmental consequences of *Sas4* or *asp* mutations, which affect microtubule behaviour throughout neurogenesis.

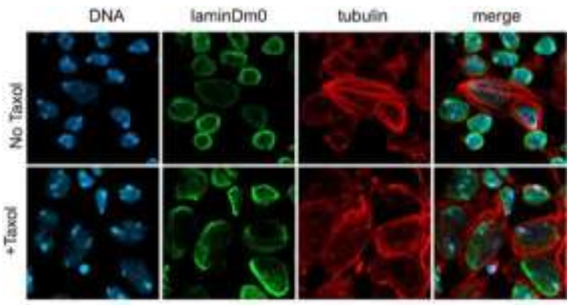

Finally disrupting the cytoskeleton-nucleus link by silencing LINC complex component is classically used and may be informative too.
We thank the referee for this comment. However, we are not entirely sure we fully understand the specific experimental aim of this suggestion. Disruption of the LINC complex is known to disrupt perinuclear microtubule organization (Gimpel et al., 2017, https://doi.org/10.1016/j.cub.2017.08.031; Sun et al., 2019, https://doi.org/10.1016/j.devcel.2019.03.027; Leong et al., 2023, https://doi.org/10.1093/hmg/ddac179; Pavlov et al., 2024 https://doi.org/10.1038/s44161-025-00727-w; Padilla et al., 2025, https://doi.org/10.1242/jcs.263712), which would likely reproduce rather than clarify the effects observed in *asp* and *Sas4* mutants. Consequently, analysing LINC-depleted larval brain cells would not allow us to disentangle the specific contribution of Asp and Sas4 to nuclear architecture. Moreover, combining RNAi for Sas4 or Asp with depletion of a LINC component would likely exacerbate the overall phenotype, without providing additional mechanistic insight into their individual roles.

Major concern 1: Cytoskeleton organization

© 2026. Published by The Company of Biologists under the terms of the Creative Commons Attribution License (https://creativecommons.org/licenses/by/4.0/).

* Sas4 and asp are both essential for proper centrosome assembly and function. A full description of the centrosome shape and position abnormalities should be added to fully describe the MT network mis- organization and help the reader to understand the cytoskeleton architectures depicted in the figures. Moreover, in absence of such piece of data, it is hard to judge whether "In sas4 mutant NB, the centrosome is not detectable" (page 5).
We thank the reviewer for this observation and apologize for not having clearly described the centrosome phenotype in our original submission. We have now included a brief description of centrosome behaviour in *Sas4* and *asp* mutant neuroblasts and reported the appropriate references. Sas4 is known to be required for centriole duplication, and consequently, larval brain cells from *Sas4* mutants completely lack centrosomes (Basto et al., 2006). Differently, Rujano et al. (2013) demonstrated that depletion of Asp does not affect centrosome localization during interphase. This information is now clearly reported also in the corresponding figure legends. Our findings provide the first evidence describing the organization of the microtubule cytoskeleton in *Sas4* and *asp* mutant NBs during interphase. **Lines 98-112.**
* In the case of mutants, the microtubule organization in the selected cells in Figure 1A, Figure 1D and Figure 2A are not similar. It is therefore not clear to me (1) whether mutant cells lack a clear wild-type "aster like" organization, contain only MT bundles, and (2) where the T bundles are located within the cell.
We thank the reviewer for this notable observation. The apparent differences in microtubule organization among the cited panels reflect both the cell phase and the experimental conditions shown. Specifically, Figures 1A and 1D display interphase neuroblast cells, whereas Figure 2A shows late G2/early prophase neuroblasts. These specifics have been now indicated in the figure legends. In addition, the variation between Figures 1A and 1D arises from the use of different anti-tubulin antibodies: a mouse anti-tubulin antibody was used in Figure 1A, while a rabbit anti-tubulin antibody was used in Figure 1D.

1) In the interphase NBs shown in Figure 1, a centrosome is visible only in the wild-type cell. This is expected, since *Sas4* mutant NBs lack centrioles and therefore cannot assemble a centrosome. In *asp* mutant NBs, the centrosome is only faintly detectable, in line with previous reports (Rujano et al., 2013). Differently, Figure 2 shows late G2/early prophase NBs, when asters begin to form around centrosomes. At this stage, asters are clearly visible in wild-type NBs, absent in Sas4 mutants, and strongly reduced in asp mutants. This information is now clearly reported in the corresponding figure legends.

2) We have now outlined the nuclear boundaries in Figures 1A and 1D to better illustrate the localization of MTs within the cells. MT bundles are observed exclusively in the cytoplasm surrounding the nuclei of *Sas4* mutant NBs (Figures 1A, D). In *asp* mutant NBs, the MT network extends throughout the entire cell, similar to wild type. It is also worth noting that the cytoplasm of *Drosophila* larval NBs is extremely thin, resulting in most of the cytoskeleton being concentrated near the nucleus and nuclear lamina. This characteristic enables clear visualization of the spatial association between MTs and lamina invaginations.

In figure 1A, bundles are only found at the periphery of the mutant cells. In contrast, in Figure 1D et E, MT bundles associate with nuclear invaginations.
The data as well as the text describing the MT organization should be clarified.
We thank the reviewer for this valuable observation. We have revised the text to more clearly describe the organization of MTs in both wild-type and mutant neuroblasts. Because these cells have a very thin cytoplasm, the boundary between the cell periphery and the perinuclear region is not sharply defined. Nevertheless, we consistently observed MT bundles only in the cytoplasm of *Sas4* neuroblasts, whereas in wt and *asp* neuroblasts, MTs are more uniformly distributed forming well-organized MT bundles in wt and poorly organized MT network in asp mutants. The linescan analyses were included to better illustrate these differences.
The differences in tubulin signal observed between Figure 1A and Figure 1D arise from the use of different anti-tubulin antibodies required for the double staining shown in figure 1D. Importantly, despite this technical difference, the overall MT organization remains consistent across experiments: well-organized MT bundles in WT; MTs predominantly around the nucleus and at the cell periphery in *Sas4* mutant and poorly organized MT network in *asp* mutant NBs. In addition, we wish to clarify that the images shown in Figure 1D were selected from the subset of cells in

© 2026. Published by The Company of Biologists under the terms of the Creative Commons Attribution License (https://creativecommons.org/licenses/by/4.0/).

which MT bundles were occasionally observed near Lamin invaginations (wt 16%, *Sas4* 51,4%, *asp* 50,8%; n>50), in order to illustrate this specific phenotype. **Lines 134-143.**

* In asp mutant NBs, the MT network appears disorganized and consists of short, fragmented MTs" (Figure 1A). In figure1A, a diffuse signal in the cytoplasm and some bundle-like structures at the cell periphery can be observed: what is the argument to conclude on the presence of fragmented MTs? Moreover, according to Garcez et al, sas4/cenpJ deletion result in MT stabilization which is not compatible with fragmentation.
We thank the reviewer for this valuable comment and apologize for the inaccurate use of the term *“fragmented”* to describe the MT network. We agree that this wording may have been misleading. In *asp* mutant neuroblasts, the MT signal is mainly diffuse throughout the cytoplasm, with some peripheral bundles, as correctly noted by the referee. We did not intend to imply physical fragmentation of microtubules. To clarify this point, we have replaced the term *“fragmented”* with *“short and thin”* in the revised text.
Moreover, the z-stacks of Figure 1A, now included as Figure S2, better illustrate that in *asp* mutant NBs, MTs appear thin and short, while in *Sas4* mutant cells MTs are arranged in bundles restricted to the cell periphery rather than forming an integrated network (Figure 1A and Figure S2), consistent with the phenotype described by Basto et al., 2006. In the revised manuscript, we have more clearly described the distinct effects of *asp* and *Sas4* mutations on MT organization. **Lines 98-113.**

*The quantifications (line scans) presented in 1A are poorly informative. For such semi quantitative analysis, reference marks (cell membrane + nuclear envelope markers) should be added to the analysis in order to understand where the MT bundles are located within the cell. In particular, since the authors investigate nuclear morphology and architecture in the following figures, it is crucial to assess the interactions between centrosome/MT and the nuclear envelope in WT versus mutant conditions.
In this line, the DAPI staining unfortunately appears weak: it is difficult to clearly visualize the nuclear boundaries in the figure. Maybe zooming on the nuclei of interest, as done in other figures, would be helpful.
We thank the reviewer for these helpful comments. We agree that including reference markers facilitates the interpretation of MT bundle localization within the cells. In the revised Figure 1A and D we have therefore added reference marks indicating the nuclear envelope, which allow a clearer spatial assessment of MT organization. In addition, we improved the DAPI channel to better visualize nuclear boundaries and provided enlarged views of the nuclei of interest, as suggested. These modifications enhance the clarity of the data and more clearly illustrate the relationship between centrosomes/MT bundles and the nuclear envelope in both WT and mutant neuroblasts.

* In other experimental models, actomyosin cytoskeleton has been shown to be tightly associated with the nucleus and involved in mechano-transduction pathways (Davidson et al, 2021 doi.org/10.1016/j.tcb.2020.11.009). Acto-myosin defects have been documented in Drosophila asp mutant brain cells (Rujano et al., 2013 DOI: 10.1038/ncb2858) and Actin-based deformations of the nucleus have recently been shown be involved in mouse multi-ciliated ependymal cell differentiation (Basso et al, 2025 doi.org/10.1016/j.devcel.2024.11.008). In the light of this literature, it would be important to assess the role of actin cytoskeleton in the processes described in the present manuscript.
Is acto-myosin network organization altered in asp and sas4 mutant cells? If yes, what is its contribution to the nuclear architecture?
We thank the reviewer for this insightful comment and for drawing our attention to the observations reported in Rujano et al. (2013). We fully agree that the actomyosin cytoskeleton is a key regulator of the coupling between cytoskeletal organization, nuclear mechanics, and gene expression. Considering the reviewer’s remark and the existing literature, we indeed expect that defects in the actin-myosin network might be present in *asp* mutant neuroblasts, consistent with the alterations described by Rujano et al., and possibly also in *Sas4* mutants.
To directly address this point, we performed immunofluorescence staining of mutant and control larval brains using anti-myosin, to mark the acto-myosin network, and tubulin with anti-tubulin antibodies. As expected, we observed clear alterations of the actin-myosin network in both *asp* and *Sas4* mutants. These defects suggest an imbalance between actin- and microtubule-generated forces that may influence nuclear morphology and downstream transcriptional

© 2026. Published by The Company of Biologists under the terms of the Creative Commons Attribution License (https://creativecommons.org/licenses/by/4.0/).

responses. These observations are now included in Supplementary Figure 3A and B and their implications are further addressed in the Discussion of the revised manuscript. **Lines 113-122 and Lines 360-364.**
We would like to emphasize that our analysis of actomyosin organization is based on brain squash preparations, which constitute the experimental approach of this study. While this technique does not yield high-resolution images and does not allow visualization of the three-dimensional distribution of myosin, it nevertheless enables reliable detection of changes in actomyosin organization. Despite these technical limitations, the differences in myosin distribution observed in *asp* and *Sas4* mutant cells compared with controls are clear, consistent, and reproducible across independent experiments.

Main concern 2. Nucleus morphology

* In addition to the effect of Asp and sas4 mutations on nucleus invaginations, is there any effect on the nucleus size? This point should be addressed, since the figure presented (figure 1A and E) suggests that the nuclei in sas4 mutant are smaller than in other conditions.
We thank the reviewer for this insightful comment. We have analysed nuclear size in wild-type and mutant brain cells by measuring the DAPI-stained area and performing the appropriate statistical analysis. In agreement with the referee’s observation, our graphical representation confirms that the nuclei of *asp* and *Sas4* mutant brain cells are smaller than those of controls. However, we consider this measurement to have limited biological relevance, as nuclear size can be influenced by multiple factors, particularly in a highly proliferative tissue such as the *Drosophila* larval brain. For this reason, we have chosen not to include this figure in the manuscript, while providing it here for the referee’s consideration and completeness of information.

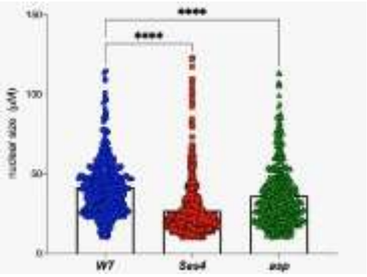

In addition, the authors should clarify the method they use to identify NB Nucleus. Neuroblasts are identified according to the nucleus size (diameter > 10 µm), I have no objection except that this method is not justified in the text. A reference should be added, or supp data should be provided to validate their shift from Deadpan staining to nucleus size estimation.
We thank the reviewer for raising this important point. In the revised manuscript, we have added a Supplemental Figure (S1) showing the nuclear diameter of Deadpan-positive neuroblasts, which validates the use of nuclear size (diameter >10 µm) as a reliable criterion for NB identification.

* In Figure 1B. Wild type, Klar decorates nucleus envelope « while Sas4 and asp mutant cells showed Klar dispersed throughout the nucleus (Fig. 1B and C).
This point should be clarified since it appears to me that the signal is homogenously distributed in the nucleus and in the cytoplasm and not only in the nucleoplasm: Is this protein improper localization or reduced expression? A western blot analysis would be informative (as in figure 1G).
We thank the reviewer for this valuable comment. To address this point, we performed Western blot analyses using two different anti-Klar antibodies (anti Klar-C and anti Klar-M). As shown in the new Supplemental Figures 4C and D, Klar total protein levels in mutant larval brain extracts are comparable to those in wild- type samples, indicating that the altered Klar distribution observed in mutant cells reflects mis localization rather than reduced expression. **Line 129-130.**
We also would like to inform the reviewer that an additional experiment using the alternative anti-Klar antibody (Klar-M) fully corroborated the Klar-C results, confirming Klar dispersion in mutant cells. Because Klar-M provided clearer labelling, these images and quantification are now shown in Figure 1B and C, with Klar-C moved to Supplementary Figure 4A and B.

© 2026. Published by The Company of Biologists under the terms of the Creative Commons Attribution License (https://creativecommons.org/licenses/by/4.0/).

* On nuclear envelope function
Page 6 "Loss of Asp and Sas4 (..) leads to nuclear envelope dysfunction in larval brain cells"
What do the authors mean by Nuclear envelope dysfunction: what are the processes affected? (The global reduction of heterochromatin marks shown in this section could indeed rely on mechanisms that are independent of the nuclear envelope). This point should be clarified.
We thank the reviewer for this comment. We agree that the term “dysfunction” was not appropriate and have replaced it with “defect” throughout the text to more accurately describe the observed nuclear envelope abnormalities.

As far as Cid clustering is concerned, this process is described to occur at prophase, thus to my understanding, when the nuclear envelope starts to be dismantled: how does this process reflect nuclear envelope activity? The authors should argument more precisely on this point.
We thank the reviewer for this observation. We have clarified in the figure legend of the revised manuscript that our analysis was performed on cells in late G2 to early prophase, when the nuclear envelope is still fully intact. **Line 861.**

Page 7 “Taken together, these data indicate that loss of either Sas4 or Asp reduces Lamin levels and disrupts lamin nucleoskeleton architecture, potentially impairing nuclear envelope integrity” Such conclusion raises the question of nuclear integrity (absence of discontinuities / breaks) in the mutants. Nuclear integrity can be easily assessed by the presence of nuclear blebs (see for instance Maning et al 202 DOI :10.1080/19491034.2025.2486816), or by visualizing discontinuities in the lamin staining. It would be interesting to analyse this parameter to support this conclusion.
We thank the reviewer for this insightful comment. Analysis of mutant cells did not reveal the presence of nuclear blebs or discontinuities in the Lamin signal. We agree with the reviewer that the expression “disrupts nuclear envelope integrity” may not be the most appropriate wording, as the observed invaginations do not necessarily indicate breaks in the nuclear envelope. Accordingly, this sentence has been revised in the manuscript to better reflect our observations. **Lines 227-229.**

* In the graphical abstract
-The MT network should be differently drawn in the mutant panel (bundles associated with the nucleus envelope or anything reflecting the phenotypes observed)
- Lamin invaginations are represented under an undeformed nuclear envelope: the nuclear envelope should lie on the lamin network and be curved accordingly.
-In addition, a unique and large invagination seems to be present most of the time in the mutants This is what should be drawn instead of the multiple and tiny "wrinkles".
We have revised the graphical abstract in accordance with the referee’s suggestions.

Main concern 3. Heterochromatin remodelling

*The authors propose that asp and sas 4 mutations "affect heterochromatin organization" (title page 6). Nevertheless, the data presented in the brain cells indicate a global reduction of heterochromatin marks (Immunostaining + Western blot) rather than a spatial reorganization in the nucleoplasm.
We thank the reviewer for this observation. Accordingly, we have revised the title to state that *asp* and *Sas4* mutations lead to a loss of heterochromatin marks, as evidenced by decreased levels of heterochromatin- associated marks detected by immunostaining and Western blot analysis. **Lines 152-153.**

Moreover, in Figure 2 H3K9me3 and 2 signals in the mutants remain in the periphery of the nucleoplasm, as in WT: the spatial distribution of the remaining marks appears thus unchanged. Confusingly, the spatial distribution of H3K9me3 shown in figure 3, does not appear to be consistent with the figure 2. A clarification on this point is thus required.
We thank the reviewer for this insightful comment. We have clarified in the revised manuscript that *asp* and *Sas4* mutants show a reduction in heterochromatin mark levels (H3K9me3 and HP1) without major changes in the peripheral localization of the remaining heterochromatin. **Lines 168-169.**

© 2026. Published by The Company of Biologists under the terms of the Creative Commons Attribution License (https://creativecommons.org/licenses/by/4.0/).

The apparent difference between Figures 2C and 3A reflects the analysis of distinct cell types: neuroblasts in Figure 2 and differentiated brain cells in Figure 3, as indicated in the figure legends. The spatial distribution of heterochromatin differs markedly between neuroblasts and differentiated brain cells. Specifically, neuroblasts exhibit a single heterochromatin mass localized at the nuclear periphery (Figures 2C) whereas differentiated cells show multiple heterochromatin blocks distributed across both peripheral and internal nuclear regions (Figures 3A). To improve visualization of heterochromatin localization within the nuclei of differentiated cells, we have introduced merged images in figure 3. This point has been clarified in the revised manuscript. **Lines 172-176.**

The authors claim that "in mutant brains, the nuclear envelope invaginations were consistently associated with a marked reduction of these heterochromatic marks "(Figure 3A and B). The cells selected and presented in the insets do not support an "association" -in the sense of " co-localization"- of invaginations with a local reduction of the H2K9me2 and me3 marks. A clarification on this point is required too.
We are very grateful to the reviewer for highlighting this important point, which was also noted by Reviewer1. We wish to clarify that H3K9me3 was quantified at the whole-cell level, distinguishing cells with Lamin invaginations from those without, rather than specifically at the invagination sites (Figure 3A, B). We apologize to the reviewer for the misleading wording in our original statement. In the revised manuscript, we now explain that in mutant brains co-stained for LaminDm0 and H3K9me3, cells with clear envelope invaginations consistently displayed lower overall H3K9me3 levels compared to mutant cells without invaginations (Figure 3B), indicating that reduced H3K9me3 occurs at the whole-cell level and is not restricted to nuclear envelope invaginations sites. **Line 177-182.**

In conclusion, the spatial distribution of the chromatin marks should be analysed quantitatively more deeply in the brain cells (distribution area at the periphery as done for the cid, signal distance to invagination etc) in order to support any conclusion on heterochromatin organization.
We thank the reviewer for this valuable suggestion. Quantitative analysis of chromatin spatial distribution is indeed challenging in this context, as the strong reduction of heterochromatic regions in mutant cells precludes reliable measurements. Moreover, analysis of chromatin dynamics is beyond the resolution and capabilities of the experimental tools employed in this study. Differently, quantification of Cid localization was feasible because Cid signal intensity and overall abundance were comparable between wild-type and mutant cells, and because Cid appears as discrete, well-defined foci whose spatial distribution can be robustly assessed.

*As far as results obtained with salivary glands. I understand the interest of the model for the pattern analysis, but I must confess that I do not see the link with brain development "These changes in the DNA banding pattern further support our hypothesis that Asp and Sas4 loss disrupts chromatin organization, potentially altering gene expression programs during brain development" (page 6). This point maybe more argumented in the text.
We are very grateful to the reviewer for highlighting this important point. The analyses performed on salivary gland polytene chromosomes were aimed at gaining a deeper understanding of the chromatin alterations observed in mutant brain cells. Although these tissues differ in morphology and function, previous studies have shown that chromatin organization and its large-scale architectural features are highly conserved between polytene chromosomes and diploid nuclei in other *Drosophila* tissues (Vatolina et al., 2011; Demakov et al., 2011; Eagen et al., 2015). Therefore, the salivary gland model is considered a useful system to visualize and interpret global changes in chromatin organization that may also occur in the CNS. In the revised manuscript, we have expanded these reasons to clarify the relevance of these analyses to our study. **Lines 251-254.**

*Finally, H3K9me3 and 2 levels appear to be different in asp and sas4 mutants (figure 2): could the authors comment on that point?
We thank the reviewer for this comment. *asp* and *Sas4* mutants share many features, yet minor differences, including those in H3K9me2/3 levels, are plausible and consistent with their independent genetic nature.

Minor concerns

© 2026. Published by The Company of Biologists under the terms of the Creative Commons Attribution License (https://creativecommons.org/licenses/by/4.0/).

* Figure 6 Legend
Each dot represents a single polytene chromosome (n ≥ 15).
The exact value of n and the number of replicates should be provided.
We thank the reviewer for the suggestion. We indicated *n ≥ 15* because the exact number of polytene chromosomes analysed may slightly vary among genotypes. Therefore, we reported the lowest value as a conservative estimate, while in all other cases *n* was higher. Each dot in the graph represents one analysed polytene chromosome, and thus the total number of dots corresponds to the *n* for each genotype. We now included the exact *n* values for each genotype and the number of independent experiments in the figure legend.

* Figure 6E
The linescan profiles are confusing: A normalization of the K27 signal intensity on DAPI signal should be more informative. In addition, the x and y axes are not properly labelled.
We thank the reviewer for this helpful suggestion. We have now presented also the DAPI and H3K27me3 intensity profiles for *wt*, *Sas4*, and *asp* separately. As the reviewer rightly noted, this approach provides a more informative and direct comparison between DNA and the H3K27me3 signals.
In the revised version the axes in Figure 6E have been properly labelled.
* Figure 7
"Previous work has shown that the human orthologues of Sas4 and asp, (McIntyre et al. 2012; Williams et
al., 2015) (...) function but also to prevent DNA damage."
The references are not properly quoted: the two publications are based on mouse models not on human. In addition, Drosophila sas4 deletion associates with increased DNA damage in (Poulton et al 2015, DOI:10.1016/j.devcel.2014.08.007), this publication could be mentioned.
We are grateful to the reviewer for pointing out this impreciseness. In the revised manuscript the suggested references have been added. **Line 262.**

Fig.7 B " Graphical representation of the percentage of γH2Av positive cells per field, as shown in (A). n≥35 for 3 brains." The exact value of n should be provided.
We thank the reviewer for the suggestion. We indicated *n ≥ 35* because the exact number of fields analysed may slightly vary among genotypes. Therefore, we reported the lowest value as a conservative estimate, while in all other cases *n* was higher. Each dot in the graph represents one analysed field, and thus the total number of dots corresponds to the *n* for each genotype. We now included the exact *n* values for each genotype in the figure legend.

Second decision letter

MS ID#: dev.205125R1

MS TITLE: Microcephaly-Associated Genes asp and Sas4 Control Chromatin Organization and Nuclear Lamina Structure in Drosophila melanogaster

AUTHORS: Degisew Yinur Mengistu, Marta Marzullo, Claudia Pellacani, Marcella Marchetti, Marta Terribili, Emma Montivero Morales, Maria Patrizia Somma and Laura Ciapponi

Dear Laura,

I have now received all the referees reports on the above manuscript, and have reached a decision. The referees' comments are appended below.

The overall evaluation is positive and we would like to publish a revised manuscript in Development, provided that the referees' comments can be satisfactorily addressed. Please attend to the remaining comments from reviewer 2 which mostly require clarification in the text, better images in a few instances or quantification (eg. Myosin2). If you do not agree with any of their criticisms or suggestions explain clearly why this is so. If it would be helpful, you are welcome to

© 2026. Published by The Company of Biologists under the terms of the Creative Commons Attribution License (https://creativecommons.org/licenses/by/4.0/).

contact us to discuss your revision in greater detail. Please send us a point-by-point response indicating your plans for addressing the referees’ comments, and we will look over this and provide further guidance.

Reviewer 1

Advance summary and potential significance to field

In the revised version of the manuscript the authors included a number of useful experimental clarifications that certainly make the main conclusions of the paper more solid. They have addressed my concerns and clarified several important points, as for example with respect to the assessment and quantification of NE invaginations in the sas4 and asp mutant neuroblasts.

Comments for the author

They also included an epistasis experiment where they restored H3K9 methylation in aspRNAi cells by overexpressing the Metyhylase Su(var)3-9, without affecting the NE phenotypes. While this experiment is useful in placing Histone modifications downstream of asp, in my view it may contradict the results obtained by the Methylstat treatment (Figure 3C, D), where the authors report a rescue of the H3K9 methylation AND the NE irregularities due to loss of function mutations of asp or sas4. I would encourage the authors to negotiate this remaining issue in the discussion. Otherwise I am happy to recommend the revised manuscript for publication.

Reviewer 3

Comments for the author

The text has been amended. Additional experiments have been performed to address some reviewers' comments. Nevertheless, in my opinion, some implementations still need to be provided for the manuscript to be accepted for publication in Development (concerns listed below).

A major question is the specificity of the phenotypes described in the mutants. To address this point, the authors have now performed new experiments by analyzing spd2/CEP192 mutants (Cep192 being not in the list of the MCPH-associated genes): "spd2 mutants display defects reminiscent of those observed in asp and Sas4 mutants" . Line 304. "These findings suggest that disturbance of centrosome integrity or MT organization can broadly affect chromatin state and nuclear architecture."
This conclusion is moreover in line with the new TAXOL data added that indicate that stabilizing microtubules is sufficient to induce nuclear invaginations.

Nevertheless, sas and asp are still in the revised manuscript presented as the key regulators of the processes described:
Title : Microcephaly-Associated Genes asp and Sas4 Control Chromatin Organization and Nuclear Lamina Structure in Drosophila melanogaster)
Abstract : These findings point to a novel potential role for Asp and Sas4 in coordinating centrosome function with chromatin organization, providing new insight into MCPH pathogenesis.
(and other occurrences in the result section)

The coexistence of these statements with the new set of data leads to an ambiguity or confusion that the authors should carefully clarify.

Concern 1: Cytoskeleton organization

1-The description of the microtubule networks in the result section has been improved. Importantly, the new images corresponding to individual z-stack and presented in Supp figure 2 are of better quality to support the description.

© 2026. Published by The Company of Biologists under the terms of the Creative Commons Attribution License (https://creativecommons.org/licenses/by/4.0/).

Surprisingly, these images are relegated in a supp figure and the lower quality ones (z stack projections ?) maintained in the main figure.
The main figure should be revised and integrate the supp figure images.
In addition, line scans should performed on the individual z-stacks and replace the actual ones.

(moreover, significantly thinner dashed lines should be added in the main figure to visualize nucleus boundaries)

2- The contribution of actomyosin network has been now studied, using an antibody against the non-muscle myosin 2 heavy chain protein.

The authors describe "marked defects in actin-myosin organization in both mutant brain cells" (supp figure 3).And, in their answer to the reviewers: "the differences in myosin distribution observed in asp and Sas4 mutant cells compared with controls are clear, consistent, and reproducible across independent experiments."
According to me, the images presented in supp fig 3 do not support these claims.
I have difficulties to detect any clear structure, like a cell cortex?, labeled in the WT or in the mutants. I do not see any significant differences in the red channel between the WT and mutants.

First, higher quality images should be presented.
Importantly, in the legend, the authors omit to mention the type of images they used: are these selected z-stacks, z-projections, non-confocal images?
I suspect that , akin for the microtubule panels, using selected z-stacks of confocal images may increase the quality of the figure.
In addition, a quantitative analysis (Cf microtubules) should be provided to potentially support their conclusion.

Minor comment :
Line 289 "In this context, Sas4 and Asp may support these processes by maintaining MT integrity"
Please use Organization instead of Integrity

Concern 2. Nucleus morphology

I acknowledge the fact that new experimental data have been provided. Nuclear areas have been measured, WB analysis of Klarsicht has been added, indicating that the protein levels are not changed in the mutants (supp fig S4). Lamin C immunostaining as well as Immunostaining data using an alternative antibody against Klarsicht have been added and appear in supp figure 4.

Nevertheless,
1- concerning Klar localization:

Upon close examination of figure 1B, Klar appears to me enriched at the cell cortex and not at the nucleus membrane as proposed by the authors.
A new figure should be proposed with convincing immunostaining data (of note, in the new Supp figure 4 the presence of Klar C at the nuclear membrane may be more visible).
In addition, in order to support the " dispersion of Klar in the mutants" a quantitative analysis must be provided (even a line scan using DAPI as a reference to delineate the nucleus would be informative).

2-concerning the nuclear invaginations:

According to me, the actual Figure 1 D (merge and insets) does not support the presence of microtubules in the nuclear invaginations. A new figure should be proposed. I expect the use of z stacks of confocal images to be helpful to support the authors claim. Moreover, a simple line scan in the two channels would give a valuable taste of quantitative analysis to these data.

In addition, Figure 1 D has been awkwardly modified: dashed lines have been added to visualize nucleus boundaries, but they rather mask the lamin signal (in WT especially).

As a related and minor concern:

© 2026. Published by The Company of Biologists under the terms of the Creative Commons Attribution License (https://creativecommons.org/licenses/by/4.0/).

Starting Line 134
"Lamin signal extending into the nucleoplasm in the form of inward projections or streams "
"invaginations" may be more accurate that projections or streams.
(...) "indicating that Lamin invaginations are not restricted to a specific Lamin isoform, but rather reflects an alteration of the nuclear membrane where Lamin proteins are associated".
Lamin in most of the case is a reasonable read-out for nucleus shape. And lamin isoforms are not known to change shape independently.

3- concerning nucleus integrity:

The authors still state that the "nuclear envelope integrity is not impaired" (line 228).
I agree on the absence of "presence of nuclear blebs "in the figures presented , I would be rather cautious on the absence of "discontinuities in the Lamin signal ». Discontinuities can -according to me - be observed in Fig 1 , including in the wild type. Such discontinuities are likely due to the quality of the immunostaining/imaging. Nevertheless, I suggest to suppress any reference to membrane integrity … or provide a clear quantification to support this claim.

4- concerning nucleus size :

The authors now provide a measure of the nucleus area and conclude that "the nuclei of asp and Sas4 mutant brain cells are smaller than those of controls." but "consider this measurement to have limited biological relevance, as nuclear size can be influenced by multiple factors".

I am surprised since, as far as I understood, nucleus size is an important parameter to consider when you are dealing with nucleus architecture and chromatin organization (Curr Opin Cell Biol. 2014 Feb 4;0:16-27. doi: 10.1016/j.ceb.2014.01.003).

On a more practical point of view, this difference raises the question of the pools of cells that are analyzed and compared in the study.
The new dot plot presented suggests that an important part of the mutants exhibit a nucleus with diameter close to 10µm. As clarified in the present manuscript, neuroblasts are identified according to the nucleus size with a diameter > 10 µm.
Then , can the authors exclude the fact that they introduce a bias in their analyses by excluding in the mutants a sub population of cells with a smaller nuclei? (or does it reflect a shift of the differentiation in the mutants and a reduced neuroblast pool ?)

5- Do finally microtubules control Nucleus shape ?

The authors have tested for their answer the effect of taxol on nucleus deformation : Taxol dependent Microtubule stabilisation and bundle formation induce nucleus invaginations (I line with for istance Hampoelz et al as mentioned by the authors) : this would be in line with the situation in the sas mutant - provided the fact that the authors can demonstrate the presence of perinuclear bundles in this mutant-

On the contrary the asp mutant exhibits a disorganized microtubule network lacking bundle-like or any robust structures. Paradoxically, their nucleus still does exhibit similar invaginations/ deformations.
The authors should discuss more these apparently opposed effects and maybe discuss how they envision the role of microtubules in nucleus shaping.

Concern 3. Heterochromatin remodelling

Regarding the analysis of heterochromatin marks, satisfactory modifications have been provided to the text. In particular it appears more clearly now that the data presented support a global reduction of heterochromatin marks, rather than a spatial reorganization in the nucleoplasm.

© 2026. Published by The Company of Biologists under the terms of the Creative Commons Attribution License (https://creativecommons.org/licenses/by/4.0/).

Second revision

Author response to reviewers' comments

LINES are referred to the track-changes version of the revised manuscript
We would also like to inform the reviewers that figure legends were shortened to comply with the journal's text length requirements. Only general descriptive text was reduced or removed, while all figure-specific information was fully preserved.

Comments from the Reviewers:

Reviewer 1: SUMMARY OF THE ADVANCE MADE IN THIS PAPER AND ITS POTENTIAL SIGNIFICANCE TO THE FIELD

In the revised version of the manuscript the authors included a number of useful experimental clarifications that certainly make the main conclusions of the paper more solid. They have addressed my concerns and clarified several important points, as for example with respect to the assessment and quantification of NE invaginations in the sas4 and asp mutant neuroblasts.

SUGGESTIONS TO AUTHORS
They also included an epistasis experiment where they restored H3K9 methylation in aspRNAi cells by overexpressing the Metyhylase Su(var)3-9, without affecting the NE phenotypes. While this experiment is useful in placing Histone modifications downstream of asp, in my view it may contradict the results obtained by the Methylstat treatment (Figure 3C, D), where the authors report a rescue of the H3K9 methylation AND the NE irregularities due to loss of function mutations of asp or sas4. I would encourage the authors to negotiate this remaining issue in the discussion. Otherwise, I am happy to recommend the revised manuscript for publication.
We thank the reviewer for this comment. We would like to emphasize that these two approaches induce fundamentally different modifications. Methylstat globally inhibits histone demethylases, broadly restoring chromatin methylation toward physiological levels and rescuing both H3K9 methylation and NE morphology. In contrast, Su(var)3-9 overexpression specifically increases H3K9 methylation in a non- physiological manner and does not restore the proper chromatin balance. Thus, the lack of NE rescue in this case does not contradict the Methylstat results, but rather highlights that global restoration of chromatin methylation, rather than elevation of a single mark, is required for normal nuclear envelope organization. These considerations have been included in the revised discussion. **LINES 351-358**

Reviewer 3: SUMMARY OF THE ADVANCE MADE IN THIS PAPER AND ITS POTENTIAL SIGNIFICANCE TO THE FIELD

SUGGESTIONS TO AUTHORS
The text has been amended. Additional experiments have been performed to address some reviewers' comments. Nevertheless, in my opinion, some implementations still need to be provided for the manuscript to be accepted for publication in Development (concerns listed below).

A major question is the specificity of the phenotypes described in the mutants. To address this point, the authors have now performed new experiments by analysing spd2/CEP192 mutants (Cep192 being not in the list of the MCPH-associated genes): "spd2 mutants display defects reminiscent of those observed in asp and Sas4 mutants". Line 304. "These findings suggest that disturbance of centrosome integrity or MT organization can broadly affect chromatin state and nuclear architecture."
This conclusion is moreover in line with the new TAXOL data added that indicate that stabilizing microtubules is sufficient to induce nuclear invaginations. Nevertheless, sas and asp are still in the revised manuscript presented as the key regulators of the processes described:
Title : Microcephaly-Associated Genes asp and Sas4 Control Chromatin Organization and Nuclear Lamina Structure in Drosophila melanogaster)
Abstract : These findings point to a novel potential role for Asp and Sas4 in coordinating centrosome function with chromatin organization, providing new insight into MCPH pathogenesis. (and other occurrences in the result section)

© 2026. Published by The Company of Biologists under the terms of the Creative Commons Attribution License (https://creativecommons.org/licenses/by/4.0/).

The coexistence of these statements with the new set of data leads to an ambiguity or confusion that the authors should carefully clarify.
We thank the reviewer for this comment. We would like to clarify that the analysis of *spd2/CEP192* mutants was specifically included in response to the reviewer's previous request to determine whether the nuclear phenotypes observed in *asp* and *Sas4* mutants were specific to these genes or could also be reproduced using alternative mutants affecting centrosome or microtubule organization. Our results show that *spd2* mutants display similar nuclear and chromatin defects, supporting the conclusion that centrosome integrity is broadly required to maintain nuclear organization and chromatin homeostasis. Importantly, the association between *CEP192* and microcephaly has already been cited in the manuscript (Guo et al., 2024), further supporting the relevance of this centrosomal component in neurodevelopmental disorders. We emphasize, however, that Asp and Sas4 remain the primary focus of this study due to their established causal link with MCPH and the detailed mechanistic analyses presented here, while the *spd2* experiments serve to demonstrate the broader relevance of centrosome integrity in these processes. We apologize if this point was not sufficiently evident and have clarified the Discussion accordingly.
LINES 398-405

Concern 1: Cytoskeleton organization

1-The description of the microtubule networks in the result section has been improved. Importantly, the new images corresponding to individual z-stack and presented in Supp figure 2 are of better quality to support the description.

Surprisingly, these images are relegated in a supp figure and the lower quality ones (z stack projections?) maintained in the main figure.
The main figure should be revised and integrate the supp figure images.
In addition, line scans should performed on the individual z-stacks and replace the actual ones. (moreover, significantly thinner dashed lines should be added in the main figure to visualize nucleus boundaries)
We thank the referee for this comment and for appreciating our efforts to improve image quality. Indeed, all panels in Figure 1 have been substantially enhanced. To improve clarity, individual channels are now displayed in black and white, while merged images remain in colour to facilitate interpretation of signal overlap. We chose to keep the individual z-stack images in Supplementary Figure 2 to avoid overloading the main figure and maintain its readability. Supplementary figures are intended to provide direct visual support for the main data without introducing additional concepts, and readers can easily refer to them for detailed inspection.
Regarding the suggestion to perform line-scans on individual z-stacks, we believe this would likely not provide additional insight and might compromise the spatial context captured in the current projections, as line-scans on maximum-intensity projections are more informative than on single z stacks.
Finally, as requested, the dashed lines outlining nuclear boundaries have been made significantly thinner to improve their visualization.

2- The contribution of actomyosin network has been now studied, using an antibody against the non-muscle myosin 2 heavy chain protein.

The authors describe "marked defects in actin-myosin organization in both mutant brain cells" (supp figure 3). And, in their answer to the reviewers: "the differences in myosin distribution observed in asp and Sas4 mutant cells compared with controls are clear, consistent, and reproducible across independent experiments." According to me, the images presented in supp fig 3 do not support these claims.
I have difficulties to detect any clear structure, like a cell cortex?, labelled in the WT or in the mutants. I do not see any significant differences in the red channel between the WT and mutants. First, higher quality images should be presented. Importantly, in the legend, the authors omit to mention the type of images they used: are these selected z-stacks, z-projections, non-confocal images?
I suspect that, akin for the microtubule panels, using selected z-stacks of confocal images may increase the quality of the figure.
In addition, a quantitative analysis (Cf microtubules) should be provided to potentially support their

© 2026. Published by The Company of Biologists under the terms of the Creative Commons Attribution License (https://creativecommons.org/licenses/by/4.0/).

conclusion.
We thank the reviewer for this valuable comment. We agree that individual actomyosin fibres are not clearly resolved in the images shown in Figure S3; however, the overall distribution of myosin shows notable differences in *asp* and *Sas4* mutant cells. To support this observation quantitatively, we performed line scan analyses of myosin intensity across individual cells (shown in Figure S3A) and across the cell monolayers (shown in Figure S3B). These measurements consistently reveal differences between wild-type and mutant neuroblasts: in wild-type cells, myosin signals are broader and less sharply defined, whereas in *Sas4* and *asp* mutants the signals are narrower and more confined (Figure S3A), indicative of a more disorganized or aggregated pattern. Line-scans performed on brain cell monolayers further revealed a significant reduction in myosin signal in mutants (Figure S3B). Thus, even in the absence of clearly visible fibres, these quantitative analyses provide evidence of altered myosin organization in both mutant genotypes.
To also increase image quality, we added the corresponding z-stacks of the z-projection shown (Figure S3C and D). Finally, the type of images shown has been clearly indicated in each figure legend of the revised manuscript. LINES 118-121

Minor comment:
Line 289 "In this context, Sas4 and Asp may support these processes by maintaining MT integrity" Please use Organization instead of Integrity.
We thank the reviewer for the suggestion; we have included this modification in the revised manuscript.

Concern 2. Nucleus morphology

I acknowledge the fact that new experimental data have been provided. Nuclear areas have been measured, WB analysis of Klarsicht has been added, indicating that the protein levels are not changed in the mutants (supp fig S4). Lamin C immunostaining as well as Immunostaining data using an alternative antibody against Klarsicht have been added and appear in supp figure 4.

Nevertheless,
1- concerning Klar localization:
Upon close examination of figure 1B, Klar appears to me enriched at the cell cortex and not at the nucleus membrane as proposed by the authors.
A new figure should be proposed with convincing immunostaining data (of note, in the new Supp figure 4 the presence of Klar C at the nuclear membrane may be more visible).
In addition, in order to support the " dispersion of Klar in the mutants" a quantitative analysis must be provided (even a line scan using DAPI as a reference to delineate the nucleus would be informative).
We thank the referee for the careful re-examination of the images and for the constructive suggestions.
We would like to clarify that Klar C immunostaining was moved to the Supplementary Figures at the request of another referee, who expressed concerns about the distribution of the LINC protein Klarsicht and considered that the conclusions drawn were not sufficiently supported by the data. To address those concerns, we expanded the analysis using the Klar M antibody and reorganized the presentation of the data accordingly.
In response to the present comment and to further improve clarity, we performed quantitative line-scan analyses using DAPI staining to precisely delineate the nuclear boundary. In wild-type cells, Klar M intensity profiles closely overlap with the DNA-defined nuclear edge. In contrast, in *Sas4* and *asp* mutant cells, the Klar signal is broadened and shifted relative to the nuclear boundary, consistent with a more dispersed distribution. LINES 129-131
Regarding the comment that Klarsicht appears enriched at the cell cortex, we respectfully disagree with this interpretation. In the images shown, no marker of the cell cortex or plasma membrane is present, and cell boundaries are not visualized. Therefore, it is not possible to assign the signal to the cortex based solely on apparent proximity to the cell periphery. Moreover, Klarsicht is a component of the LINC complex, which is known to localize at the nuclear envelope rather than at the plasma membrane (Sosa et al., 2012, https://doi.org/10.1016/j.cell.2012.03.046).

2-concerning the nuclear invaginations:

According to me, the actual Figure 1 D (merge and insets) does not support the presence of

© 2026. Published by The Company of Biologists under the terms of the Creative Commons Attribution License (https://creativecommons.org/licenses/by/4.0/).

microtubules in the nuclear invaginations. A new figure should be proposed. I expect the use of z stacks of confocal images to be helpful to support the authors claim. Moreover, a simple line scan in the two channels would give a valuable taste of quantitative analysis to these data.
In addition, Figure 1 D has been awkwardly modified: dashed lines have been added to visualize nucleus boundaries, but they rather mask the Lamin signal (in WT especially).
We acknowledge the referee's concern about the visualization of microtubules within nuclear invaginations.
In light of this perplexity, we have now removed this image from the revised version of the manuscript. We believe that removal of this image does not alter the main conclusion that mutations in *Sas4* and *asp* induce prominent Lamin-positive nuclear invaginations (Figure 1A). We hope that this revision addresses the referee's concern and improves the overall clarity of the figure presentation.

As a related and minor concern:
Starting Line 134
"Lamin signal extending into the nucleoplasm in the form of inward projections or streams "
"invaginations" may be more accurate that projections or streams.
(…) "indicating that Lamin invaginations are not restricted to a specific Lamin isoform, but rather reflects an alteration of the nuclear membrane where Lamin proteins are associated".
Lamin in most of the case is a reasonable read-out for nucleus shape. And lamin isoforms are not known to change shape independently.
We thank the reviewer for these observations. We have now defined invaginations as deformations of the nuclear envelope in which the nuclear lamina folds inward into the nuclear interior. Accordingly, we consistently use the term "invagination" throughout the manuscript and have added this definition for clarification, as also requested by another reviewer. In addition, the sentence *"indicating that Lamin invaginations are not restricted to a specific Lamin isoform but rather reflect an alteration of the nuclear membrane where Lamin proteins are associated"* has been revised. The text now states that the observed invaginations support the conclusion that these structures reflect deformation of the nuclear envelope, as Lamin staining provides a reliable read-out of nuclear shape. LINES 137-138

3- concerning nucleus integrity:

The authors still state that the "nuclear envelope integrity is not impaired" (line 228).
I agree on the absence of "presence of nuclear blebs "in the figures presented, I would be rather cautious on the absence of "discontinuities in the Lamin signal ». Discontinuities can -according to me - be observed in Fig 1, including in the wild type. Such discontinuities are likely due to the quality of the immunostaining/imaging. Nevertheless, I suggest to suppress any reference to membrane integrity … or provide a clear quantification to support this claim.
We thank the reviewer for this comment. We have removed all statements referring to preserved nuclear envelope integrity and revised the text to more cautiously describe these phenotypes as alterations in Lamin organization and nuclear envelope morphology.

4- concerning nucleus size :

The authors now provide a measure of the nucleus area and conclude that "the nuclei of asp and Sas4 mutant brain cells are smaller than those of controls." but "consider this measurement to have limited biological relevance, as nuclear size can be influenced by multiple factors".
I am surprised since, as far as I understood, nucleus size is an important parameter to consider when you are dealing with nucleus architecture and chromatin organization (Curr Opin Cell Biol. 2014 Feb 4;0:16-27. doi: 10.1016/j.ceb.2014.01.003).
On a more practical point of view, this difference raises the question of the pools of cells that are analyzed and compared in the study.
The new dot plot presented suggests that an important part of the mutants exhibit a nucleus with diameter close to 10µm. As clarified in the present manuscript, neuroblasts are identified according to the nucleus size with a diameter > 10 µm.
Then, can the authors exclude the fact that they introduce a bias in their analyses by excluding in the mutants a sub population of cells with a smaller nuclei? (or does it reflect a shift of the differentiation in the mutants and a reduced neuroblast pool?)
We thank the reviewer for raising this very important point. Neuroblasts in our analyses were

© 2026. Published by The Company of Biologists under the terms of the Creative Commons Attribution License (https://creativecommons.org/licenses/by/4.0/).

identified based on Deadpan positivity and nuclear size, with a minimum diameter of ≥ 10 μm. Importantly, Deadpan- positive cells in both control and mutant brains consistently exhibited nuclear sizes at or above this threshold, indicating that our neuroblast population was not biased by excluding smaller nuclei.

We would also like to clarify that the dot plot shows nuclear area (μm^2). A nucleus with a 10 μm diameter corresponds to an area of approximately 78 μm^2. When applying this threshold, the overall distribution reveals a marked reduction in the proportion of large nuclei in *asp* and *Sas4* mutant brains compared to wild type (*asp*: 0.08%; *Sas4*: 0.03%; wt: 5.11%). This suggests, as noted by the reviewer, a reduced neuroblast pool and a relative increase in smaller, differentiated cells. This interpretation is consistent with a potential increase in differentiation in these mutants; however, as this remains speculative, we have not included this interpretation in the manuscript and limit it to this response.

5- Do finally microtubules control Nucleus shape ?

The authors have tested for their answer the effect of Taxol on nucleus deformation : Taxol dependent Microtubule stabilisation and bundle formation induce nucleus invaginations (In line with for instance Hampoelz et al as mentioned by the authors): this would be in line with the situation in the sas mutant - provided the fact that the authors can demonstrate the presence of perinuclear bundles in this mutant- On the contrary the asp mutant exhibits a disorganized microtubule network lacking bundle-like or any robust structures. Paradoxically, their nucleus still does exhibit similar invaginations/ deformations.

The authors should discuss more these apparently opposed effects and maybe discuss how they envision the role of microtubules in nucleus shaping.

We thank the reviewer for raising this important point. At first glance, the phenotypes observed in *Sas4* and *asp* mutant NBs may appear contradictory, as both exhibit nuclear invaginations despite having different microtubule organizations. We agree with the reviewer that these are only apparently opposite effects. In *Sas4* mutant NBs, centrosomes are absent and microtubules fail to form a radial centrosome-derived network. Instead, they organize into bundles at the cell cortex and around the nucleus, which may exert localized mechanical forces on the nuclear envelope. In *asp* mutant NBs, centrosomes are retained during interphase and microtubules are initially nucleated from them, but the network is highly disorganized and composed of short, thin filaments. This disorganization may lead to an uneven distribution of cytoskeletal forces, which could also promote local deformations of the nuclear envelope.

Together, these observations indicate that nuclear shape is governed by the overall organization of the microtubule network, and that defects arising from different perturbations of this network can lead to similar outcomes by altering the balance of forces acting on the nucleus. We have now briefly incorporated these considerations into the discussion of the revised manuscript. LINES 313-316

Concern 3. Heterochromatin remodelling

Regarding the analysis of heterochromatin marks, satisfactory modifications have been provided to the text. In particular it appears more clearly now that the data presented support a global reduction of heterochromatin marks, rather than a spatial reorganization in the nucleoplasm.

We thank the referee for this positive assessment. We are pleased that the modifications have clarified this point and improved the presentation of our findings.

Third decision letter

MS ID#: dev.205125R2

MS TITLE: Microcephaly-Associated Genes asp and Sas4 Control Chromatin Organization and Nuclear Lamina Structure in Drosophila melanogaster

© 2026. Published by The Company of Biologists under the terms of the Creative Commons Attribution License (https://creativecommons.org/licenses/by/4.0/).

AUTHORS: Degisew Yinur Mengistu, Marta Marzullo, Claudia Pellacani, Marcella Marchetti, Marta Terribili, Emma Montivero Morales, Maria Patrizia Somma and Laura Ciapponi

Dear Laura,

I have now received the comments of reviewer 3 and have reached a decision. The referee's comments are appended below.

The overall evaluation is positive and we would like to publish to publish your manuscript. Could you address before moving forward the remaining comments 2 and 3 pasted below. I concur with the reviewer that the Klar labelling is not clearly labelling the nuclear envelope and I should add that what is more striking is a labelling of the cell cortex. Please attend to these comments in your revised manuscript and detail them in your point-by-point response.

Reviewer 3

Advance summary and potential significance to field

Together, the data clearly show that centrosome integrity is required for nuclear architecture in drosophila neuroblasts. This is absolutely relevant and new in the context of the MCPH disorder.

Comments for the author

1-As far as "The Microtubule and actomyosin sections" are concerned, I am satisfied with the modifications provided.

2-As far as Klar localization is concerned, I am still not convinced by the Immunostaining provided:
As indicated by the authors in their answer
"Klarsicht is a component of the LINC complex, which is known to localize at the nuclear envelope rather than at the plasma membrane (Sosa et al., 2012)"
Nevertheless, in figure fig 1 B and in supp fig 4
(1) in the WT cells , as (+) control, the nuclear boundaries should be continuously labeled. They are only poorly and discontinuously labelled.
(2) in the mutants, I maintain that the line scans in figure 1D indicate, in addition to a continuous signal around the nucleus, an signal increase at the cell periphery (that I localize with the limit of the signal background): this does not make sense biologically speaking, but such increase can be observed in the case of poor quality immunostainings (weak specific signals).
Taken together, these points strongly suggest that the immune staining was of low quality and hardly exploitable.

Therefore the authors should either provide a clear and convincing N envelope labelling in the WT to demonstrate the reliability of their immunostaining, or -if this cannot be achieved- this part should be simply removed, the data being not in support of the quality of the manuscript (The absence of KLAR immunostaining will anyway not significantly affect the progression of the manuscript).

3-Minor and final comments:
Supp figure 3 C. and D : scale bar should be added
For the rest of the figures, I suggest to enlarge the scale bars that are admittedly present, but dramatically discrete.

4-I would like to clarify my previous initial comment on text "ambiguities":
The main findings of the manuscript appear in two flavors:
*Flavor (1): see title, or in the introduction (page 3 line 74 to 81)
"… these genes (sas4 and asp) are critical for the maintenance of chromatin architecture and genome integrity during Drosophila neurogenesis.
In this scenario, the effects described are a direct consequence of the mutations. "
*Flavor (2): see Abstract

© 2026. Published by The Company of Biologists under the terms of the Creative Commons Attribution License (https://creativecommons.org/licenses/by/4.0/).

"These findings suggest a novel role for centrosome proteins in regulating chromatin organization, providing new insights into the mechanisms underlying MCPH pathogenesis."
In this scenario, the effects described are an indirect consequence of the mutations, the initial effect is the loss of centrosome integrity that in turn affects nuclear architecture.

My feeling is that the manuscript focuses on flavor (1) while flavor (2) is more accurate. Together, the data clearly show that centrosome integrity is required for nuclear architecture in drosophila neuroblasts. This is absolutely relevant and new in the context of the MCPH disorder.
Shedding more light in the discussion on the central role of "centrosome integrity" would have given more hindsight to the work.

In conclusion, I support the acceptation of the manuscript provided that the minor points 2 and 3 are addressed.

Third revision

Author response to reviewers' comments

Comments from the Reviewers:

Reviewer 3: SUMMARY OF THE ADVANCE MADE IN THIS PAPER AND ITS POTENTIAL SIGNIFICANCE TO THE FIELD

Together, the data clearly show that centrosome integrity is required for nuclear architecture in drosophila neuroblasts. This is absolutely relevant and new in the context of the MCPH disorder.

SUGGESTIONS TO AUTHORS

1-As far as "The Microtubule and actomyosin sections" are concerned, I am satisfied with the modifications provided.

2-As far as Klar localization is concerned, I am still not convinced by the Immunostaining provided:
As indicated by the authors in their answer
"Klarsicht is a component of the LINC complex, which is known to localize at the nuclear envelope rather than at the plasma membrane (Sosa et al., 2012)"
Nevertheless, in figure fig 1 B and in supp fig 4
(1) in the WT cells , as (+) control, the nuclear boundaries should be continuously labeled. They are only poorly and discontinuously labelled.
(2) in the mutants, I maintain that the line scans in figure 1D indicate, in addition to a continuous signal around the nucleus, an signal increase at the cell periphery (that I localize with the limit of the signal background): this does not make sense biologically speaking, but such increase can be observed in the case of poor quality immunostainings (weak specific signals).
Taken together, these points strongly suggest that the immune staining was of low quality and hardly exploitable.

Therefore the authors should either provide a clear and convincing N envelope labelling in the WT to demonstrate the reliability of their immunostaining, or -if this cannot be achieved- this part should be simply removed, the data being not in support of the quality of the manuscript (The absence of KLAR immunostaining will anyway not significantly affect the progression of the manuscript).

We thank the reviewer for this important observation. We agree that the quality of the immunostaining was not sufficiently robust to support a clear and convincing conclusion. Although we were reasonably confident in our interpretation, the antibodies currently available did not allow us to obtain staining of sufficient quality. Therefore, following the reviewer and the

© 2026. Published by The Company of Biologists under the terms of the Creative Commons Attribution License (https://creativecommons.org/licenses/by/4.0/).

editor suggestion, we have removed the Klar immunostaining experiments from the manuscript. We agree that their removal does not affect the main conclusions or the overall message of our study.

3-Minor and final comments:
Supp figure 3 C. and D : scale bar should be added
For the rest of the figures, I suggest to enlarge the scale bars that are admittedly present, but dramatically discrete.
We fixed this issue

4-I would like to clarify my previous initial comment on text "ambiguities": The main findings of the manuscript appear in two flavors:
*Flavor (1): see title, or in the introduction (page 3 line 74 to 81)
"… these genes (sas4 and asp) are critical for the maintenance of chromatin architecture and genome integrity during Drosophila neurogenesis.
In this scenario, the effects described are a direct consequence of the mutations. "
*Flavor (2): see Abstract
"These findings suggest a novel role for centrosome proteins in regulating chromatin organization, providing new insights into the mechanisms underlying MCPH pathogenesis."
In this scenario, the effects described are an indirect consequence of the mutations, the initial effect is the loss of centrosome integrity that in turn affects nuclear architecture.

My feeling is that the manuscript focuses on flavor (1) while flavor (2) is more accurate. Together, the data clearly show that centrosome integrity is required for nuclear architecture in drosophila neuroblasts. This is absolutely relevant and new in the context of the MCPH disorder. Shedding more light in the discussion on the central role of "centrosome integrity" would have given more hindsight to the work.

In conclusion, I support the acceptation of the manuscript provided that the minor points 2 and 3 are addressed.

We thank the reviewer for this clarification. We agree that flavour 2 is more accurate and that some sentences in the previous version may have led to ambiguity. Accordingly, following the reviewer’s suggestion, we have revised the wording by modifying a term in the title and by rephrasing the relevant sentences in the Introduction (page 3, lines 74–83).

Fourth decision letter

MS ID#: dev.205125R3

MS TITLE: Microcephaly-Associated Genes asp and Sas4 influence Chromatin Organization and Nuclear Lamina Structure in Drosophila melanogaster

AUTHORS: Degisew Yinur Mengistu, Marta Marzullo, Claudia Pellacani, Marcella Marchetti, Marta Terribili, Emma Montivero Morales, Maria Patrizia Somma and Laura Ciapponi

Dear Laura,

I looked carefully at your responses to the remaining comments of reviewer 3 and in light of this I am happy to tell you that your manuscript has been accepted for publication in Development, pending our standard publication integrity checks.

© 2026. Published by The Company of Biologists under the terms of the Creative Commons Attribution License (https://creativecommons.org/licenses/by/4.0/).
